# Effectiveness of integrated care including therapeutic assertive community treatment in severe schizophrenia-spectrum and bipolar I disorders: Four-year follow-up of the ACCESS II study

**DOI:** 10.1371/journal.pone.0192929

**Published:** 2018-02-27

**Authors:** Daniel Schöttle, Benno G. Schimmelmann, Friederike Ruppelt, Alexandra Bussopulos, Marietta Frieling, Evangelia Nika, Luise Antonia Nawara, Dietmar Golks, Andrea Kerstan, Matthias Lange, Michael Schödlbauer, Anne Daubmann, Karl Wegscheider, Anja Rohenkohl, Gizem Sarikaya, Mary Sengutta, Daniel Luedecke, Linus Wittmann, Gunda Ohm, Christina Meigel-Schleiff, Jürgen Gallinat, Klaus Wiedemann, Thomas Bock, Anne Karow, Martin Lambert

**Affiliations:** 1 Psychosis Centre, Department of Psychiatry and Psychotherapy, Centre of Psychosocial Medicine, Medical Center Hamburg-Eppendorf, Hamburg, Germany; 2 University Hospital of Child and Adolescent Psychiatry, University of Bern, Bern, Switzerland; 3 University Hospital of Child and Adolescent Psychiatry, University Medical Center Hamburg-Eppendorf, Hamburg, Germany; 4 Department of Medical Biometry and Epidemiology, University Medical Center Hamburg-Eppendorf, Hamburg, Germany; 5 Strategic University Development Centre, University Medical Centre, Hamburg-Eppendorf, Hamburg, Germany; 6 Department of Psychiatry and Psychotherapy, University Centre of Psychosocial Medicine, Medical Center Hamburg-Eppendorf, Hamburg, Germany; Centre for Addictions and Mental Health, CANADA

## Abstract

The ACCESS-model offers integrated care including assertive community treatment to patients with psychotic disorders. ACCESS proved more effective compared to standard care (ACCESS-I study) and was successfully implemented into clinical routine (ACCESS-II study). In this article, we report the 4-year outcomes of the ACCESS-II study. Between May 2007 and December 2013, 115 patients received continuous ACCESS-care. We hypothesized that the low 2-year disengagement and hospitalization rates and significant improvements in psychopathology, functioning, and quality of life could be sustained over 4 years. Over 4 years, only 10 patients disengaged from ACCESS. Another 23 left for practical reasons and were successfully transferred to other services. Hospitalization rates remained low (13.0% in year 3; 9.1% in year 4). Involuntary admissions decreased from 35% in the 2 years prior to ACCESS to 8% over 4 years in ACCESS. Outpatient contacts remained stably high at 2.0–2.4 per week. We detected significant improvements in psychopathology (effect size *d = 0.79*), illness severity (*d = 1.29*), level of functioning (*d = 0.77*), quality of life (*d = 0.47*) and stably high client satisfaction (*d = 0.02*) over 4 years. Most positive effects were observed within the first 2 years with the exception of illness severity, which further improved from year 2 to 4. Within continuous intensive 4-year ACCESS-care, sustained improvements in psychopathology, functioning, quality of life, low service disengagement and re-hospitalization rates, as well as low rates of involuntary treatment, were observed in contrast to other studies, which reported a decline in these parameters once a specific treatment model was stopped. Yet, stronger evidence to prove these results is required.

**Trial registration:** Clinical Trial Registration Number: NCT01888627

## Introduction

Psychotic disorders are among the most severe mental disorders [1, 2]. Patients often remain highly vulnerable to future episodes and experience persisting or even increasing difficulties in functioning even when not acutely ill [2, 3].

Various outpatient care models have been developed for patients with severe mental illness (SMI) to serve their complex treatment needs (e.g., assertive community treatment [ACT] [4–10] and have generally shown positive effects.

Most of these care models are diagnosis-nonspecific and do not offer continuous and unlimited treatment [9]. However, specific and unlimited care may be important for patients with psychotic disorders as they have high rates of service-disengagement and non-adherence and commonly show a chronic course of illness [10, 11].

In 2006, our group designed and evaluated a diagnosis-specific integrated care treatment model including ACT (the ACCESS-model) for patients with schizophrenia-spectrum disorders (SSD) [4, 12]. Briefly, the most salient differences to other ACT-care models are the focus on psychotic disorders (and not on severely ill patients in general), the expertise of the ACT-team in treating psychotic disorders with their focus on psychotherapy and family involvement, and the embedment of the ACT-team in an integrated care program allowing nee-adapted treatment. Compared to a reference catchment area offering standard care, the ACCESS-I-study showed a markedly better 1-year efficacy of the ACCESS-model at costs comparable to standard care. In 2007, the ACCESS-model was implemented in routine care and concurrently extended to patients with bipolar disorder with psychotic features (BD), offering continuous and open-ended treatment. Under real-life conditions, the effectiveness of the program was continuously assessed (ACCESS-II-study) [6]. The 24-month follow-up revealed low service disengagement rates and hospital days as well as improvements in psychopathology, illness severity, functioning level, quality of life, and client satisfaction in both SSD and BD [6]. These improvements were similar to those found in ACCESS under research conditions (ACCESS-I-study [4]).

There is some evidence that specific care models are effective only as long as they are delivered. Although not directly comparable to our study cohort because of the focus on first-episode patients, the OPUS-trial revealed that their care model (including ACT) was superior to standard care with most benefits occurring in the early phase, and stabilized as long as the care was delivered (i.e., for 24 months) [13, 14]. However, after OPUS-care was stopped, the 60-month follow-up showed that patients deteriorated after cessation of OPUS-care to the extent that model and standard care produced similar 60-month outcomes [15].

We report the 4-year outcomes of the original 115 patients with severe SSD and BD treated in the ACCESS-model (ACCESS-II-study). We hypothesized that the low service disengagement rates and the 2-year improvements in psychopathology, functioning, and quality of life would be maintained. We also hypothesized that the frequency of outpatient contacts and medication adherence would remain stably high, and rates of inpatient care and involuntary admissions stably low, over the 4 years of treatment in ACCESS.

## Materials and methods

### Context, sample, inclusion and exclusion criteria

The Psychosis Center of the University Medical Center Hamburg-Eppendorf is responsible for the treatment of adult patients with severe SSD or BD in an urban catchment area of 300,000 inhabitants.

**Fig 1:**
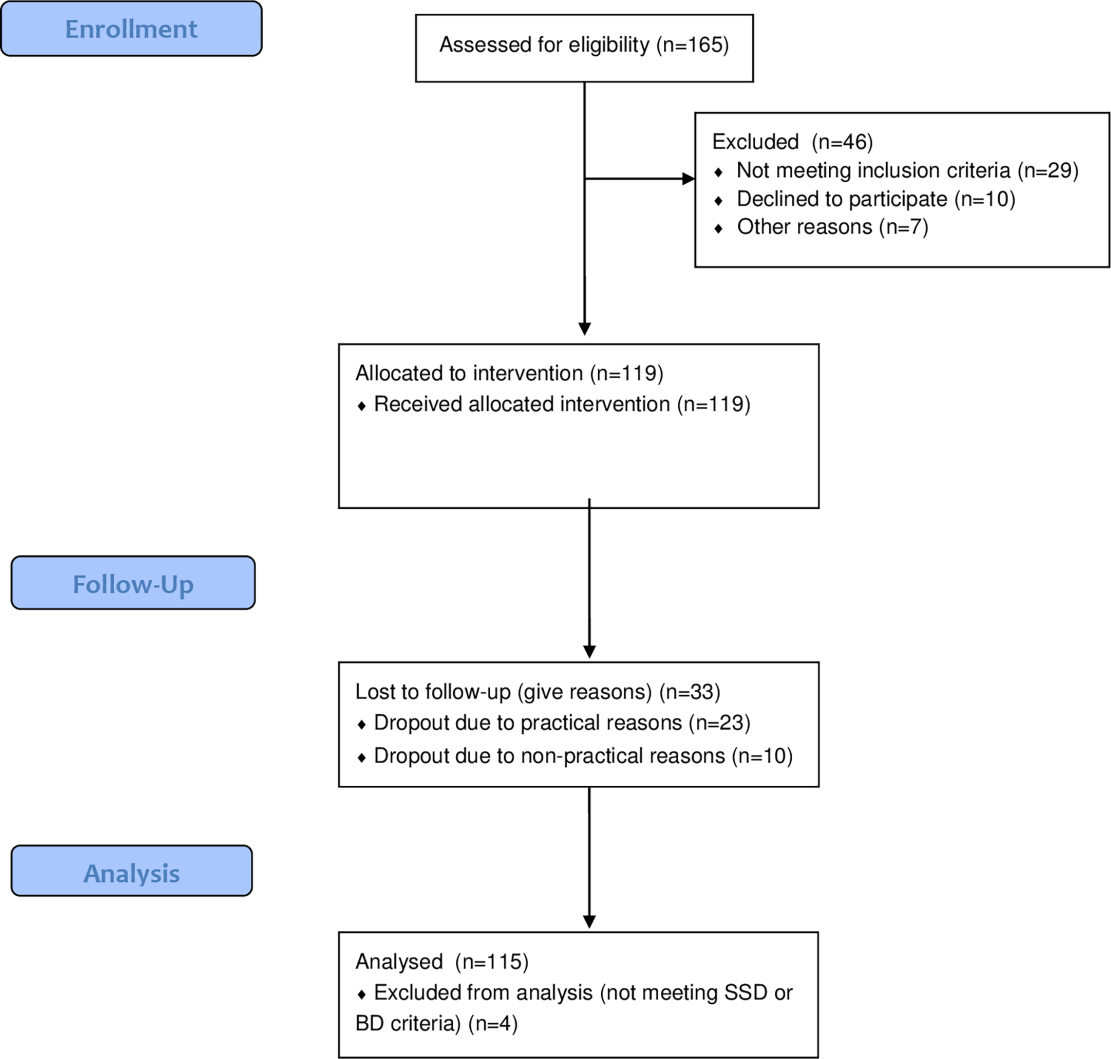
Sample flow chart. Patients of the study were recruited from 1st of May 2007 to 31st of October 2009. 115 patients with SSD or BD and severe mental illness were included for the study. All treated patients (N = 115) participated in the assessments, which were administered as part of the clinical routine.

The ACCESS-model is described in detail elsewhere [4] [6] (see also S2 and S5 Files). The main characteristics of the integrated care concept, including details on ACT, inclusion and exclusion criteria, and assessments are outlined in Tables 1 and 2. From May 2007 to December 2013, 115 patients with SSD or BD and severe mental illness were treated with the ACCESS-model (Fig 1). All treated patients (N = 115) participated in the assessments, which were administered as part of the clinical routine. The investigation was carried out in accordance with the latest version of the Declaration of Helsinki and informed consent of the participants was obtained. All patients treated in the ACCESS model gave informed consent that their data could be used in the context of the ACCESS-II study whenever they were sufficiently stable and the capacity to consent was determined by a consultant psychiatrist. The local institutional review board (Ethikkommission der Ärztekammer Hamburg) approved the observational study (registration number: PV4059). The study was registered at ClinicalTrials.gov (identifier: NCT01888627).

**Table 1:** Characteristics of the ACCESS treatment and inclusion/exclusion criteria.

Characteristics	Content
**Integrated care model**	
**Catchment area with population size**	■ Catchment area of the Department of Psychiatry and Psychotherapy of the University Medical Center, 300,000 habitants
**Health care facilities within the IC model**	■ Specialized psychosis inpatient unit with attached day-clinic; acute inpatient unit (closed ward), specialized psychosis outpatient center, ACT team, specialized day-clinic for first-episode psychosis patients in the age range of 15–29, working support outpatient center, 20 private psychiatrists
**ACT team fidelity**	
**Maximum full-time equivalent caseload**	■ 15–25
**Staff fidelity and skills**	■ Consultant psychiatrists, psychiatrists, psychologists, nurses, social worker
**Staff skills**	■ Diagnosis-specific training in pharmacotherapy, cognitive behavioral (CBT), dynamic, and/or family psychotherapy, pharmacotherapy
**Work style**	■ Shared caseload, patients are discussed in daily team meetings, weekly internal and external supervision, regularly patient-centered network meetings
**Availability**	■ Extended hours (8 am to 6 pm Monday to Friday) & 24-hour crisis telephone & 24-hour emergency service within the Department
**Contact with clients**	■ High frequent face-to-face contacts, assertive engagement, shared-decision making, “no drop-out” policy
**Main interventions**	■ Case management, home treatment, individual, group and family psychotherapy, psychoeducation, pharmacotherapy, social work
**Inclusion and Exclusion Criteria**	
**Inclusion criteria:** **Exclusion criteria:**	■ Diagnosis of a schizophrenia spectrum disorder (i.e., schizophrenia, schizophreniform disorder, schizoaffective disorder, delusional disorder, or psychotic disorder not otherwise specified) or bipolar disorder with psychotic features, all assessed with the Structured Clinical Interview for DSM-IV Axis I Disorders (SCID-I)[16] ■ Aged ≥ 18 years ■ Present hospitalization because of an acute illness state as assessed by a psychiatrist ■ Presence of a certain severity of illness as assessed with the Brief Psychiatric Rating Scale, 24-item version (BPRS),[17] with (1) BPRS total score ≥ 40 points and (2) fulfillment of 1 of the following 8 criteria: ≥ 6 points on item 10 (hallucinations); ≥ 6 points on item 11 (unusual thought content); ≥ 6 points on item 15 (conceptual disorganization); ≥ 10 total points on items 3 and 4 (depressive-suicidal syndrome); ≥ 6 points on item 4 (suicidality); ≥ 15 total points on items 8, 9, and 21 (manic syndrome); ≥ 15 total points on items 6, 12, and 20 (disruptive behavior syndrome); or ≥ 15 total points on items 13, 16, and 17 (negative syndrome). ■ Psychotic disorders due to a medical condition were excluded.

**Table 2:** Assessments and measures.

Assessments and Measures	Details
**Fidelity of the ACT team**	Fidelity of the assertive community treatment model was assessed yearly with the Dartmouth Assertive Community Treatment Scale.[18] At initiation of ACCESS, the total score was 4.5 and varied yearly between 4.2 and 4.6 points, indicating that fidelity of the treatment model was good.
**Fidelity of ratings**	Trained raters independent of the treatment team to avoid bias. All raters received extensive training, particularly for SCID-I interviews, BPRS, CGI-S, and GAF.
**Assessment time points**	Baseline, week 6, and months 3, 6, 12, 18, 24, 30, 36, 40, and 48
**Diagnoses**	Diagnoses of the psychotic disorder and comorbid Axis I disorder(s) were assessed with the SCID-I.[16]
**Service disengagement**	Service disengagement for non-practical reasons was considered to be present if a patient repeatedly refused further treatment despite the need and several attempts at reengagement (phone calls to patient and potentially home visits by the assertive community treatment team).[10]
**Service use data**	Treatment contacts consisted of face-to-face meetings as well as emails/letters, telephone calls, and contact with institutions or family members. Furthermore, hospital days (inpatient and day-clinic treatment) were noted for each year of treatment. All service use data are presented for patients being actively treated in each year (i.e., excluding service-disengaged patients).
**Baseline assessments**	■ Sociodemographic, functional, and pretreatment characteristics using the German version of the Early Psychosis File Questionnaire,[19] ■ Employment/occupation using the Modified Vocational Status Index[20] and the Modified Location Code Index.[20] “Employed/occupied” comprised paid or unpaid full- or part-time employment, being an active student in university, a full- or part-time volunteer; “independent living” comprised living alone, with a partner, or with peers. ■ Duration of untreated psychosis with the Duration of Untreated Psychosis Scale.[21–23] ■ Prevalence of previous inpatient treatment, lifetime involuntary admission, and admission within the 2 years before ACCESS were assessed by interviewing patients, relatives, and health service staff previously responsible for the patient. Data were validated by cross checking the hospital database. Involuntary admissions were due to danger to self or others. ■ Medication adherence was assessed using the criteria of Kane et al.[24] Therapists rated their patients as being fully adherent in the last 4 weeks if taking ≥ 80% of their prescribed medications, partially adherent when taking 20%–80%, and nonadherent when taking ≤ 20% of the prescribed medications.
**Baseline and follow-up assessments**	■ Psychopathology using the BPRS at baseline and every 6 months ■ Severity of illness using the Clinical Global Impressions -Severity of Illness scale (CGI-S)^[^25] ■ Level of functioning using the Global Assessment of Functioning (GAF) Scale[26]; ■ Quality of life using the 18-item Quality of Life Enjoyment and Satisfaction Questionnaire (QLES-Q-18)[27] ■ Patients’ satisfaction with their care using the Client Satisfaction Questionnaire (CSQ-8)^[^28] ■ Medication adherence (see previous paragraph above)^[^24]

### Statistical analysis

Descriptive baseline differences between diagnostic groups were assessed via independent-samples t- tests for continuous dependent variables. There were two Tweedie-distributed variables [29], i.e., with ties at 0 (duration of untreated psychosis and untreated illness), which were evaluated by Mann-Whitney U-tests. Categorical variables were assessed with Chi-square tests. To compare baseline with the 48-month follow-up for the binary outcomes (e.g., involuntary admissions), we used McNemar’s test. We evaluated the changes from baseline (admission) via mixed-model repeated measures, considering the follow-up times as repeated measures, the patients as the random effect, the group (if applicable) and time as fixed effects, and the baseline values of the dependent variable as covariates. Outcomes were changes from baseline for BPRS total-score, CGI-S score, GAF, Q-LES-Q-18, and CSQ-P. We examined the interaction between time and diagnostic group (if applicable). In every model, this interaction was not significant. Therefore, we eliminated the interaction-term from all models. We used the baseline values as covariates to minimize variance [30]. The main effects (F), significance levels (p), and effect sizes (d) are reported. Effect sizes (d) were calculated by dividing the differences of adjusted mean scores by the standard deviation of residuals [31]. The level of significance was set at p < .05, two-sided. Statistical analyses were performed using SPSS Version 20.0 (IBM Corp, 2011).

## Results

### Baseline characteristics

One hundred and fifteen patients with SSD or BD were treated with the ACCESS-model and participated in the ACCESS-II study. Baseline details are displayed in Table 3. Patients with SSD (n = 92) and BD (n = 23) were severely ill (high CGI-S and BPRS scores and low GAF scores). Quality of life and satisfaction with care before entry into the ACCESS-model were low; 43.5% of all patients had involuntary admissions to inpatient treatment in the past, and only 25.2% (n = 28) were adherent to their most recent medication regime. As expected, patients with SSD and BD had similar baseline characteristics except that those with SSD had a longer duration of untreated psychosis, fewer previous suicide attempts, and less insight into illness. Only 15 of 115 patients (13%) had received psychotherapeutic treatment in the 2 years before admission.

**Table 3:** Baseline variables.

	All patients (N = 115)	SSD (n = 92)	BD (n = 23)	p-value
**Demographic details**				
Age, mean (SD)	41.8 (12.9)	41.4 (12.8)	43.6 (13.2)	.47
Sex, n (%), male	51 (44.3)	41 (44.6)	10 (43.5)	.23
Partnership, n (%), single	100 (87.0)	83 (90.2)	17 (73.9)	.04
Education				.48
9 years, n (%)	18 (16.4)	14 (15.9)	4 (18.2)	
10 years, n (%)	37 (33.6)	32 (36.4)	5 (22.7)	
13 years, n (%)	55 (50.0)	42 (47.7)	13 (59.1)	
Completed professional education, n (%)	73 (63.5)	58 (63.0)	15 (65.2)	.65
Employment/occupation, n (%)	22 (18.1)	18 (19.6)	3 (13.0)	.47
Living independently, n (%)	102 (88.7)	80 (87.0)	22 (95.7)	.24
**Illness details**				
First episode psychosis, n (%)	15 (13.0)	14 (15.2)	1 (4.3)	.17
Comorbid psychiatric disorder at entry, n (%)	87 (75.7)	71 (77.2)	16 (69.6)	.45
Substance use disorder (SUD) lifetime, n (%)	62 (53.9)	51 (55.4)	11 (47.8)	.51
Other comorbid disorder lifetime, n (%)	68 (59.1)	55 (59.8)	13 (56.5)	.77
Family history of psychiatric disorder ^a^				
Any psychiatric disorder, n (%)	54 (47.0)	42 (45.7)	12 (52.2)	.78
Psychotic disorder, n (%)	31 (27.0)	23 (25.0)	8 (34.8)	.45
Previous inpatient treatment				
Any inpatient treatment lifetime, n (%)	97 (84.3)	76 (82.6)	21 (91.3)	.21
Involuntary admission, lifetime, n (%)	50 (43.5)	39 (42.4)	11 (47.8)	.52
Involuntary admission, 2 years before, n (%)	40 (34.8)	30 (32.6)	10 (43.5)	.26
Psychotherapy treatment, 2 years before, n (%)	15 (13.0)	10 (10.9)	5 (21.7)	.17
Insight into illness before IC, n (%)	72 (61.0)	51 (55.4)	18 (81.8)	.04
Suicide attempts in the past, n (%)	47 (40.9)	33 (35.9)	14 (60.9)	.03
Forensic history, n (%)	9 (7.8)	8 (8.7)	1 (4.3)	.45
Traumatic adversities				
Any traumatic adversity in the past, n (%)	73 (63.5)	55 (59.8)	18 (78.3)	.14
Traumatic adversities before age 18, n (%)	58 (50.4)	47 (51.1)	11 (47.8)	.93
Duration of untreated illness				
DUI, median in weeks (quartiles)	156.4 (52.3–275.0)	156.6 (56.5–264.2)	104.4 (44.6–373.7)	.67
DUP, median in weeks (quartiles)	21.6 (5.9–52.1)	21.9 (8.4–52.1)	8.4 (0.0–21.9)	.01
Full adherence with last medication, n (%)	29 (25.2)	25 (27.2)	4 (17.4)	.34
**Baseline scores of assessment scales**				
BPRS total score, mean (SD)	81.8 (20.5)	81.3 (19.7)	84.0 (23.8)	.57
CGI-S-score, mean (SD)	5.9 (0.9)	5.9 (0.9)	5.8 (1.1)	.70
GAF-score, mean (SD)	37.0 (12.2)	36.7 (12.2)	38.0 (12.8)	.67
Q-LES-Q-18-score, mean (SD)	2.2 (0.6)	2.2 (0.6)	2.3 (0.8)	.70
CSQ-8 P-score, mean (SD)	1.9 (0.4)	1.9 (0.4)	2.0 (0.4)	.28

**Notes. BD = Bipolar Disorder; SSD = Schizophrenia Spectrum Disorder;** BPRS: Brief Psychiatric Rating Scale, CGI-S: Clinical Global Impression scale—Severity score, GAF: Global Assessment of Functioning scale, CSQ-8 P: Client Satisfaction Questionnaire-8 (patient version), Q-LES-Q-18: Quality of Life Enjoyment and Satisfaction Questionnaire.

^a^ First- and second-degree relatives.

### Service use

Service use data are displayed in Table 4. Within the 48-month treatment period, 72 patients (62.6%) were hospitalized, and 9 patients (7.8%) received day-treatment (including the index admission). Days of inpatient treatment significantly reduced from year 1 (22.4 days, SD = 27.9) to year 4 (4.7 days, SD = 10.6; including first admission). Rates of involuntary admission declined significantly (n = 9 in total, 7.8%) compared to the 2 years before ACCESS (n = 40, 34.8%; McNemar test, *p <* .*001*).

**Table 4:** Service use and service-disengagement during 4 years of treatment.

**Service use during 4 years of treatment**	***Year 1 (n = 115)***	***Year 2 (n = 107)***	***Year 3 (n = 100)***	***Year 4 (n = 88)***
Total number of treatment contacts per week/patient, mean (SD)	2.4 (0.9)	2.4 (1.2)	2.2 (0.9)	2.2 (0.7)
Hospital use				
■ Days of inpatient admissions, mean (SD)	22.4 (27.9)	12.2 (26.9)	3.5 (12.2)	4.6 (10.6)
■ Days day-clinic admissions, mean (SD)	5.5 (25.9)	0	0	0
**Service disengagement during 4 years of treatment**				
■ Reasons for disengagement				
■ moved out of catchment area, n	3	0	3	0
■ moved to sheltered housing, n	1	4	4	1
■ transition to other service, n	1	0	1	2
■ change of health insurance company, n	0	0	1	0
■ change of diagnosis, n	1	1	0	0
■ drop-outs non-practical reasons, n	2	2	3	3

Patients received 2.0–2.4 outpatient treatment contacts per week. This rate was stable over the 4 years. The ACT team conducted most contacts. Eighty-four patients (73.0%) received psychotherapeutic treatment conducted by the ACT team or private psychotherapists. A significantly higher percentage of patients with bipolar disorder than patients with schizophrenia spectrum disorders received psychotherapy (91.3% vs. 68.5%; McNemar test, *p =* .*03*).

### Service-disengagement

Over the 48-month treatment period, 10 patients (8.7%) were service-disengaged after a median of 115.4 weeks (range 16.0–183.9; quartiles 44.0–162.8) (please see Table A and Table B in S1 File). Furthermore, 23 patients (20.0%) dropped out of the study due to practical reasons (e.g., moved out of catchment area) after a median duration of treatment of 104.4 weeks (range 5.9–181.7; quartiles 61.0–134.4).

### Secondary outcomes in SSD and BD

At 48-months, 75.7% (n = 87) were fully adherent (McNemar-test, *p <* .*001*), compared to 25.2% (n = 29) at baseline, with no differences between SSD and BD.

All follow-up assessments during the 4 years indicated improved psychopathology, illness severity, global functioning, and quality of life (Table 5). Larger improvements in psychopathology (*p =* .*003; d =* .*86*) and quality of life (p = .033; *d =* .*45*), as denoted by medium to large effect sizes, were detected in BD, compared to SSD, between baseline and year 4. Significant improvements in test scores occurred during the first 18 months for the BPRS, during the first 6 months for the CGI and GAF, within the first 3 months for the Q-LES-Q-18-, and during the first 6 weeks for the CSQ-8-scores. Further significant improvements after year 2 were only found regarding illness severity (CGI-scores, *p =* .*03*). All other scores remained stable (thus did not deteriorate).

**Table 5:** Course of illness of all patients (N = 115).

		Mean (SD)	Change from baseline	MMRM 48-months follow-up		
			Adjusted mean (95% CI)	Time effect, F	p- value	Effect size, d
Outcomes						
BPRS total score						
Baseline		81.6 (20.4)				
24 months		50.5 (10.4)	-29.7 (-31.7 to -27.7)			
48 months		51.0 (12.8)	-30.5 (-32.7 to -28.3)	7.77	<0.001	0.79
CGI-Severity score						
Baseline		5.9 (1.0)				
24 months		4.2 (1.0)	-1.7 (-1.9 to -1.5)			
48 months		4.0 (1.1)	-1.9 (-2.0 to -1.7)	9.29	<0.001	1.29
GAF						
Baseline		36.9 (12.1)				
24 months		57.4 (13.0)	20.6 (18.5 to 22.7)			
48 months		59.4 (14.7)	22.5 (20.4 to 24.6)	7.31	<0.001	0.77
Q-LES-Q-18						
Baseline		2.2 (0.7)				
24 months		3.2 (0.6)	1.0 (0.9 to 1.1)			
48 months		3.2 (0.6)	1.0 (0.9 to 1.1)	2.55	0.007	0.47
CSQ-8 P						
Baseline		1.9 (0.4)				
24 months		3.3 (0.4)	1.2 (1.2 to 1.3)			
48 months		3.1 (0.5)	1.2 (1.1 to 1.3)	1.04	0.406	0.02

Notes

BPRS: Brief Psychiatric Rating Scale, CGI-S: Global Clinical Impression scale-Severity score, GAF: Global Assessment of Functioning scale, Q-LES-Q-18: Quality of Life Enjoyment and Satisfaction Questionnaire, CSQ-8 P: Client Satisfaction Questionnaire-8 (patient version), SD: Standard Deviation, CI: Confidence Intervall

Thirty patients (27.3%) were employed/occupied after 48 months (vs. 18.3% at baseline; McNemar-test, *p =* .*124*). The proportions of individuals living independently remained stable (n = 93, 84.5% vs. 88.7% at baseline; McNemar-test, *p =* .*424*) with no significant differences between SSD and BD. Analyzing the last two years of treatment separately, no significant differences between employment/occupation (n = 32, 27.3% after 4 years vs. 29.1% at 2 years; McNemar-test, *p =* .*804*) and living independently (n = 90, 84.5% after 4 years, 78.3% after 2 years vs.; McNemar-test, *p =* .*375*) were detected.

The CSQ-8 scores indicated a significantly better than baseline satisfaction with care, with a mean rating of “good” at 12-, 24-, and 48-month follow-ups. Satisfaction with treatment improved similarly in both patients with BD and with SSD.

## Discussion

The ACCESS-model was incorporated into clinical routine as a time-unlimited care model and delivered to a sample of severely ill patients, primarily with multiple-episode SSD and BD, and thus very complex treatment needs. The initial sample of 115 patients treated with ACCESS was assessed over 48 months.

The service disengagement rate over 48 months remained very low at 8.7% overall (3.7% within the first 24 months) and was therefore even lower than the ACCESS-I 1-year disengagement rate of 6.3%. Another 23 patients (20.0%) dropped out for practical reasons. Fourteen of these were transferred to more appropriate care (i.e., 10 to sheltered housing and 4 to other services). For the remaining 9 patients who dropped out for practical reasons (insurance, catchment area) appropriate care outside the ACCESS-model was assured. Even if all non-practical and practical dropouts are counted, the resulting rate of 28.7% over 4 years is very low, indicating excellent engagement rates in ACCESS-II (see Tables A and B in S1 File, supporting information). Shorter-term dropouts in SMI’s have been reported at 30% [32], but most of these patients lost contact with the mental health care system. The positive effect of ACT on sustained service engagement may be related to the lower and shared case load, higher contact frequency, no drop-out policy, 24-hour-per-day availability, and the possibility of visiting patients in the community, especially if at risk of disengagement. Other studies with first-episode patients reported disengagement rates of 21.4% in over 24 months [33] or 23.3% over 18 months [10]. In the latter sample, an important predictor of service disengagement was a medium level of illness severity with little improvement during treatment [34]. Therefore, the low disengagement rates in ACCESS, may be in part related to the high illness severity of our sample.

Patients in ACCESS received intensive outpatient treatment with a mean of 2.0–2.4 contacts per week. This rate remained stable over the 4 years. The majority received between 1 and 3 contacts per week, as indicated by the low standard deviation. This continuously high rate of outpatient contact in ACT was also reported by Gold et al. in a retrospective 4-year study [35]. We assume—without specific empirical evidence from our data—that this continuously high weekly contact rate was a key component of ACCESS in stabilizing patients’ psychopathology, functioning, and satisfaction with care and thereby the therapeutic alliance and service engagement. To further strengthen engagement, we regularly involved family members or significant others and applied a recovery-oriented psychotherapeutic approach.

This, together with the high outpatient contact frequency, may also have led to the very low hospitalization rate. It is important to note that 10 patients (8.7%) were transferred to sheltered homes, which otherwise may have increased the hospitalization rate. That is, without a sheltered home option the hospitalization rate might have been higher. OPUS also reported a significantly lower hospitalization rate (mean = 96 days) compared to conventional treatment (123 days) [15]. This difference was still present at the 5-year, but not at the 10-year, follow-up [15, 36]. However, the hospitalization rates in ACCESS were generally lower, which may be due, among other factors, to the fact that ACCESS offered continuous 4-year ACT/Integrated Care.

Another important result of ACCESS is the much lower rate of involuntary admissions (7.8% over 4 years) compared to the 2 years before ACCESS-care (34.8%). There is evidence that the availability of home treatment can reduce the number of involuntary admissions [37, 38]. This is most likely related to fast and flexible access to treatment, particularly in the event of crises, and to the preventive potential of intensive outpatient care.

Overall, patients’ psychopathology, illness severity, functioning, and quality of life improved with medium to large effect sizes. Comparable to the 24-month outcome of ACCESS-II and consistent with the literature, patients with BD had greater improvements in psychopathology and quality of life than patients with SSD.[39–41] Analyzing the last two years separately, only severity of illness continuously improved from year 2 to year 4, while all other outcome measures remained stable. Although comparability is limited by the sample composition and the care model itself, the OPUS-trials reported a deterioration of patients’ psychopathology after OPUS-care was stopped [15, 36], indicating that care should be offered continuously in an open-ended, need-adapted manner. In this study we observed that ongoing active treatment stabilized severely ill patients. Yet, causal effects cannot be proven due to the non-randomized and single group design of the study. That is, it may be more efficient to offer affordable, flexible, but highly specific long-term care as opposed to intensive, potentially expensive short-term care in first-episode and—as our results confirm—multiple-episode psychosis [42].

The present study has a number of strengths. The introduction of the ACCESS-model into clinical routine after comparing the model to standard care in research settings is an important step to transfer research into practice. Furthermore, the clinical settings led to the inclusion of patients who would probably have declined study participation in a (randomized)-controlled trial. The 4-year follow-up time allowed for the assessment of long-term effects of continuous treatment beyond initial improvements and whether these effects can be sustained.

The main inherent limitation is the absence of a control group in the ACCESS II study. Therefore, a direct causal effect of the treatment program on the experienced improvement can´t be drawn. Instead, other factors may also be responsible for improvements or stability of psychopathology in the patients over 4 years. Thus, the data must be interpreted as observational. After the prospective controlled study confirmed superiority of the ACCESS model above treatment as usual over 1 year, only an observational and non-controlled long-term study was considered ethical in our hospital. Another unavoidable limitation was the non-blinded assessment of patients. We used external raters to assure assessment quality and to reduce—but not to fully avoid—social desirability bias and thus too-positive ratings of psychopathology. One main outcome—service-disengagement—was not biased by social desirability or non-blinded assessments. However, the external assessments themselves may have further strengthened service-engagement. As the sample size of the BD group is rather small, analyses of differences between SSD and BD may be underpowered. In addition, the representativeness of the sample may be limited by the exclusion of homeless patients, who were, by definition of the catchment area, treated elsewhere. Newly established teams have better short-term outcomes due to, for example, team enthusiasm and initially smaller caseloads. Due to the long follow-up time, we can rule out these factors. Furthermore, we cannot rule out that important confounders were not assessed, including the specific effect of antipsychotic or mood stabilizer treatments on outcomes.

### Conclusion

To our knowledge, this is the first long-term study confirming the effectiveness of continuous treatment with ACT embedded in an integrated care system in a clinical routine setting for patients with severe and mostly multiple-episode schizophrenia spectrum disorders and bipolar I disorder with psychotic features. While other short-term interventions, including ACT with longer-term follow-ups, reported loss of treatment-related improvements in patients with first-episode psychosis after these model interventions were stopped, the open-end ACCESS-model produced stable outcomes with further improvements in illness severity with low disengagement and hospitalization rates. We assume that the following factors explain these beneficial results. The setting should allow for psychosis-specific ACT embedded in integrated care with direct links to inpatient care and a broad spectrum of treatment options for psychosis and comorbidities. It should be offered in an open-ended, need-adapted manner with a low-enough case-load to enable several outpatient contacts per week. The treatment team should be committed to psychotherapy and family involvement, and should be recovery-oriented.

In summary, the results of the study are consistent with good outcomes, but to draw causal conclusions, stronger evidence including a long-term RCT would be required.

## Supporting information

S1 File Table A time to service-disengagement (non-practical reasons) and table B time to service-disengagement (practical reasons).
(DOCX)

S2 File Study protocol. (DOCX)

S3 File Translated study information. (PDF)

S4 File Trend protocol. (DOCX)

S5 File CT in German. (DOCX)

## References

[1] NICE. Psychosis and Schizophrenia in Adults: NICE Guideline. In: Excellence NIfHaC, editor. London, UK 2014.

[2] Austin SF, Mors O, Secher RG, Hjorthoj CR, Albert N, Bertelsen M, et al Predictors of recovery in first episode psychosis: the OPUS cohort at 10 year follow-up. Schizophr Res. 2013;150(1):163–8. doi: 10.1016/j.schres.2013.07.031 . 23932664 10.1016/j.schres.2013.07.031

[3] Hafner H, Maurer K, An der Heiden W. [Schizophrenia—a disorder in its own right?: results from 25 years of the ABC study]. Nervenarzt. 2013;84(9):1093–4, 6–103. doi: 10.1007/s00115-013-3788-6 . 23695002 10.1007/s00115-013-3788-6

[4] Lambert M, Bock T, Schottle D, Golks D, Meister K, Rietschel L, et al Assertive community treatment as part of integrated care versus standard care: a 12-month trial in patients with first- and multiple-episode schizophrenia spectrum disorders treated with quetiapine immediate release (ACCESS trial). J Clin Psychiatry. 2010;71(10):1313–23. doi: 10.4088/JCP.09m05113yel . 20361911 10.4088/JCP.09m05113yel

[5] Marshall M, Lockwood A. Assertive community treatment for people with severe mental disorders. Cochrane Database Syst Rev. 2000;(2):CD001089 doi: 10.1002/14651858.CD001089 . 10796415 10.1002/14651858.CD001089

[6] Schottle D, Schimmelmann BG, Karow A, Ruppelt F, Sauerbier AL, Bussopulos A, et al Effectiveness of integrated care including therapeutic assertive community treatment in severe schizophrenia spectrum and bipolar I disorders: the 24-month follow-up ACCESS II study. J Clin Psychiatry. 2014;75(12):1371–9. doi: 10.4088/JCP.13m08817 . 25188752 10.4088/JCP.13m08817

[7] Stein LI, Test MA. Alternative to mental hospital treatment. I. Conceptual model, treatment program, and clinical evaluation. Arch Gen Psychiatry. 1980;37(4):392–7. . 7362425 10.1001/archpsyc.1980.01780170034003

[8] Sytema S, Wunderink L, Bloemers W, Roorda L, Wiersma D. Assertive community treatment in the Netherlands: a randomized controlled trial. Acta Psychiatr Scand. 2007;116(2):105–12. doi: 10.1111/j.1600-0447.2007.01021.x . 17650271 10.1111/j.1600-0447.2007.01021.x

[9] Schottle D, Karow A, Schimmelmann BG, Lambert M. Integrated care in patients with schizophrenia: results of trials published between 2011 and 2013 focusing on effectiveness and efficiency. Curr Opin Psychiatry. 2013;26(4):384–408. doi: 10.1097/YCO.0b013e328361ec3b . 23722100 10.1097/YCO.0b013e328361ec3b

[10] Conus P, Lambert M, Cotton S, Bonsack C, McGorry PD, Schimmelmann BG. Rate and predictors of service disengagement in an epidemiological first-episode psychosis cohort. Schizophr Res. 2010;118(1–3):256–63. doi: 10.1016/j.schres.2010.01.032 . 20206475 10.1016/j.schres.2010.01.032

[11] Stowkowy J, Addington D, Liu L, Hollowell B, Addington J. Predictors of disengagement from treatment in an early psychosis program. Schizophr Res. 2012;136(1–3):7–12. Epub 2012/02/18. doi: S0920-9964(12)00052-7 [pii] doi: 10.1016/j.schres.2012.01.027 . 22336955 10.1016/j.schres.2012.01.027

[12] Karow A, Reimer J, Konig HH, Heider D, Bock T, Huber C, et al Cost-effectiveness of 12-month therapeutic assertive community treatment as part of integrated care versus standard care in patients with schizophrenia treated with quetiapine immediate release (ACCESS trial). J Clin Psychiatry. 2012;73(3):e402–8. doi: 10.4088/JCP.11m06875 . 22490266 10.4088/JCP.11m06875

[13] Nordentoft M, Petersen L, Jeppesen P, Thorup AA, Abel MB, Ohlenschlaeger J, et al [OPUS: a randomised multicenter trial of integrated versus standard treatment for patients with a first-episode psychosis—secondary publication]. Ugeskr Laeger. 2006;168(4):381–4. Epub 2006/01/27. doi: VP48017 [pii]. . 16436240

[14] Petersen L, Nordentoft M, Jeppesen P, Ohlenschaeger J, Thorup A, Christensen TO, et al Improving 1-year outcome in first-episode psychosis: OPUS trial. Br J Psychiatry Suppl. 2005;48:s98–103. doi: 10.1192/bjp.187.48.s98 . 16055817 10.1192/bjp.187.48.s98

[15] Bertelsen M, Jeppesen P, Petersen L, Thorup A, Ohlenschlaeger J, le Quach P, et al Five-year follow-up of a randomized multicenter trial of intensive early intervention vs standard treatment for patients with a first episode of psychotic illness: the OPUS trial. Arch Gen Psychiatry. 2008;65(7):762–71. doi: 10.1001/archpsyc.65.7.762 . 18606949 10.1001/archpsyc.65.7.762

[16] First MB, Spitzer RL, Miriam G, Williams JBW. Structured Clinical Interview for DSM-IV-TR Axis I Disorders, Research Version, Patient Edition. (SCID-I/P) New York: Biometrics Research, New York State Psychiatric Institute; 2002.

[17] Overall J, Gorham D. The brief psychiatric rating scale. Psychological Reports. 1962;10:799–812.

[18] Teague GB, Bond GR, Drake RE. Program fidelity in assertive community treatment: development and use of a measure. Am J Orthopsychiatry. 1998;68(2):216–32. . 9589760 10.1037/h0080331

[19] Lambert M, Conus P, Cotton S, Robinson J, McGorry PD, Schimmelmann BG. Prevalence, predictors, and consequences of long-term refusal of antipsychotic treatment in first-episode psychosis. J Clin Psychopharmacol. 2010;30(5):565–72. doi: 10.1097/JCP.0b013e3181f058a0 . 20814327 10.1097/JCP.0b013e3181f058a0

[20] Tohen M, Hennen J, Zarate CM Jr., Baldessarini RJ, Strakowski SM, Stoll AL, et al Two-year syndromal and functional recovery in 219 cases of first-episode major affective disorder with psychotic features. Am J Psychiatry. 2000;157(2):220–8. doi: 10.1176/appi.ajp.157.2.220 . 10671390 10.1176/appi.ajp.157.2.220

[21] McGorry PD, Copolov DL, Singh BS. Royal Park Multidiagnostic Instrument for Psychosis: Part I. Rationale and review. Schizophr Bull. 1990;16(3):501–15. Epub 1990/01/01. . 2287936 10.1093/schbul/16.3.501

[22] McGorry PD, Singh BS, Copolov DL, Kaplan I, Dossetor CR, van Riel RJ. Royal Park Multidiagnostic Instrument for Psychosis: Part II. Development, reliability, and validity. Schizophr Bull. 1990;16(3):517–36. Epub 1990/01/01. . 2287937 10.1093/schbul/16.3.517

[23] Schimmelmann BG, Huber CG, Lambert M, Cotton S, McGorry PD, Conus P. Impact of duration of untreated psychosis on pre-treatment, baseline, and outcome characteristics in an epidemiological first-episode psychosis cohort. J Psychiatr Res. 2008;42(12):982–90. doi: 10.1016/j.jpsychires.2007.12.001 . 18199456 10.1016/j.jpsychires.2007.12.001

[24] Kane JM, Leucht S, Carpenter D, Docherty JP. The expert consensus guideline series. Optimizing pharmacologic treatment of psychotic disorders. Introduction: methods, commentary, and summary. J Clin Psychiatry. 2003;64 Suppl 12:5–19. Epub 2003/12/03. . 14640142

[25] Guy W. Clinical Global Impression. Guy W, editor. Rockville, MD: National Institute for Mental Health; 1976.

[26] American Psychiatric Association DI. American Psychiatric Association and Task Force on DSM IV Diagnostic and Statistical Manual of Mental Disorders, DSM IV. 4 ed. Association AP, editor. Washington, DC: APA; 1994.

[27] Ritsner M, Kurs R, Gibel A, Ratner Y, Endicott J. Validity of an abbreviated quality of life enjoyment and satisfaction questionnaire (Q-LES-Q-18) for schizophrenia, schizoaffective, and mood disorder patients. Qual Life Res. 2005;14(7):1693–703. . 16119181 10.1007/s11136-005-2816-9

[28] Nguyen TD, Attkisson CC, Stegner BL. Assessment of patient satisfaction: development and refinement of a service evaluation questionnaire. Eval Program Plann. 1983;6(3–4):299–313. . 10267258 10.1016/0149-7189(83)90010-1

[29] Jorgensen B. Exponential Dispersion Models. J Roy Stat Soc B Met. 1987;49(2):127–62. PubMed PMID: WOS:A1987J844700001.

[30] Vickers A, Altman D. Analysing controlled trials with baseline and follow up measurements. BMJ. 2001;323:1123–4. 11701584 10.1136/bmj.323.7321.1123 PMC1121605

[31] Cortina J, Nouri H. Effect size for ANOVA designs. Oaks T, editor. Calif: Sage Publications; 2000.

[32] Kreyenbuhl J, Nossel IR, Dixon LB. Disengagement from mental health treatment among individuals with schizophrenia and strategies for facilitating connections to care: a review of the literature. Schizophr Bull. 2009;35(4):696–703. doi: 10.1093/schbul/sbp046 ; PubMed Central PMCID: PMCPMC2696379. 19491314 10.1093/schbul/sbp046 PMC2696379

[33] Petersen L, Jeppesen P, Thorup A, Abel MB, Ohlenschlaeger J, Christensen TO, et al A randomised multicentre trial of integrated versus standard treatment for patients with a first episode of psychotic illness. BMJ. 2005;331(7517):602 doi: 10.1136/bmj.38565.415000.E01 ; PubMed Central PMCID: PMCPMC1215551. 16141449 10.1136/bmj.38565.415000.E01 PMC1215551

[34] Schimmelmann BG, Conus P, Schacht M, McGorry P, Lambert M. Predictors of service disengagement in first-admitted adolescents with psychosis. J Am Acad Child Adolesc Psychiatry. 2006;45(8):990–9. doi: 10.1097/01.chi.0000223015.29530.65 . 16865042 10.1097/01.chi.0000223015.29530.65

[35] Gold PB, Meisler N, Santos AB, Carnemolla MA, Williams OH, Keleher J. Randomized trial of supported employment integrated with assertive community treatment for rural adults with severe mental illness. Schizophr Bull. 2006;32(2):378–95. Epub 2005/09/24. doi: sbi056 [pii] doi: 10.1093/schbul/sbi056 ; PubMed Central PMCID: PMC1435374. 16177278 10.1093/schbul/sbi056 PMC1435374

[36] Secher RG, Hjorthoj CR, Austin SF, Thorup A, Jeppesen P, Mors O, et al Ten-year follow-up of the OPUS specialized early intervention trial for patients with a first episode of psychosis. Schizophr Bull. 2015;41(3):617–26. doi: 10.1093/schbul/sbu155 ; PubMed Central PMCID: PMCPMC4393691. 25381449 10.1093/schbul/sbu155 PMC4393691

[37] Juckel G, Haussleiter I. [Involuntary Admissions in Accordance to the Mental Health Act (PsychKG)—What are the Strongest Predictors?]. Psychiatr Prax. 2015;42(3):133–9. doi: 10.1055/s-0034-1369866 . 24723039 10.1055/s-0034-1369866

[38] McGarvey EL, Leon-Verdin M, Wanchek TN, Bonnie RJ. Decisions to initiate involuntary commitment: the role of intensive community services and other factors. Psychiatr Serv. 2013;64(2):120–6. Epub 2013/03/12. doi: 10.1176/appi.ps.000692012 . 23475404 10.1176/appi.ps.000692012

[39] Benabarre A, Vieta E, Colom F, Martinez-Aran A, Reinares M, Gasto C. Bipolar disorder, schizoaffective disorder and schizophrenia: epidemiologic, clinical and prognostic differences. Eur Psychiatry. 2001;16(3):167–72. Epub 2001/05/17. doi: S0924-9338(01)00559-4 [pii]. . 11353595 10.1016/s0924-9338(01)00559-4

[40] Latalova K, Prasko J, Diveky T, Kamaradova D, Velartova H. Quality of life in patients with bipolar disorder—a comparison with schizophrenic patients and healthy controls. Psychiatr Danub. 2011;23(1):21–6. Epub 2011/03/31. . 21448093

[41] Treuer T, Tohen M. Predicting the course and outcome of bipolar disorder: a review. Eur Psychiatry. 2010;25(6):328–33. Epub 2010/05/07. doi: S0924-9338(10)00053-2 [pii] doi: 10.1016/j.eurpsy.2009.11.012 . 20444581 10.1016/j.eurpsy.2009.11.012

[42] Craig TK, Garety P, Power P, Rahaman N, Colbert S, Fornells-Ambrojo M, et al The Lambeth Early Onset (LEO) Team: randomised controlled trial of the effectiveness of specialised care for early psychosis. BMJ. 2004;329(7474):1067 doi: 10.1136/bmj.38246.594873.7C ; PubMed Central PMCID: PMCPMC526115. 15485934 10.1136/bmj.38246.594873.7C PMC526115

