# Supplementary material for: Effectiveness of integrated care including therapeutic assertive community treatment in severe schizophrenia-spectrum and bipolar I disorders: Four-year follow-up of the ACCESS II study
Source: PLoS One. 2018 Feb 27;13(2):e0192929. doi: 10.1371/journal.pone.0192929 (PMC5828355; doi:10.1371/journal.pone.0192929)
Supplement: S1 File — Table A time to service-disengagement (non-practical reasons) and table B time to service-disengagement (practical reasons). (DOCX) [file pone.0192929.s001.docx]

**Supporting information S1 File**

S1 table 1 time to service-disengagement (non-practical reasons)

| **Time to disengagement in weeks** | **Number of patients** |
| --- | --- |
| 16.0 | 1 |
| 22.6 | 1 |
| 51.1 | 1 |
| 104.4 | 1 |
| 107.8 | 1 |
| 109.1 | 1 |
| 157.0 | 1 |
| 157.6 | 1 |
| 178.4 | 1 |
| 183.9 | 1 |

S1 table 2 time to service-disengagement (practical reasons)

| **Time to disengagement in weeks** | **Number of patients** |
| --- | --- |
| 5.9 | 1 |
| 9.7 | 1 |
| 19.7 | 1 |
| 36.1 | 1 |
| 53.7 | 1 |
| 61.0 | 1 |
| 64.3 | 1 |
| 66.0 | 1 |
| 88.1 | 1 |
| 92.7 | 1 |
| 96.6 | 1 |
| 104.4 | 1 |
| 107.4 | 1 |
| 112.6 | 1 |
| 122.1 | 1 |
| 128.4 | 1 |
| 131.7 | 1 |
| 134.4 | 1 |
| 146.3 | 1 |
| 151.6 | 1 |
| 154.9 | 1 |
| 164.9 | 1 |
| 181.7 | 1 |
