# Supplementary material for: Effectiveness of integrated care including therapeutic assertive community treatment in severe schizophrenia-spectrum and bipolar I disorders: Four-year follow-up of the ACCESS II study
Source: PLoS One. 2018 Feb 27;13(2):e0192929. doi: 10.1371/journal.pone.0192929 (PMC5828355; doi:10.1371/journal.pone.0192929)
Supplement: S2 File — (DOCX) [file pone.0192929.s002.docx]

Universitätsklinikum Hamburg-Eppendorf

Zentrum für Psychosoziale Medizin
Klinik und Poliklinik für Psychiatrie und Psychotherapie und
Klinik für Kinder- und Jugendpsychiatrie und -psychotherapie

Martinistraße 52
20246 Hamburg
Telefon: (040) 7401-57483
Telefax: (040) 7410-55455

PD Dr. med. Anne Karow
karow@uke.uni-hamburg.de
http://www.uke.uni-hamburg.de

An die Ethik-Kommission bei der Ärztekammer Hamburg

GESUNDHEITSNETZ PSYCHOSE: Integrierte Versorgung nach dem Hamburger Modell bei Patienten mit Psychosen – Vergleich des klinischen Verlaufs in der Regelversorgung mit einer historischen Kontrollgruppe

Antrag auf Prüfung einer Studie im Rahmen der medizinischen Forschung im Hinblick auf ethische Unbedenklichkeit

1 Persönliche Angaben

1.1 Antragsteller

- PD Dr. Anne Karow, Klinik und Poliklinik für Psychiatrie und Psychotherapie, Zentrum für Psychosoziale Medizin, Universitätsklinikum Hamburg-Eppendorf, Martinistr. 52, 20246 Hamburg, Tel: 040 / 7410-57483; Fax: 040 / 7410-55455, E-Mail: karow@uke.de
- Dr. Daniel Schöttle, Klinik und Poliklinik für Psychiatrie und Psychotherapie, Zentrum für Psychosoziale Medizin, Universitätsklinikum Hamburg-Eppendorf, Martinistr. 52, 20246 Hamburg, Tel: 040 / 7410-51147; Fax: 040 / 7410-55455, E-Mail: d.schöttle@uke.de

1.2 Klinische und organisatorische Mitarbeiter der Integrierten Versorgung

Prof. Dr. Martin Lambert	Leiter des Arbeitsbereichs Psychosen
Prof. Dr. Dipl. Psych. Thomas Bock	Leiter der Psychosen Ambulanz
Fr. Dr. med. Lia Nika	Mitarbeiter des ACT Teams
Fr. Dr. med. Alexandra Bussopulos	Mitarbeiter des ACT Teams
Dr. Dipl.-Psych. Michael Schödlbauer	Mitarbeiter des ACT Teams
Hr.Dipl.-Psych. Dietmar Golks	Mitarbeiter des ACT Teams
Fr. Dipl. Psych. Andrea Kerstan	Mitarbeiter des ACT Teams
FKS Marietta Frieling	Mitarbeiter des ACT Teams
Fr. Dr. med. Gunda Ohm	Strategische Unternehmensentwicklung
Fr. Dr. Christina Meigel-Schleiff	Kaufmännische Leitung
Hr. Benjamin Lange	Kaufmännische Leitung
Dipl.-Psych. Anne-Lena Sauerbier	Wissenschaftliche Mitarbeiter
Dipl.-Psych. Liz Rietschel	Wissenschaftliche Mitarbeiter

1.3 Leiter des beteiligten Behandlungszentrums

Prof. Dr. med. Dieter Naber
Ärztlicher Direktor der Klinik und Poliklinik für Psychiatrie und Psychotherapie
Martinistrasse 52, D-20246 Hamburg
Telefon: +49−40−7410−52201
Fax: +49−40−7410−52999
E-Mail: naber@uke.de

1.4 Angaben zur wissenschaftlichen Qualifikation

Die Antragsteller führen seit Jahren im Rahmen wissenschaftlicher Projekte Studien zu psychiatrischen Fragestellungen durch, insbesondere auf dem Gebiet der pharmakologischen und nicht-pharmakologischen Therapie psychotischer Erkrankungen. Im Übrigen sei auf Anlagen 2 (Curriculum Vitae der Antragsteller) und 3 (Schriftenverzeichnis) verwiesen.

1.5 Finanzierung der Studie

Die Studie wird durch die Klinik für Psychiatrie und Psychotherapie finanziert.

1.6 Adressat des Gebührenbescheides der Ärztekammer Hamburg

Der Gebührenbescheid der Ärztekammer Hamburg soll an die Projektleiterin, Frau PD Dr. Anne Karow, gerichtet werden (PD Dr. Anne Karow, Klinik und Poliklinik für Psychiatrie und Psychotherapie, Zentrum für Psychosoziale Medizin, Martinistrasse 52, D-20246 Hamburg).

2 Beschreibung und wissenschaftliche Begründung des Projekts

2.1 Erläuterung der Versuchsziele

Im Rahmen der vorliegenden quasi-experimentellen Studie soll die **Wirksamkeit der integrierten Versorgung nach dem Hamburger Modell (IV) als Bestandteil der Regelversorgung im Vergleich zu einer historischen Kontrollgruppe** untersucht werden. Für die Hauptfragestellung sollen Patienten in der IV-Intervention nach dem Hamburger Modell hinsichtlich ihrer psychofunktionalen Remissionsrate im Langzeitverlauf untersucht und mit einer historischen Kontrollgruppe (ACCESS Studie) verglichen werden. Sekundäre Erfolgskriterien umfassen die Verbesserung der Krankheitssymptome, des Funktionsniveaus, der Lebensqualität, die Einhaltung der Medikation, die Zufriedenheit des Patienten mit der Behandlung, die Kosteneffektivität der Behandlung und die Sicherheit der Behandlung (Selbst-/Fremdgefährdung). Bei der historischen Kontrollgruppe handelt es sich um Patienten aus der ACCESS Studie, die die Integrierte Versorgung bei Patienten mit Psychosen unter kontrollierten Bedingungen über 12 Monate untersuchte (Karow et al., in press; Lambert et al., 2010). Die vorliegende Studie erweitert die Ergebnisse dieser Referenzuntersuchung um Daten aus der Regelversorgung („real life“ Bedingungen) und kann somit zu einer nachhaltigen Implementierung der IV in die Regelversorgung in Deutschland beitragen.

Die klinische Auswertung soll anhand von Qualitätssicherungsdaten aus dem Hamburger Modell für Integrierte Versorgung der Klinik für Psychiatrie und Psychotherapie des Universitätsklinikums Hamburg-Eppendorf durchgeführt werden. Die vorliegenden Qualitätssicherungsdaten werden seit Beginn der Integrierten Versorgung nach dem Hamburger Modell für die beteiligten Krankenkassen erhoben.

2.2 Darstellung des bisherigen Wissensstandes

2.2.1 Krankheitsverlauf von Psychosen

Beginnende oder manifeste Psychosen, also psychische Erkrankungen bei denen u.a. Halluzinationen, Wahn und Denkstörungen auftreten, sind hochkomplexe Störungen (siehe Abb. 1) deren Verlauf und Prognose maßgeblich von der Qualität der Behandlung abhängen (Conus et al. 2007, Lambert & Naber 2009). Insgesamt existieren 12 verschiedene diagnostische Entitäten, bei denen das Krankheitsbild von psychotischen Symptomen geprägt ist, v.a. die Schizophreniespektrums-Störungen, die Bipolare I Störung oder die schwere Depression (Lambert & Naber 2009). Die Komplexität und Tragweite dieser Erkrankungen lässt sich anhand folgender Forschungsergebnisse belegen (siehe auch Abb. 1):

Abb. 1: Symptome, somatische und psychische komorbide Störungen, sonstige Probleme und daraus resultierende soziale Konsequenzen bei Patienten mit Psychosen

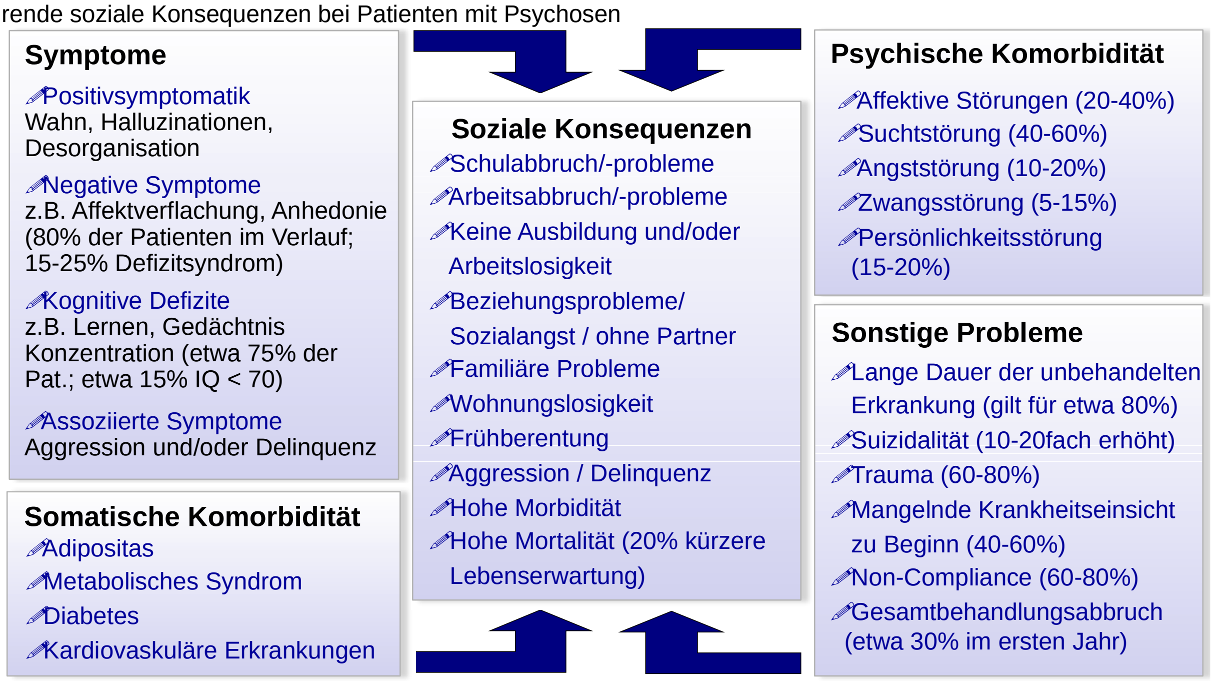

- Psychosen sind relativ häufige Erkrankungen: Nach neusten epidemiologischen Studien allein zur Schizophrenie liegt die Inzidenz bei 15.2 pro 100.000 Einwohner (Varianz 7.7-43.0), die 1-Jahres Prävalenz im Median bei 3.3 pro 100.000 Einwohner, die Lebenszeitprävalenz im Median bei 4.0 pro 100.000 Einwohner und das Lebenszeitmorbiditätsrisiko im Median bei 7.2 pro 100.000 Einwohner (Saha et al. 2005; McGrath & Susser 2009). Für die Bipolar-I-Störung (mit psychotischen Symptomen) liegt die 1- Jahres Prävalenz bei 0.6% und Lebenszeitprävalenz bei 1% (Merikangas et al. 2007).

- Die Erkrankung beginnt meist früh im Leben: Bei den meisten Betroffenen beginnt die Erkrankung zwischen dem 15. und 25. Lebensjahr (Amminger et al. 2006), bis zum 30. Lebensjahr sind 90% der betroffenen Männern und 66% der Frauen erkrankt (Saha et al. 2005). Etwa 20-30% sind bei Erkrankungsbeginn noch im jugendlichen Alter (Schimmelmann et al. 2007).
- Betroffene sind meist lange unbehandelt: Betrachtet man die Dauer der unbehandelten Erkrankung bei psychotischen Störungen sind die Mehrzahl der Betroffenen im Durchschnitt etwa 2 bis 5 Jahre erkrankt bevor eine adäquate biologische und psychosoziale Behandlung beginnt (Marshall et al. 2005; Schimmelmann et al. 2008). Die zunehmende Behandlungsverzögerung hat schwerwiegende Auswirkungen auf den Verlauf und die Prognose der Erkrankung:
    1. zunehmende Häufigkeit von Suizidversuchen vor Beginn der ersten Behandlung (Robinson et al. 2009),
    2. Abnahme des Funktionsniveaus inklusive Arbeitsfähigkeit oder Ausbildungsstatus vor Beginn der ersten Behandlung (Schimmelmann et al. 2008),
    3. Schlechterer Verlauf und Prognose der Erkrankung mit niedrigeren „Recovery“-Raten (symptomatisches plus funktionelles Outcome; Schimmelmann et al. 2008; Lambert et al. 2010), gehäuften Rückfällen (Alvarez-Jiminez et al. 2011) und reduzierter Lebensqualität (Marshall et al. 2005),
    4. Behandlungsunzufriedenheit (Mattson et al. 2005)
    5. schlechterer Therapieteilnahme (Alvarez-Jiminez et al. 2009)
    6. gehäuften Gesamtbehandlungsabbruch (Schimmelmann et al. 2006; Conus et al. 2010a),
    7. Nicht-Remission einer komorbiden Suchterkrankung (Lambert et al. 2005) und erhöhten direkten und indirekten Langzeitkosten.

- Psychosen sind zumeist komplexe Störungen: Diese Komplexität ergibt sich aus der Vielzahl von psychotischen (positiv, negativ, kognitiv, Desorganisation) und assoziierten (Agitation, Depression, Suizidalität) Symptomen, komorbiden psychischen oder somatischen Störungen, Stressoren, Konflikten, Traumata, verzögerten Persönlichkeitsentwicklungen, Funktionseinbußen und

daraus resultierenden sozialen Problemen wie Abbruch von Schule, Ausbildung, Arbeit oder Kontakten zu Familie oder Freunden. Viele der genannten Faktoren (z.B. Suchtkomorbidität oder Traumata) können zu einer schlechten Prognose beitragen, insbesondere wenn sie nicht adäquat diagnostiziert und langfristig behandelt werden. (Lambert & Naber 2009; Conus et al. 2010b).

- Der Verlauf der Erkrankung ist häufig ungünstig: Aufgrund der Komplexität der Erkrankung, der langen Behandlungsverzögerung und eines unzureichenden Hilfesystems (siehe unten) sind die Erkrankungsverläufe psychotischer Störungen häufig ungünstig. Lediglich 10-15% der Betroffenen haben nur eine Episode im Leben, alle anderen haben dauerhafte Symptome nach der ersten Episode oder wiederkehrende Episoden mit oder ohne dauerhafte Symptome bzw. mit einer zunehmenden Verschlechterung ihres Krankheitsbildes (Rosen & Garety 2005).
- Die Behandlung ist häufig schwierig: Zahlreiche Faktoren bedingen gehäuft schwierige Behandlungen bei psychotischen Störungen. Hierzu zählen beispielsweise:
- die hohe Rate von medikamentöser Non-Adhärenz (50% setzen innerhalb eines Jahres, 75% innerhalb 2 Jahre ihre Medikamente ab; Velligan et al. 2009), die hohe Rate von kompletten Medikationsverweigerern (20%; Lambert et al. 2010b), die hohe Rate von Patienten, welche die gesamte Behandlung abbrechen (20-40% innerhalb von 12-18 Monaten; Kreyenbuhl et al. 2009; Lambert et al. 2010c)

2.2.2 Versorgungsspezifische Rationalen für die Entwicklung des Hamburger Modells

Neben erkrankungsspezifischen Rationalen liegen der Konzeption des Hamburger Modells auch versorgungsspezifische Rationalen zugrunde. Neben fehlender bzw. unzureichender Aufklärung und Früherkennung sind diese wie folgt:

- Unzureichende ambulante psychiatrische Behandlung durch Fehlstrukturierung des Versorgungssystems mit Fokus auf vollstationäre Behandlung, Überfinanzierung von ambulanter Psychotherapie im Verhältnis zur Finanzierung von ambulanter psychiatrischer Versorgung, hochschwelligem Zugang zum Hilfesystem, langen Wartezeiten und fehlender Implementierung von intensiven ambulanten Behandlungsmodellen. Als Beispiel sei eine Untersuchung an

902 Psychose-Patienten angeführt, wobei sich ergab, dass etwa 70% der Betroffenen lediglich ein bis drei Termine pro Quartal als Therapie erhielten, die im Durchschnitt 5-30min dauerten. Zudem erhielten nur etwa 8-10% der Patienten, die für sie notwendigen evidenz-basierten Therapien und nur etwa 7% der schwer erkrankten Patienten hatten jemals seit Beginn ihrer Erkrankung eine Psychotherapie erhalten (Lambert et al. 2010).

- Nach neuesten europäischen Studien erhalten nur etwa 8% der Psychose-Betroffenen eine kombinierte medikamentöse und psychosoziale Behandlung (WHO 2011).
- Fehlende Implementierung von intensiven ambulanten Behandlungsmodellen wie Assertive Community Treatment (ACT), trotz hoher Evidenz bei schwer erkrankten Patienten (Lambert et al. 2010c; Marshall & Lockwood 2011).
- In Bezug auf die Kosten betragen die jährlichen direkten Schizophreniekosten in Deutschland etwa 14.000-18.000€ pro Patient (Konnopka et al. 2009). Hinzu kommen Kosten für Angehörige von 950-1.700€ und indirekte Kosten von 25.000-30.000€, so dass die Gesamtkosten bei etwa 39.950-49.700€ pro Jahr pro Patient liegen. Betrachtet man alle Patienten aller Schweregrade zusammen, entstehen 60-70% der direkten Kosten durch (wiederholte) stationäre Behandlungen; mit steigendem Schweregrad steigt dieser Anteil auf bis 90%.

2.2.3 Integrierte Versorgungsmodelle für Psychosen: das Hamburger Modell

Das „Hamburger Modell“ bezeichnet ein Integriertes Versorgungsmodell für schwer erkrankte Psychose Betroffene nach § 140 SGB V, dass eine sektorübergreifende und langfristige Behandlung in einem Netzwerk bestehend aus dem Arbeitsbereich Psychosen des UKE und niedergelassenen Psychiatern beinhaltet (siehe Tabelle 1). Das Modell läuft seit dem 1.5.2007, folgende Krankenkassen nehmen daran teil: DAK, HEK, IKK Classic und AOK Rheinland Hamburg. Für die Teilnahme bestehen krankenkassenspezifische und diagnostische Indikationen sowie Indikationen, die einen hohen Schweregrad der Erkrankung abbilden (siehe 2.2.2.2). Das Behandlungsmodell umfasst als Kernbestandteil sog. Assertive Community Treatment (ACT), darüber hinaus können die Teilnehmer alle Angebote des Arbeitsbereich Psychosen und der beteiligten niedergelassenen Psychiater nutzen (siehe 2.2.2.3). Das Hamburger Modell ist ein sog. „Capitation-Modell“, d.h. das UKE als Hauptvertrags-

nehmer bekommt eine versichertenbezogene Jahrespauschale, mit der alle Leistungen finanziert werden (siehe 2.2.2.4).

Tab. 1: Das Hamburger Modell im Überblick

Information	Details
**Start**	▪ 1.5.2007 (Laufzeit: 4 Jahre, 7 Monate)
**Teilnehmende Krankenkassen**	▪ DAK, HEK, IKK Classic, AOK Rheinland/Hamburg
**Indikation**	▪ Psychotische Störung (12 Diagnosen)<br>▪ Krankenhauseinweisung<br>▪ Hoher allgemeiner Schweregrad der Erkrankung<br>▪ Vorliegen eines assoziierten Syndroms mit hohem Schweregrad
**Finanzierung**	▪ Managed Care Capitation-Modell (Jahrespauschale)<br>▪ Jahrespauschale beginnt am Tag der stationären Aufnahme<br>▪ Für die ersten 2 Jahre Jahrespauschale, dann gestufte Pauschalen je nach Schweregrad der Erkrankung (CGI-S) & Funktionsniveau (GAF) **= Remission** über die letzten 6 Monate vor 2 Jahresfrist<br>▪ Jedes Jahr neue Einstufung in Bezug auf Remission

2.2.4 Indikationen zur Teilnahme am Hamburger Modell

a) Versichert bei einer der folgenden Krankenkassen:

DAK, HEK, IKK Classic und AOK Rheinland Hamburg

b) Erfüllung einer der folgenden Hauptdiagnosen nach ICD-10:

F1-Störungen:	Substanzinduzierte psychotische Störung (F1x.5)
F2-Störungen:	Schizophrenie (F20)<br>Anhaltende wahnhafte Störung (F22)<br>Akute vorübergehende psychotische Störung (F23)<br>Induzierte wahnhafte Störung (F24)<br>Schizoaffektive Störung (F25)<br>Sonstige nichtorganische psychotische Störung (F28)<br>Nicht näher bezeichnende nichtorganische Psychose (F29)
F3-Störungen:	Manische Episode mit psychotischen Symptomen (F30)<br>Bipolar affektive Störung (F31)<br>Schwere depressive Episode mit psychotischen Symptomen (F32.3)<br>Rezidivierende depressive Störung, gegenwärtig schwere depressive Episode mit psychotischen Symptomen (F33.3)

c) Erfüllung mindestens eines der folgenden Schweregrad-Kriterien:

Vorliegende Einweisung:	Einweisung zur stationären Aufnahme von einem niedergelassenen Psychiater oder bei Notfallaufnahme von einem Krankenhausarzt liegt vor. Die Schwere der Erkrankung macht normalerweise eine stationäre Behandlung notwendig.
Erfüllung des allgemeinen Schweregrad-kriteriums:	Der Patient erfüllt einen allgemeinen Schweregrad von einem Gesamtwert von mindestens 40 Punkten in der Brief Psychiatric Rating Scale (BPRS) Chronizitätskriterium hinzu (hatte in den letzten 2 Jahren viele (teil-)stationäre Aufenthalte)
Erfüllung mindestens eines der spezifischen Schweregrad-kriterien:	Halluzinationen (item 10) ≥ 6 Punkte<br>Wahn (item 11) ≥ 6 Punkte<br>Desorganisation (item 15) ≥ 6 Punkte<br>Depressiv-suizidales Syndrom ≥ 10 Punkte<br>Suizidales Syndrom ≥ 6 Punkte<br>Manisches Syndrom ≥ 15 Punkte<br>Verhaltensstörungs-Syndrom im Rahmen einer Psychose ≥ 15 Punkte<br>Syndrom vorherrschender Negativsymptomatik ≥ 15 Punkte

2.2.5 Das Behandlungsmodell

Das Behandlungsmodell umfasst eine sektorübergreifende und langfristige Behandlung in einem Netzwerk bestehend aus dem Arbeitsbereich Psychosen des UKE und niedergelassenen Psychiatern. In Abbildung 2 sind alle beteiligten Institutionen sowie die derzeit 20 beteiligten niedergelassenen Psychiater dargestellt.

Abb. 2: Das Integrierte Versorgungsmodell für Psychosen: Hamburger Modell

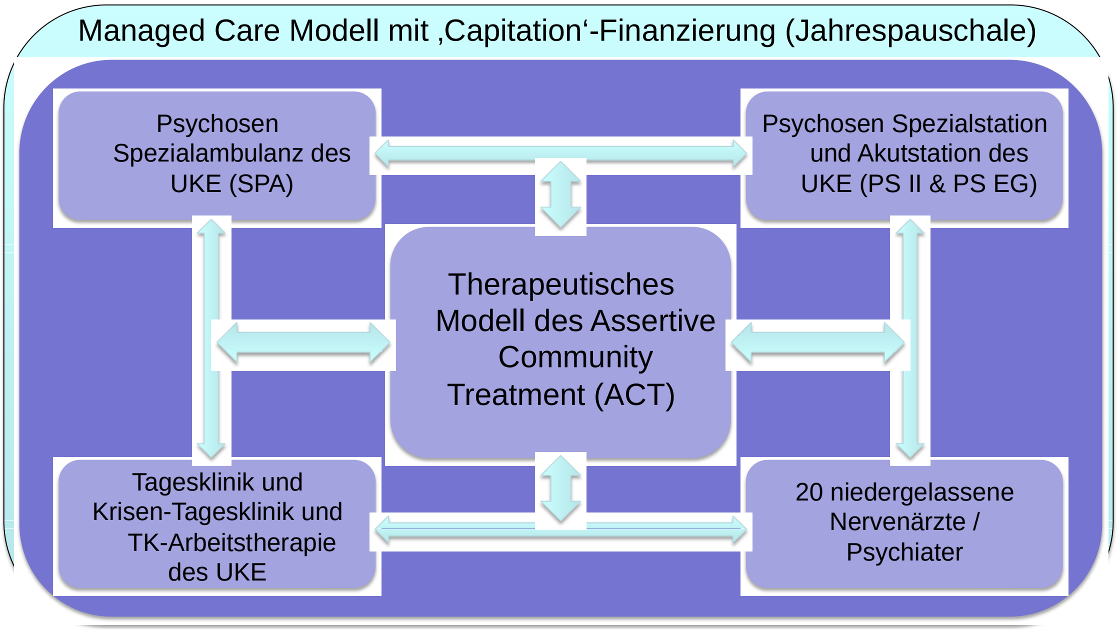

Der Arbeitsbereich Psychosen des UKE (siehe Abbildung 3) umfasst verschiedene Teilbereiche, die alle an der Versorgung psychotischer Patienten beteiligt sind bzw. den Betroffenen und ihren Familien im Rahmen der Integrierten Versorgung zur Verfügung stehen. Die beteiligten Versorgungseinheiten umfassen:

- Die Psychosen Spezialambulanz (SPA) des UKE mit einem langfristigem ambulanten Einzel- und Gruppenangebot sowie ein Spezialangebot für Bipolare Patienten (BiPo Projekt),
- die Krisentagesklinik für Jungerwachsene Psychose Betroffene (altersübergreifend 16-29 Jahre, 8-10 Plätze),
- das Assertive Community Treatment (ACT) Team,
- die Psychosen Spezialstation der Erwachsenpsychiatrie (PS 2, 23 Betten),
- die Akutstation der Erwachsenpsychiatrie (PS EG, 23 Betten),
- 20 niedergelassene Psychiater aus dem erweiterten Sektor (v.a. Eimsbüttel)

Abb. 3: Der Arbeitsbereich Psychosen des UKE

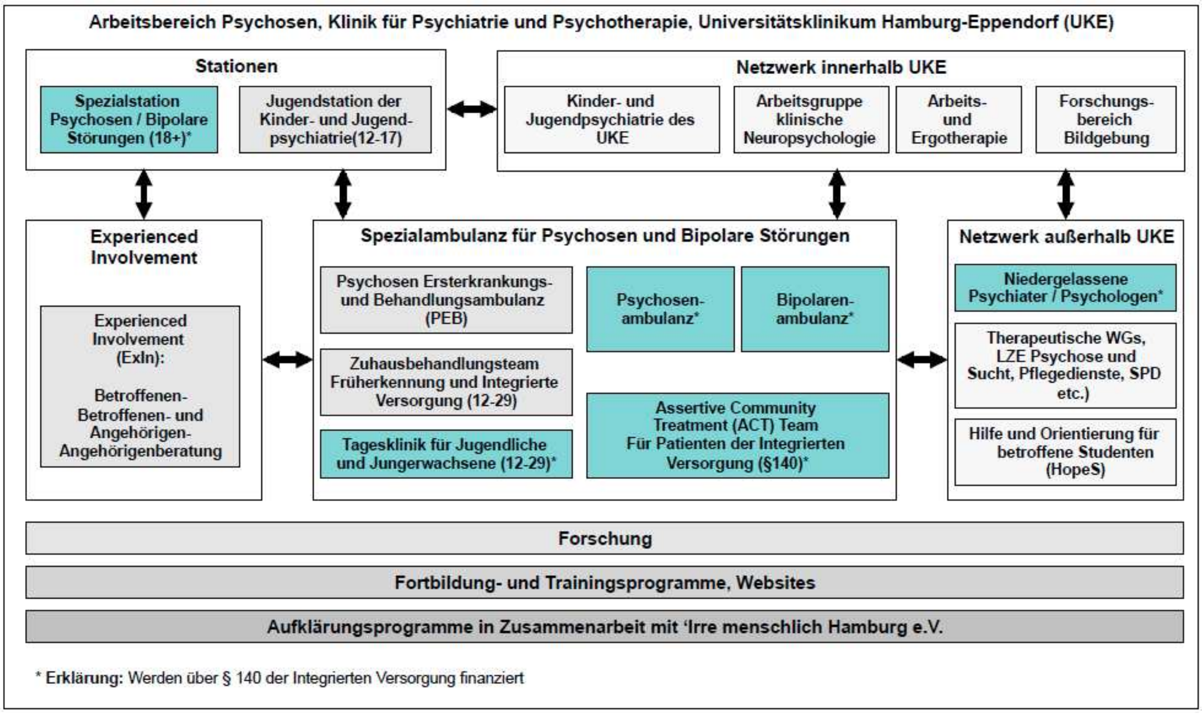

Kernbestandteil der integrierten Behandlung ist sog. Assertive Community Treatment (ACT). ACT ist eines der „evidenzbasierten“ Behandlungsmodelle für schwererkrankte Patienten (engl. „severe mental illness“, SMI) und umfasst eine „aufsuchende, intensive und langfristige Behandlung für Menschen mit chronischen und schweren psychischen Erkrankungen“ (Marshall & Lockwood 2011). Der Aufbau eines ACT Teams wird nach den Richtlinien der Assertive Community Treatment Association (ACTA) durchgeführt. Die Qualität des Teams wird anhand von 28 Kriterien mit der Dartmouth Assertive Community Treatment Scale (DACTS) sichergestellt (Range

von 1 = „poor fidelity“ bis 5 = „excellent fidelity“; Teague et al. 1998). Zu den allgemeinen Qualitätskriterien eines ACT Teams gehören:

- multiprofessionelles Team,
- niedrige Behandler-Patienten-Ratio von 1:10 bis maximal 1:20,
- „no drop out policy“,
- 24h tägliche Erreichbarkeit,
- Einbindung von ACT in zusätzliche Behandlungsoptionen und damit Zugang zu allen „evidenzbasierten“ Therapien und Therapieprogrammen sowie notwendige sozialen Maßnahmen und
- settingübergreifende Behandlungskontinuität.

Bei der ACT Konzeption im Hamburger Modell wurde „traditionelles ACT“ hin zu „therapeutischem ACT“ modifiziert (siehe Abbildung 4 und Tabelle 2).

Tab. 2: Unterschiede zwischen traditionellem ACT und therapeutischem ACT

**Struktur**	**Traditionelles ACT**	**Therapeutisches ACT**
**Indikation**	‚Severe mental illness‘ (SMI)	Psychose
**Team**	Nicht-spezialisiertes Team	Psychose-Experten-Team
**Behandler / Patient-Ratio**	1 : 15/20	1 : 15/20
**Bereitschaft**	24h/tgl. / 365 Tage	24h/tgl. / 365 Tage
**No-drop-out policy**	ja	ja
**Pharmakotherapie**	Häufig keine Verantwortung oder nicht speziell ausgebildet	Experten
**Psychotherapie**	Nicht speziell ausgebildet	Experten
**Aufgaben**	Case Management<br>Home Treatment	Case Management<br>Home Treatment<br>Psychotherapie

Wie in Tabelle 2 dargestellt, existieren wesentliche Unterschiede zwischen traditionellen und therapeutischen ACT. Diese umfassen:

- das ACT Team ist auf eine Erkrankung spezialisiert,
- das ACT Team ist multiprofessionell besetzt und besteht aus Psychose Experten
- das Team hat Expertise für die Pharmakotherapie psychotischer Störungen
- das Team ist speziell ausgebildet für Psychotherapie von Psychosen

die Aufgaben umfassen auch andere evidenzbasierte Interventionen für schwer erkrankte Psychose-Betroffene, u.a. Home Treatment und intensives Case Management. Mit dieser Spezialisierung wird versucht, ein Maximum an störungsspezifischer Behandlungsqualität zu implementieren.

Abb. 4: Therapeutisches ACT als Kernbestandteil des Hamburger Modells

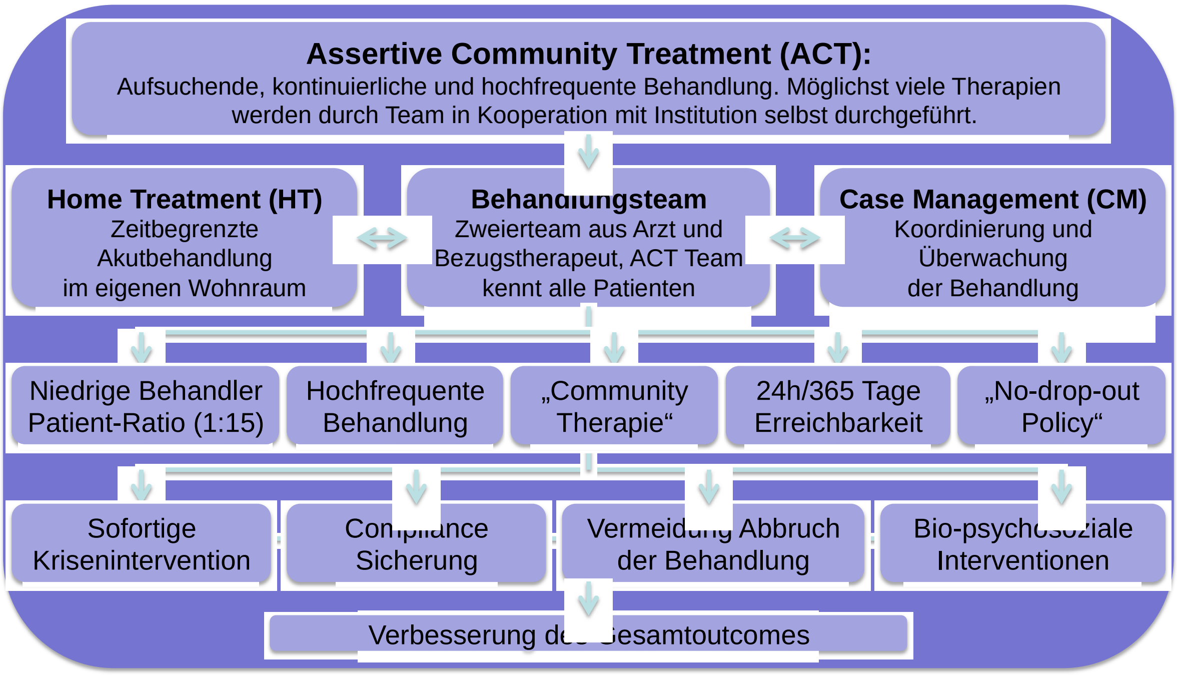

Das Team am UKE ist multiprofessionell besetzt und besteht aus Fachärzten und Assistenzärzten, Psychologen und Fachkrankenpflegepersonal. Es ist einerseits integriert in die Psychosen Ambulanz, anderseits ist es vernetzt mit dem stationären Bereich und allen an der Integrierten Versorgung teilnehmenden niedergelassenen Psychiatern. Im Rahmen der Integrierten Versorgung wird jeder Patient durch ein Zweierteam bestehend aus einem Bezugstherapeuten aus dem ACT Team und einem Arzt (aus dem ACT Team oder niedergelassen), kontinuierlich über die gesamte langfristige Laufzeit behandelt. Das Team trägt die Verantwortung für die strukturelle und inhaltliche Koordination der Therapie. Die Therapieplanung erfolgt mit dem Patienten und wenn immer möglich auch zusammen mit den Angehörigen. Der Patient kann alle im Psychosenbereich angeboten Therapien nutzen. Das sichert den Zugang zu allen „evidenzbasierten“ Gruppentherapien und Therapieprogrammen sowie notwendigen sozialen Maßnahmen. Die niedrige Behandler/Patient-Ratio erlaubt darüber hinaus einen möglichst frühzeitigen Zugang zu Psychotherapie, die durch den jeweiligen ACT Bezugstherapeuten durchgeführt werden kann. Beteiligte niedergelassene Nervenärzte (derzeit 20) haben sich im Rahmen des Vertrages dazu verpflichtet eingeschlossene Patienten ohne Wartezeit, intensiver und in enger Abstimmung mit dem jeweiligen Bezugstherapeuten zu behandeln. Das ACT Team hat damit im Wesentlichen folgende Aufgaben (siehe Abbildung 4):

- Hochfrequente, langfristige und settingübergeifende Behandlung im eigenen Umfeld inklusive Akut- und Langzeitbehandlung und inklusive einer hochqualitativen Pharmakotherapie,
- Verhinderung von Rückfällen durch Krisenintervention 7 Tage die Woche und 24h täglich, sofortige Rückfallbehandlung, sofortige Compliance-fördernde Maßnahmen im Falle von Non-Compliance und sofortige „Re-engagement" bei Gesamtbehandlungsabbruch
- poststationäre intensive Nachsorge und damit Reduktion der Krankenhausverweildauer und
- Planung und Koordination aller Interventionen und damit Zugang zu allen „evidenzbasierten" Gruppentherapien und Therapieprogrammen sowie notwendigen sozialen Maßnahmen
- Möglichst frühzeitiger Zugang zu Psychotherapie, die durch den ACT Mitarbeiter durchgeführt wird.
- Einbindung der niedergelassenen Psychiater

Mittlerweile haben 20 niedergelassene Psychiater ihren Beitritt zum „Hamburger Modell" erklärt. Für Patienten der Integrierten Versorgung haben sie sich dazu verpflichtet, möglichst keine oder nur sehr kurze Wartezeiten vorzuhalten und diese Patienten intensiver als bisher zu betreuen. Zusätzlich zu einer leitliniengerechten Behandlung der psychotischen Störung inklusive der Pharmakotherapie werden weitere Leistungen zur Erfüllung der Vertragsziele erbracht. Dazu gehören:

- Information und Aufklärung des Patienten über die neue Versorgungsform
- kurzfristige Terminvergabe für Patienten der Integrierten Versorgung
- Koordination des individuellen Behandlungssettings mit den anderen Leistungserbringern der Integrierten Versorgung,
- zusätzliche Dokumentation im Rahmen der Integrierten Versorgung zur begleitenden Qualitätssicherung und
- regelmäßige Teilnahme an den intersektoralen Fallkonferenzen inklusive der Vorbereitung der Kasuistiken.

2.2.6 Finanzierung des Hamburger Modells

Das UKE erhält für die psychiatrische Behandlung des Patienten eine Jahrespauschale von den Kostenträgern. Grundlage für die Finanzierung der ersten beiden Behandlungsjahre sind die IST-Kosten der jeweiligen Krankenkasse für die bisherige Krankenhausbehandlung dieses Versichertenkollektives. Dies umfasst die Kosten der stationären und ambulanten Behandlung sowohl im UKE, als auch in anderen Krankenhäusern in Hamburg, deren Anteil rund 20% ausmacht. Aus diesen Kosten wurde der durchschnittliche Wert ermittelt, der die Verhandlungsgrundlage für die zu vereinbarende Jahrespauschale war. Durch die krankenkassenindividuelle Berech-

nung der Jahrespauschale sollte die Morbidität entsprechend der Versichertenstruktur des jeweiligen Kostenträgers abgebildet werden.

Für die Finanzierung der weiteren Behandlung über die 2 Jahre hinaus wurde ein anderer Weg eingeschlagen, der für alle Krankenkassen gleichermaßen gilt: Die Abrechnung erfolgt ab dem 3. Behandlungsjahr über eine von vier möglichen Pauschalen, die patientenindividuell anhand des Schweregrades und des Funktionsniveaus ermittelt wird, unter der Annahme, dass ein schwerer erkrankter Patient einen höheren Behandlungsbedarf hat, als ein gut stabilisierter Patient mit hohem Funktionsniveau. Die Krankenkassen zahlen ab dem 3. Behandlungsjahr insgesamt, über alle Patienten gesehen, deutlich weniger als in den ersten beiden Behandlungsjahren. Damit profitieren finanziell auch die Krankenkassen von der langfristigen Stabilisierung des Patienten.

2.3 Ergebnisse von Vorprüfungen/Unbedenklichkeit

a) Ergebnisse der pharmakologisch-toxikologischen Vorprüfungen bei Arzneimittelstudien [§ 40 (1) 5. und 6. AMG]
   Entfällt

b) Nachweise über die sicherheitstechnische Unbedenklichkeit des Medizinproduktes sowie Ergebnisse der biologischen Sicherheitsprüfung [§ 20 (1) 6. und 7. MPG]
   Entfällt

c) Handelt es sich um eine Untersuchung, auf die §§ 23/24 StrlSchV vom 20.07.2001 Anwendung finden? Werden studienbedingt röntgenologische und/oder nuklearmedizinische Untersuchungen/Behandlungen durchgeführt?
   Entfällt

2.4 Begründung der Notwendigkeit von Humanversuchen

a) Experimente an gesunden Personen?
   Entfällt

b) Heilversuche an Patienten?

Die große medizinische und gesundheitspolitische Bedeutung der Anwendung von IV mit ACT bei der Behandlung von psychotischen Erkrankungen macht ihre Erforschung dringend notwendig. Bisherige Arbeiten sind nicht geeignet, die Fragestellungen der vorliegenden Analyse zu beantworten und unterliegen in ihrer Aussagekraft insbesondere hinsichtlich der Beurteilung des Behandlungserfolges unter „real life“ Bedingungen, d.h. der Beurteilung der integrierten Versorgung als Bestandteil der Regelversorgung, methodischen Einschränkungen. Antworten auf diese Fragestellungen lassen sich nur durch patientenorientierte Forschung gewinnen und machen daher weiterführende Untersuchungen an Patienten notwendig.

2.5 Schilderung der geplanten Versuchsdurchführung

Die vorliegende Studie wird durch das Studienzentrum des Zentrums für Psychosen des Universitätsklinik Hamburg-Eppendorf durchgeführt. Die Behandlungsdauer ist unbegrenzt und entspricht für die gesamte Stichprobe einer Behandlung in IV & ACT. Assertive Community Treatment (ACT) ist eine bislang in Deutschland nur am UKE implementierte Interventionsform. ACT bedeutet, dass eine akute und auch langfristige Behandlung von einem Team von Psychoseexperten im häuslichen Umfeld erfolgt. Das Team am UKE ist multiprofessionell besetzt und besteht aus Fachärzten und Assistenzärzten, Psychologen und Fachkrankenpflegepersonal. Es ist einerseits integriert in die Psychosen Ambulanz, anderseits ist es vernetzt mit dem stationären Bereich und allen an der Integrierten Versorgung teilnehmenden niedergelassenen Psychiatern. Das Team trägt die Verantwortung für die strukturelle und inhaltliche Koordination der Therapie. Die Therapieplanung erfolgt mit dem Patienten und wenn immer möglich auch zusammen mit den Angehörigen. Der Patient kann alle im Psychosenbereich angeboten Therapien nutzen. Das sichert den Zugang zu allen „evidenzbasierten“ Gruppentherapien und Therapieprogrammen sowie notwendigen sozialen Maßnahmen. Die niedrige Behandler/Patient-Ratio erlaubt darüber hinaus einen möglichst frühzeitigen Zugang zu Psychotherapie, die durch den jeweiligen ACT Bezugstherapeuten durchgeführt werden kann. Beteiligte niedergelassene Nervenärzte (derzeit 20) haben sich im Rahmen des Vertrages dazu verpflichtet eingeschlossene Patienten ohne Wartezeit, intensiver und in enger Abstimmung mit dem

jeweiligen Bezugstherapeuten zu behandeln (zur ausführlichen Beschreibung der Behandlungsrationale vgl. 2.2.5).

Alle Patienten in der integrierten Versorgung werden zu den in Tabelle 1 dargestellten Messzeitpunkten von einer geschulten Psychologin untersucht.

Die Kontrollbedingung ist eine historische Kontrollgruppe, die in dem Zeitraum vor Implementierung der Integrierten Versorgung nach dem Hamburger Modell in das Versorgungssystem am UKE bzw. in Rissen im Rahmen der ACCESS Studie („Assertive Community Treatment as part of Integrated Care vs. Standard Care in patients with Schizophrenia„ EudraCT number: 2005-001069-32; Clinicaltrials.gov number: NCT01081418; Nummer Ethik Kommission Hamburg: 2515) behandelt worden ist. Die Kontrollgruppe umfasst 120 Patienten mit Psychosen, die zwischen 1/2006 und 12/2007 an der ACCESS Studie teilgenommen hatten und eine Einverständniserklärung für die Verwendung ihrer Daten unterschrieben hatten. Die ACCESS Studie diente als Referenzstudie für die Einführung der IV nach dem Hamburger Modell.

Tabelle 1. Überblick über die im Laufe der Behandlung durchgeführten Untersuchungen

Untersuchungsparameter	Skalen und Erklärung	Evaluation<br>**Zeitpunkt(e) in Wochen (A = Aufnahme)**
Basisdaten		
**Demographie und klinische Variablen**	**Early Psychosis File Questionnaire (EPFQ; Lambert et al. 2005)**	**A**
**Diagnostische Variablen**	**Hauptdiagnose, Komorbidität (bei Aufnahme und im Verlauf), Suizidversuchsdiagnosen**	**A**
Compliance		
Non-Compliance (objektiv)	**≥ 1 Woche ohne Medikation (Robinson et al. 2005)**	**A, Woche 6, Monat 3 Monat 6, alle 6 Monate, fortlaufend**
Partielle Compliance (objektiv)	**Verpasst 20-80% der Medikation (Kane et al. 2003)**	**A, Woche 6, Monat 3 Monat 6, alle 6 Monate, fortlaufend**
Gesamtbehandlungsabbruch und Behandlungseinbindung		
Gesamtbehandlungsabbruch	**Gesamtbehandlungsabbruch ist evident, wenn der Patient wiederholt trotz “no drop out policy” die weitere Behandlung ablehnt oder trotz vielfacher Versuche nicht mehr kontaktierbar ist.**	**A, Woche 6, Monat 3 Monat 6, alle 6 Monate, fortlaufend**
Behandlungseinbindung	Service Engagement Scale (SES; Tait et al. 2005; Werte von 0-3 Punkten; Durchschnittswert über 14 Fragen; niedrigere Werte = besseres Engagement)	**A, Woche 6, Monat 3 Monat 6, alle 6 Monate, fortlaufend**
Psychopathologie und Schwere der Erkrankung		
BPRS	**Brief Psychiatric Rating Scale (BPRS; Overall & Gorham, 1962; Werte von 0-144 Punkte; niedrigere Werte = bessere Psychopathologie)**	**A, Woche 6, Monat 3 Monat 6, alle 6 Monate, fortlaufend**
CGI-S	**Clinical Global Impression Scale (Guy et al. 1976; Werte von 1-7 Punkten; niedrigere Werte = niedrigere Schwere der Erkrankung)**	**A, Woche 6, Monat 3 Monat 6, alle 6 Monate, fortlaufend**
CGI-BP	**Clinical Global Impression Scale – Bipolar Disorder (CGI-BP; Spearing et al. 1997; Werte von 1-7 Punkten; niedrigere Werte = niedrigere Schwere der Erkrankung; 2 Subskalen für depressive und manische Symptome)**	**A, Woche 6, Monat 3 Monat 6, alle 6 Monate, fortlaufend**
Lebensqualität		
Q-LES-Q-18	**Quality of Life Enjoyment and Satisfaction Questionnaire (Ritsner et al. 2005; 18 Fragen; Werte von 0-5; Durchschnittswert über 18 Fragen; höhere Werte = besseres Lebensqualität)**	**A, Woche 6, Monat 3 Monat 6, alle 6 Monate, fortlaufend**
EQ-5D	**Euro-QoL 5D (Prieto et al. 2004; Werte von 0-5 Punkten; niedrigere Werte = niedrigere Lebensqualität)**	**A, Woche 6, Monat 3 Monat 6, alle 6 Monate, fortlaufend**
Behandlungszufriedenheit		
CSQ-8 Patient	Client Satisfaction Questionnaire (CSQ-8; Larsen et al. 1979; Werte von 8 bis 32, höhere Werte = höhere Behandlungszufriedenheit)	**A, Woche 6, Monat 3 Monat 6, alle 6 Monate, fortlaufend**
CSQ-8 Familie/Angehörige	Client Satisfaction Questionnaire (CSQ-8; Larsen et al. 1979; Werte von 8 bis 32, höhere Werte = höhere Behandlungszufriedenheit)	**A, Woche 6, Monat 3 Monat 6, alle 6 Monate, fortlaufend**
Daten zur Intervention		
Dokumentation	**Ständige Dokumentation aller Leistungen (inkl. Anzahl, Art, Dauer; Bericht alle 3-6 Monate; Anzahl, Dauer, Art der Kontakte/Therapien**	**Ständige Dokumentation**
Kosten		
Dokumentation	Krankenhauskosten: (voll- und teilstationär, Institutsambulanz), Ambulante Kosten, (ACT Team, niedergelassener Psychiater)	**Ständige Dokumentation**

EPFQ: Early Psychosis File Questionnaire (Conus et al., 2007); CGI-S: Clinical Global Impression Scale-Schizophrenia (Guy, 1976); GAF: Global Assessment of Functioning Scale (American Psychiatric Association, 2003); MLVI: Modified Location and Vocation Index (Tohen et al., 2000); Q-LES-Q-18: Quality of Life Enjoyment and Satisfaction Questionnaire (Ritsner, Kurs, Gibel, Ratner, & Endicott, 2005); CSQ-8: Client Satisfaction of Care Questionnaire (Nguyen, Attkisson, & Stegner, 1983); EQ-5D: Euro-Qol-5D (Prieto, Novick, Sacristán, Edgell, & Alonso, 2003);

2.6 Literatur

Alvarez-Jiménez M, Gleeson JF, Cotton S, Wade D, Gee D, Pearce T, Crisp K, Spiliotacopoulos D, Newman B, McGorry PD. Predictors of adherence to cognitive-behavioural therapy in first-episode psychosis. Can J Psychiatry 2009; 54(10): 710-8.

Alvarez-Jimenez M, Gleeson JF, Henry LP, Harrigan SM, Harris MG, Amminger GP, Killackey E, Yung AR, Herrman H, Jackson HJ, McGorry PD. Prediction of a single psychotic episode: a 7.5-year, prospective study in first-episode psychosis. Schizophr Res, 2011; 125(2-3): 236-46.

American Psychiatric Association, American Psychiatric Association, & Task Force on DSM-IV. Diagnostic and statistical manual of mental disorders, DSM-IV. (4th ed ed.) Washington, DC: American Psychiatric Association, 1994.

Amminger GP, Harris MG, Conus P, Lambert M, Elkins KS, Yuen HP, McGorry PD. Treated incidence of first-episode psychosis in the catchment area of EPPIC between 1997 and 2000. Acta Psychiatrica Scand 2006; 14: 337-345.

Conus P, Cotton S, Schimmelmann B, McGorry PD, Lambert M. The First Episode Psychosis Outcome Study (FEPOS): Pre-morbid and baseline characteristics of 786 first episode psychosis patients treated in EPPIC between 1998 and 2000. Early Intervention in Psychiatry 2007; 1: 191-200.

Conus P, Lambert M, Cotton S, McGorry P, Schimmelmann B. Rate and predictors of service disengagement in an epidemiological first-episode psychosis cohort. Schizophr Res 2010a; 118: 256-263.

Conus P, Cotton S, Schimmelmann B, Berk M, Rothanthi D, McGorry P, Lambert M. Pre-treatment and outcome correlates of past sexual and physical trauma in 118 bipolar I patients with a first episode of psychotic mania. Bipolar Disord 2010b; 12: 244-52.

Guy W. ECDEU Assessment Manual for Psychopharmacology, revised. US Dept Health, Education, and Welfare publication (ADM) 76-338. Rockville, Md: National Institute of Mental Health; 1976: 218-222.

Karow A, Reimer J, Schulz H, Bock T, Schöttle D, Golks D, Meister K, Rietschel L, Ohm G, Schimmelmann BG, Huber C, Sadre Chirazi-Stark M, Naber D, Schimmelmann B, Lambert M. Cost-utility analysis of 12 months Assertive Community Treatment as part of Integrated Care versus Standard Care in patients with schizophrenia treated with Quetiapine (ACCESS Trial). J Clin Psychiatry, in press.

Konnopka A, Klingberg S, Wittorf A, König HH. The cost of schizophrenia in Germany: a systematic review of the literature. Psychiatr Prax 2009; 36(5): 211-218.

Kreyenbuhl J, Nossel IR, Dixon LB. Disengagement from mental health treatment among individuals with schizophrenia and strategies for facilitating connections to care: a review of the literature. Schizophr Bull 2009; 35: 696-703.

Lambert M, Conus P, Lubman DI, Wade D, Yuen HP, Moritz S, Naber D, McGorry PD, Schimmelmann B. The impact of substance use disorders on clinical outcome in 668 patients with first-episode psychosis. Acta Psychiatr Scand 2005; 112: 141-148.

Lambert M (Edit.), Naber D. Current Schizophrenia – 2nd edition. Current Medicine Group, 2009, pp. 1-171.

Lambert M, Karow A, Leucht S, Schimmelmann B, Naber D. Remission in schizophrenia: its validity, frequency, predictors and patients' perspective 5 years after. Dialogues Clin Neurosci 2010a; 12: 393-407.

Lambert M, Conus P, Cotton S, Robinson J, McGorry PD, Schimmelmann BG. Prevalence, predictors, and consequences of long-term refusal of antipsychotic treatment in first-episode psychosis. J Clin Psychopharmacol 2010b; 30: 565-72.

Lambert M, Bock T, Schöttle D, Golks D, Meister K, Rietschel L, Bussopulos A, Frieling M, Schödlbauer M, Burlon M, Huber C, Pakrasi M, Ohm G, Sadre Chirazi-Stark M, Naber D, Schimmelmann B. Assertive Community Treatment (ACT) as part of Integrated Care versus Standard Care: a 12-month trial in patients with first- and negatively selected multiple-episode schizophrenia-spectrum disorders treated with quetiapine IR. J Clin Psychiatry 2010c; 71: 1313-23.

Marshall M, Lewis S, Lockwood A, Drake R, Jones P, Croudace T. Association between duration of untreated psychosis and outcome in cohorts of first-episode patients: a systematic review. Arch Gen Psychiatry 2005; 62: 975-83.

Marshall M, Lockwood A. (2011) Assertive community treatment for people with severe mental disorders. Cochrane Database Syst Rev, 13;(4), CD001089.

Mattsson M, Lawoko S, Cullberg J, Olsson U, Hansson L, Forsell Y. Background factors as determinants of satisfaction with care among first-episode psychosis patients. Soc Psychiatry Psychiatr Epidemiol; 2005; 40(9): 749-54.

McGrath JJ, Susser ES. New directions in the epidemiology of schizophrenia. Med J Aust. 2009; 190(4 Suppl):7-9.

Merikangas KR, Akiskal HS, Angst J, Greenberg PE, Hirschfeld RM, Petukhova M, Kessler RC. Lifetime and 12-month prevalence of bipolar spectrum disorder in the National Comorbidity Survey replication. Arch Gen Psychiatry 2007; 64(5):543-52.

Robinson J, Cotton S, Conus P, Schimmelmann B, McGorry PD, Lambert M. Prevalence and predictors of suicide attempt in an incidence cohort of 661 young people with first-episode psychosis. Aust N Z J Psychiatry 2009; 43: 149-57.

Rosen K, Garety P. Predicting recovery from schizophrenia: a retrospective comparison of characteristics at onset of people with single and multiple episodes. Schizophr Bull 2005; 31: 735-50.

Saha S, Chant D, Welham J, McGrath J. A systematic review of the prevalence of schizophrenia. PLoS Med; 2005; 2(5):e141.

Schimmelmann B, Conus P, Schacht M, McGorry PD, Lambert M. Predictors of service disengagement with treatment in first-admitted adolescents with psychosis. J Am Acad Child Adolesc Psychiatry 2006; 45: 990-999.

Schimmelmann B, Conus P, Cotton S, McGorry P, Lambert M. Pre-treatment, baseline, and outcome differences between adolescent-onset and adult-onset psychosis in an epidemiological cohort of 636 first-episode patients. Schizophr Res 2007; 95: 1-9.

Schimmelmann B, Conus P, Cotton S, McGorry PD, Lambert M. Impact of Duration of Untreated Psychosis on Initial Presentation and Outcome in an Epidemiological First Episode Psychosis Cohort. J Psychiatr Res 2008; 42: 982-990.

Spearing MK, Post RM, Leverich GS, et al. Modification of the Clinical Global Impressions (CGI) scale for use in bipolar illness (BP): the CGI-BP. Psychiat Res 1997; 73: 159-171.
Teague GB, Bond GR, Drake RE. Program fidelity in assertive community treatment: development and use of a measure. Am J Orthopsychiatry 1998; 68: 216-232.
Velligan DI, Weiden PJ, Sajatovic M, Scott J, Carpenter D, Ross R, Docherty JP; Expert Consensus Panel on Adherence Problems in Serious and Persistent Mental Illness. The expert consensus guideline series: adherence problems in patients with serious and persistent mental illness. J Clin Psychiatry 2009; 70 Suppl 4: 1-46.

2.7 Bei Multicenter-Studien (LKP in Hamburg)

a) Ist das Projekt schon in einem anderen Bundesland bei der für den dortigen Prüfarzt zuständigen Ethik-Kommission vorgelegt worden? Wenn ja, bei welcher?

Entfällt

b) Wie viele Zentren nehmen teil?
Die folgenden Zentren nehmen an der Durchführung der Studie teil:

Es handelt sich um eine monozentrische Studie

2.8 Geplanter Studienzeitraum (Beginn/Ende)

‚First patient in': 01.05. 2007. Gesamtdauer der Studie: fortlaufend

3 Schilderung der voraussehbaren Belastungen und Risiken für die Versuchspersonen

Mit der Studienteilnahme verbunden ist eine standardisierte Behandlung mittels IV & ACT. Aufgrund des aktuellen wissenschaftlichen Forschungsstandes kann nicht davon ausgegangen werden, dass IV & ACT gegenüber der aktuell üblichen Standardbehandlung für die Patienten mit besonderen Risiken oder Belastungen verbunden ist. Die Datenerhebung zur Qualitätssicherung für die beteiligten Krankenkassen wird im Rahmen der Regelversorgung durchführt, so dass den Patienten kein zusätzlicher Zeitaufwand entsteht.

3.1 Ein- und Ausschlusskriterien

Personen, die die Indikationen zur Teilnahme am Hamburger Modell erfüllen, werden bei erfolgter Einwilligung in die Behandlung in die vorliegende Studie eingeschlossen.

3.2 Stellungnahme zur Möglichkeit weiterer, derzeit nicht überschaubarer Risiken

Aufgrund des aktuellen Forschungsstandes ist nicht davon auszugehen, dass sich durch die Behandlung der Studienpatienten durch das Hamburger IV Modell neue oder nicht überschaubare Risiken ergeben könnten.

3.3 Abbruchkriterien (individuell und für die Gesamtstudie)

Im Zuge der Aufklärung über die Behandlung in der integrierten Versorgung werden alle Teilnehmer darüber informiert, dass sie sich jederzeit und ohne Angabe von Gründen gegen die weitere Teilnahme entscheiden können, ohne dass ihnen dabei Nachteile entstehen. Die integrierten Versorgung ist in die klinische Routineversorgung integriert. Beim Wunsch nach einem Wechsel des Behandlers bzw. der Behandlungsform werden die Patienten beim Wechsel zu einem Therapeuten ihrer Wahl unterstützt.

Da die Datenerhebung zur Qualitätssicherung der integrierten Versorgung nicht mit Risiken für die Patienten verbunden ist, entfällt die Spezifizierung von Abbruchkriterien.

4 Darlegung der voraussichtlichen Vorteile und der Bedeutung des Versuchs für den Menschen

a) In der Heilkunde

   Aufgrund der Erkenntnisse über Vor- und Nachteile von IV & ACT ist langfristig eine Optimierung der Behandlung dieser Patienten zu erwarten.

b) In der Forschung

   Aufgrund von methodischen Problemen und der geringen Anzahl von Vorstudien sowie des Fehlens von Informationen bezüglich der Wirksamkeit von IV & ACT bei Psychose Betroffenen im Deutschen Gesundheitssystem ist von der vorliegenden Analyse ein Wissensgewinn hinsichtlich der Wirksamkeit und Finanzierbarkeit von IV & ACT zu erwarten.

4.1 Bei minderjährigen, gesunden Versuchspersonen

Entfällt

4.2 Bei Patienten als Versuchspersonen

Mit der Datenauswertung entstehen den Patienten keine zusätzlichen Risiken und keine zusätzliche zeitliche Belastung. Die koordinierte qualitätsgesicherte integrierte Behandlung auf hohem Niveau (IV) mit zusätzlichen Behandlungsangeboten (ACT), einer aufwändigeren Diagnostik, sowie durch regelmäßige Kontrollen des Behandlungsergebnisses und Follow-up-Untersuchungen wird im Rahmen der Regelversorgung bereits zur Verfügung gestellt.

5 Güterabwägung zwischen den Nachteilen und Risiken einerseits und dem voraussichtlichen Nutzen andererseits

Es sind keine Belastungen und Risiken mit der vorliegenden Untersuchung verbunden, da die Daten im Rahmen der Qualitätssicherung der Regelversorgung erhoben werden und alle Analysen für die vorliegende Studie anonymisiert durchgeführt wird.

5.1 Berücksichtigung des Grundsatzes, dass stets die Belange der Versuchsperson den Vorrang haben

Entsprechend der Deklaration von Helsinki von 1996 ist gewährleistet, dass zu jedem Zeitpunkt der Behandlung die Belange der Patienten Vorrang haben. Bei Nicht-Einwilligung erwachsen den Patienten und Probanden keinerlei Nachteile. Ebenso kann die Einwilligung zur Teilnahme an der Behandlung zu jedem Zeitpunkt zurückgezogen werden ohne dass den Patienten dadurch ein Nachteil entsteht.

5.2 Beschränkung der Zahl der Versuchspersonen auf das unbedingt notwendige Maß

Die Zahl der Versuchspersonen ist aufgrund der Einbettung in das bestehende Versorgungssystem nicht beschränkt.

5.3 Bei Versuchen an gesunden Probanden, denen keine therapeutischen Vorteile aus dem Versuch erwachsen, gelten strengere Anforderungen an die Vertretbarkeit des Forschungsvorhabens als bei neuartigen Heilversuchen an Patienten

Entfällt

5.4 Besondere Überlegungen sind bei Blind- und Doppelblindversuchen an Patienten angesichts der ungleichen Behandlung der beiden Gruppen nötig

Entfällt

6 Angaben über den Inhalt der Aufklärungsgespräche mit den Versuchspersonen

6.1 Bei Probanden

a) Diagnoseaufklärung

Die eingeschlossenen Patienten werden bereits durch die teilnehmenden Einrichtungen medizinisch versorgt. Eine Aufklärung über die Diagnose ist daher im Rahmen der klinischen Routineversorgung durch die zuweisende Einheit (Station, Ambulanz oder Poliklinik) erfolgt. Bei Rückfragen der Patienten über ihre Diagnose werden die Patienten nach den Regeln der ärztlichen Kunst von dem ihnen zugeordneten Arzt beraten.

b) Alternative Verlaufserklärung (Prognoseaufklärung)

Eine spezielle Prognoseaufklärung hinsichtlich der Interventionen entfällt. Bei Rückfragen der Patienten über ihre Prognose werden die Patienten nach den Regeln der ärztlichen Kunst von dem ihnen zugeordneten Arzt beraten.

c) Risikoaufklärung, bezogen auf die Versuchsauswirkungen

Alle Probanden werden über die Durchführung der Qualitätssicherung im Rahmen der integrierten Versorgung aufgeklärt (siehe Anlagen 4 und 5).

d) Aufklärung über ein angebrachtes Verhalten der Versuchsperson während des Versuches und nach dem Versuch

Alle Probanden werden über das erforderliche Verhalten während der Untersuchung informiert. Eine Teilnahme an der integrierten Versorgung ist erst

nach schriftlicher Einwilligung durch Unterzeichnung des Aufklärungsbogens möglich.

e) Aufklärung über die Aufzeichnung von Krankheitsdaten und deren Weitergabe zur Überprüfung an den Auftraggeber, die zuständige Überwachungsbehörde (gemäß § 67 AMG) oder die zuständige Bundesoberbehörde [§ 40 (1) 2. AMG; § 20 (1) 2. MPG]
Alle Probanden werden darauf hingewiesen, dass die für die Qualitätssicherung der integrierten Versorgung erfassten medizinischen Daten in anonymisierter Form verarbeitet und veröffentlicht werden.

f) Studienbedingte röntgenologische/nuklearmedizinische Untersuchungen
Entfällt

6.2 Aufklärung über die Widerruflichkeit einer Einwilligung

Die Patienten werden darauf hingewiesen, dass sie ihr Einverständnis für die Behandlung in der integrierten Versorgung jederzeit ohne Angabe von Gründen und ohne weitere Folgen zurückziehen können.

6.3 Besondere Aufklärung über die Situation

a) bei der randomisierten Studie
Entfällt

b) beim Blind- und Doppelblindversuch
Entfällt

c) bei placebo-kontrollierten Studien
Entfällt

6.4 Beachtung der Sonderregeln bei Minderjährigen und bei in ihrer Geschäftsfähigkeit beeinträchtigten Patienten

Entfällt

7 Beifügung eines Musters für die (schriftlich) zu erteilende Aufklärung/Einwilligungserklärung [§ 40 (2) Nr. 2 AMG]

Die verwendeten Aufklärungsbögen und Einwilligungserklärungen liegen dem Antrag als Anlagen 4 und 5 bei.

8 Nachweis einer ausreichenden Probandenversicherung

Der Gesetzgeber und die Versicherungsträger haben den Abschluss einer Studienversicherung (Gefährdungshaftung) für Studien zur Prüfung eines neuen Arzneimittels oder Medizinprodukts oder eines neuen Anwendungsgebietes vorgesehen. Bei der vorliegenden Studie handelt es sich nicht um eine klinische Prüfung im Sinne von § 4 (23) AMG.

Im Rahmen der Teilnahme an der genannten Behandlung gelten die allgemeinen Haftungsgrundsätze. Im Fall eines Schadens durch die Behandlung besteht Deckung durch die Haftpflichtversicherung des UKE. Die Teilnehmer der integrierten Versorgung werden in der Patienteninformation und den Einwilligungsunterlagen auf diesen Sachverhalt hingewiesen.

8.1 Aufklärung über das Bestehen und den Umfang einer Probandenversicherung und die danach von der Versuchsperson zu beachtenden Obliegenheiten im Falle des vermuteten Schadens und im Todesfall

Vgl. 8

9 Darlegung der Erfüllung etwaiger sonstiger Voraussetzungen für die Durchführung des Vorhabens

Entfällt

10 Maßnahmen bei Veränderung der Risikolage

10.1 Sicherstellung, dass bei Veränderungen der Risikolage während des Versuchs die Güterabwägung im Sinne von Ziffer 5 jeweils erneut durchgeführt wird und bei nachteiliger Veränderung der Risikolage auch erneute Aufklärungsgespräche mit den Versuchspersonen stattfinden

Entfällt, da Bestandteil der Regelversorgung.

10.2 Mitteilung der veränderten Abbruchkriterien an die Ethik-Kommission

Entfällt, da Bestandteil der Regelversorgung.

10.3 Mitteilung schwerwiegender oder unerwarteter Ereignisse an die Ethik-Kommission.

Entfällt, da Bestandteil der Regelversorgung.

Hamburg, den 25. Januar 2012

____

PD Dr. med. Anne Karow

____

Dr. med. Daniel Schöttle

11 Anlagen

Anlage 1 Studienprotokoll

Anlage 2 Lebenslauf des Antragstellers

Anlage 3 Schriftenverzeichnis des Antragstellers

Anlage 4 Einwilligungserklärung

Anlage 5 Patienteninformation

Anlage 1 Studienprotokoll (Version 1 vom Januar 2012)

Integrierte Versorgung nach dem Hamburger Modell bei Patienten mit Psychosen – Vergleich des klinischen Verlaufs in der Regelversorgung mit einer historischen Kontroll-gruppe

Studienprotokoll

Januar 2012

Studienprotokoll	
**Studienleiter**	PD. Dr. Anne Karow, Klinik und Poliklinik für Psychiatrie und Psychotherapie, Zentrum für Psychosoziale Medizin, Universitätsklinikum Hamburg-Eppendorf, Martinistr. 52, 20246 Hamburg, Tel: 040 / 7410-57483; Fax: 040 / 7410-55455; E-Mail: karow@uke.de
	Dr. Daniel Schöttle, Klinik und Poliklinik für Psychiatrie und Psychotherapie, Zentrum für Psychosoziale Medizin, Universitätsklinikum Hamburg-Eppendorf, Martinistr. 52, 20246 Hamburg, Tel: 01522-2816853; Fax: 040 / 7410-55455; E-Mail: d.schoettle@uke.de
**Titel der Studie**	Integrierte Versorgung nach dem Hamburger Modell bei Patienten mit Psychosen – Vergleich des klinischen Verlaufs in der Regelversorgung mit einer historischen Kontrollgruppe
**Kurzfassung**	Verbesserte Behandlung durch die Errichtung eines Behandlungsnetzwerkes und integrierter Versorgung nach dem Hamburger Modell mit Home Treatment und Integriertem Behandlungsnetzwerk, soll die Prognose schwer psychoseerkrankten Menschen nachhaltig verbessern.
**Studienpopulation**	Erwachsene Menschen mit einer psychotischen Erkrankung, einschließlich Schizophrenie Spektrums Erkrankungen, Bipolar I. und II. Störungen, Major Depression mit psychotischen Merkmalen.
**Ziel (e)**	**Primäres Studienziel:** Führt die Integrierte Versorgung (IV) mit 'Assertive Community Treatment' und Home Treatment nach dem Hamburger Modell im Vergleich zu einer historischen Kontrolle (ACCESS Studie) zu einer vergleichbar hohen psychofunktionalen Remissionsrate im Behandlungsverlauf?<br>**Sekundäre Studienziele: A.** Führt IV zu einer Verbesserung des Outcomes hinsichtlich Psychopathologie, Funktionsniveau und Lebensqualität? **B.** Führt IV zu einer Verbesserung der Inanspruchnahme der Behandlungsangebote, der Medikamenten-Adhärenz und der Behandlungszufriedenheit der Patienten und Angehörigen? **C.** Ist IV eine kosteneffektive Behandlung?
**Interventionen**	<u>Experimentalbedingung:</u> Integrierte Behandlung (Integrierte Versorgung, IV)<br><u>Kontrollbedingung:</u> Historische Kontrolle: n=120 Patienten mit Psychosen, die zwischen 1/2006 und 12/2007 an der ACCESS Studie teilgenommen hatten und eine Einverständniserklärung für die Verwendung ihrer Daten unterschrieben haben.
**Ein- und Ausschlusskriterien**	<u>Einschlusskriterien</u>:<br>▪ Männer und Frauen, Alter ab 18 Jahre mit ausreichenden Kenntnissen der deutschen Sprache<br>▪ Vorliegen einer der folgenden Diagnosen nach ICD-10: Schizophrenie (F20 einschließlich aller Subtypen), anhaltende wahnhafte Störung (F22), akute vorübergehende psychotische Störung (F23), induzierte wahnhafte Störung (F24), schizoaffektive Störung (F25), sonstige nichtorganische psychotische Störung (F28), nicht näher bezeichnende nichtorganische Psychose (F29), Substanzinduzierte psychotische Störung (F1x.5), Manische Episode mit psychotischen Symptomen (F30), Bipolar affektive Störung (F31), Schwere depressive Episode mit psychotischen Symptomen (F32.3), Rezidivierende depressive Störung, gegenwärtig schwere depressive Episode mit psychotischen Symptomen (F33.3)<br><u>Ausschlusskriterien</u>:<br>▪ Psychotische Störung aufgrund einer organischen Erkrankung (z.B. F06.0-3)<br>▪ Wohnort außerhalb des Sektors
**Studientyp**	Quasi-experimentelle, klinische Verlaufsstudie mit historischer Kontrollgruppe
**Messzeitpunkte**	Aufnahme (t0), 6 Wochen (t1), 3 Monate (t1), 6 Monate (t2), alle 6 Monate fortlaufend
**Primäre Zielgröße**	Das primäre Zielkriterium ist die Remissionsrate über einen Zeitraum von mindestens 12 Monaten. Eine symptomatische Remission liegt vor, wenn die schizophrene Kernsymptomatik einen leichten bis milden Schweregrad erfüllt (siehe Andreasen et al. 2005). Eine funktionale Remission ist über einen GAF-Wert ≥ 60 operationalisiert. Eine psychofunktionale Remission ist operationalisiert über ein zeitgleiches Erfüllen des symptomatischen und funktionalen Remissionskriteriums über einen zeitpunkt von mindestens sechs Monaten.
**Sekundäre Zielgrößen**	Psychopathologie (BPRS, CGI-S, CGI-BP), Funktionsniveau (GAF), Lebensqualität (Q-LES-Q-18, EQ5-D), Service engagement (SES), Medikamenten-Adhärenz, Behandlungszufriedenheit (CSQ-8), Gesundheitsökonomie,
**Statistische Analysen**	<u>Primärer Endpunkt:</u> Als primäre statistische Analyse wird ein exakter Test nach Fisher zweiseitig zum 5% Niveau verwendet, mit dem Prozentsatz der Patienten in psychofunktionaler Remission nach 24-monatiger Behandlung und im Langzeitverlauf in beiden Behandlungsgruppen.<br><u>Sekundäre Endpunkte:</u> Für den Vergleich dimensionaler sekundärer Outcomeparameter, z.B. Psychopathologie werden Kovarianzanalysen berechnet. Die Kosten-Effektivität wird nach der Methode von Knapp et al. (2008) mittels Quality-Adjusted Life Years (QALYs) berechnet.

1. ZUSAMMENFASSUNG

Im Rahmen der vorliegenden quasi-experimentellen Studie soll die Wirksamkeit der integrierten Versorgung nach dem Hamburger Modell (IV) als Bestandteil der Regelversorgung im Vergleich zu einer historischen Kontrollgruppe untersucht werden. Für die Hauptfragestellung sollen Patienten in der IV-Intervention nach dem Hamburger Modell mit einer historischen Kontrollgruppe (ACCESS Studie) hinsichtlich ihrer psychofunktionalen Remissionsrate im Langzeitverlauf verglichen werden. Sekundäre Erfolgskriterien umfassen die Verbesserung der Krankheitssymptome, des Funktionsniveaus, der Lebensqualität, die Einhaltung der Medikation, die Zufriedenheit des Patienten mit der Behandlung, die Kosteneffektivität der Behandlung und die Sicherheit der Behandlung (Selbst-/Fremdgefährdung). Bei der historischen Kontrollgruppe handelt es sich um Patienten aus der ACCESS Studie, die die Integrierte Versorgung bei Patienten mit Psychosen unter kontrollierten Bedingungen über 12 Monate untersuchte (Karow et al., in press; Lambert et al., 2010). Die vorliegende Studie erweitert die Ergebnisse dieser Referenzuntersuchung um Daten aus der Regelversorgung („real life“ Bedingungen) und kann somit zu einer nachhaltigen Implementierung der IV in die Regelversorgung in Deutschland beitragen.

Die klinische Auswertung soll anhand von Qualitätssicherungsdaten aus dem Hamburger Modell für Integrierte Versorgung der Klinik für Psychiatrie und Psychotherapie des Universitätsklinikums Hamburg-Eppendorf durchgeführt werden. Die vorliegenden Qualitätssicherungsdaten werden seit Beginn der Integrierten Versorgung nach dem Hamburger Modell für die beteiligten Krankenkassen erhoben.

2. Hintergrund

Psychosen stellen ernsthafte, hochkomplexe psychische Erkrankungen dar, die derzeit häufig durch eine schlechte Prognose mit wiederholten psychotischen Episoden (über 80%aller Betroffenen) und einer hohen Chronifizierungsrate gekennzeichnet sind. Darüberhinaus sind Psychosen keine seltenen Erkrankungen: Nach der letzten großen deutschen epidemiologischen Untersuchung haben Psychosen eine 1-Jahres-Prävalenz von 2.6% und eine Lebenszeitprävalenz von 4.5% (Jacobi et al., 2004). Verlauf und Prognose psychotischer Störungen hängen maßgeblich von der Qualität der Behandlung (Conus et al. 2007, Lambert & Naber 2009).

1. Komplexität der Erkrankung

Psychosen sind zumeist komplexe Störungen: Diese Komplexität ergibt sich aus der Vielzahl von psychotischen (positiv, negativ, kognitiv, Desorganisation) und assoziierten (Agitation, Depression, Suizidalität) Symptomen, komorbiden psychischen oder somatischen Störungen, Stressoren, Konflikten, Traumata, verzögerten Persönlichkeitsentwicklungen, Funktionseinbußen und daraus resultierenden sozialen Problemen wie Abbruch von Schule, Ausbildung, Arbeit oder Kontakten zu Familie oder Freunden. Viele der genannten Faktoren (z.B. Suchtkomorbidität oder Traumata) können zu einer schlechten Prognose beitragen, insbesondere wenn sie nicht adäquat diagnostiziert und langfristig behandelt werden. (Lambert & Naber 2009; Conus et al. 2010b).

2. Eine Unzureichende Qualität der Behandlung von Psychosen

(a) Versorgungsspezifische Rationalen für die Entwicklung des Hamburger Modells:

Im traditionellen Behandlungssystem zeigen nur 15% aller Psychose Betroffenen einen guten Behandlungserfolg mit psychofunktionaler Remission (Rosen & Garety, 2005). Mehr als 80% haben wiederholte psychotische Episoden, dauerhafte Beeinträchtigungen des Funktionsniveaus, verüben wiederholt Suizidversuche und haben insgesamt eine um etwa 20 Jahre verkürzte Lebenserwartung (Lambert & Naber, 2009). Der Verlauf der Erkrankung ist häufig ungünstig: Neben der Komplexität der Erkrankung und der langen Behandlungsverzögerung ist hierfür auch ein unzureichenden Hilfesystems maßgeblich: Neben fehlender bzw. unzureichender Aufklärung und Früherkennung besteht derzeit zumeist eine unzureichende

ambulante psychiatrische Behandlung für Psychosepatienten, bedingt durch die Tatsache, dass der Fokus des Versorgungssystems derzeit auf vollstationäre Behandlungsformen ausgerichtet ist. Darüber hinaus ist eine Überfinanzierung ambulanter Psychotherapien im Verhältnis zur Finanzierung von ambulanter psychiatrischer Versorgung zu verzeichnen. Der Zugang zum Hilfesystem ist in der bestehenden Form zu hochschwellig mit langen Wartezeiten bis zum Therapiebeginn und fehlender Implementierung von intensiven ambulanten Behandlungsmodellen. So ergab eine Untersuchung an 902 Psychose-Patienten, dass etwa 70% der Betroffenen lediglich ein bis drei Termine pro Quartal als Therapie erhielten, die im Durchschnitt 5-30min dauerten. Zudem erhielten nur etwa 8-10% der Patienten, die für sie notwendigen evidenz-basierten Therapien und nur etwa 7% der schwer erkrankten Patienten hatten jemals seit Beginn ihrer Erkrankung eine Psychotherapie erhalten (Lambert et

al.). Obwohl es bereits hohe Evidenz für die Wirksamkeit von intensiven ambulanten Behandlungsmodellen wie Assertive Community Treatment (ACT) bei schwer erkrankten Patienten gibt (Lambert et al.) (Marshall & Lockwood), werden diese Behandlungsmodelle in Deutschland bisher kaum angeboten. Auch die notwendige Kombination von medikamentöser und psychsosozialer wird kaum umgesetzt; laut neuesten europäischen Studien erhalten nur etwa 8% der Psychose-Betroffenen eine kombinierte medikamentöse und psychosoziale Behandlung (WHO 2011).

(b) Versorgungsspezifische Rationalen für die Entwicklung des Hamburger Modells:

Psychotische Erkrankungen verursachen für die Gesellschaft immense Kosten, die derzeit allein in Deutschland auf etwa 5 Milliarden € pro Jahr geschätzt werden (Lambert & Naber, 2009), beziehungsweise etwa 14.000-18.000€ pro Patient (Konnopka et al. 2009). Hinzu kommen Kosten für Angehörige von 950-1.700€ und indirekte Kosten von 25.000-30.000€, so dass die Gesamtkosten bei etwa 39.950-49.700€ pro Jahr pro Patient liegen. Betrachtet man alle Patienten aller Schweregrade zusammen, entstehen 60-70% der direkten Kosten durch (wiederholte) stationäre Behandlungen; mit steigendem Schweregrad der Erkrankung steigt dieser Anteil auf bis 90%.

Die Effektivität einer frühen, intensiven, integrierten und aufsuchenden gemeindepsychiatrischen Behandlung (Assertive Community Treatment, ACT) konnte bereits in anderen Ländern belegt werden. ACT-Teams sind multiprofessionell und initiieren und koordinieren die verschiedenen Behandlungselemente: ärztlich/psychologische Behandlung, psychiatrische Pflege, Psychoedukation, Familieninterventionen, Suchtinterventionen, Rehabilitationsarbeit sowie andere individuell notwendige Behandlungsmaßnahmen. Mehrere Studien liefern empirische Unterstützung, dass eine integrierte Behandlung im Vergleich zu einer Standardbehandlung die Langzeitprognose des psychofunktionalen Krankheitsverlaufs verbessert und aufgrund seltenerer Krankenhausaufenthalte deutlich niedrige direkte Krankheitskosten verursacht (Henry & McGorry, 2007; Mihalopoulos, Harris, Henry, Harrigan, & McGorry, 2009; Petersen et al., 2005).

3. Studiendesign

Im Rahmen einer klinischen Vergleichsstudie soll untersucht werden, ob sich Patienten mit einer IV-Intervention nach dem Hamburger Modell im Vergleich zu einer his-

torischen Kontrollgruppe hinsichtlich ihrer psychofunktionalen Remissionsrate im Langzeitverlauf unterscheiden.

3.1 Intervention

Das „Hamburger Modell" bezeichnet ein Integriertes Versorgungsmodell für schwer erkrankte Psychose Betroffene nach § 140 SGB V, dass eine sektorübergreifende und langfristige Behandlung in einem Netzwerk bestehend aus dem Arbeitsbereich Psychosen des UKE und niedergelassenen Psychiatern beinhaltet. Das Modell läuft seitdem 1.5.2007, folgende Krankenkassen nehmen daran teil: DAK, HEK, IKK Classic und AOK Rheinland Hamburg. Für die Teilnahme bestehen krankenkassenspezifische und diagnostische Indikationen sowie Indikationen, die einen hohen Schweregrad der Erkrankung abbilden. Das Behandlungsmodell umfasst als Kernbestandteil sog. Assertive Community Treatment (ACT), ein evidenzbasiertes Behandlungsmodell für schwer erkrankte Patienten. Darüber hinaus können die Teilnehmer alle Angebote des Arbeitsbereich Psychosen und der beteiligten niedergelassenen Psychiater nutzen. Das Hamburger Modell ist ein sog. „Capitation-Modell", d.h. das UKE als Hauptvertragsnehmer bekommt eine versichertenbezogene Jahrespauschale, mit der alle Leistungen finanziert werden.

Ziel des Hamburger Modells ist eine langfristige Verbesserung der Behandlung schwer erkrankter Psychosepatienten:
Im Zentrum der Behandlung steht das ACT-Team, das im Wesentlichen folgende Aufgaben hat:

- Mobiles zu Hause Behandlungsteam,
- Planung und Koordination aller Interventionen,
- Hochfrequente, langfristige und settingübergeifende Behandlung im eigenen Umfeld inklusive Akut- und Langzeitbehandlung, Pharmakotherapie, Psychotherapie und Beteiligung des sozialen Umfelds,
- Verhinderung von Rückfällen durch Krisenintervention 7 Tage die Woche und 24h täglich, sofortige Rückfallbehandlung, Interventionen bei Non-Compliance und (möglichen) Gesamtbehandlungsabbruch,
- Bei stationären Behandlungen, poststationäre intensive Nachsorge.
- Jeder im Netzwerk erkannte Betroffene und seine Angehörigen können neben dem ACT Team – und damit deren Rufbereitschaft (7 Tage die Woche und

24h täglich) – alle zur Verfügung stehenden Institutionen und Angebote nutzen, insbesondere solche der Spezialambulanz Psychosen (SPA), wie z.B. Kontakt zu ausgebildeten Peer-experts, Selbsthilfegruppen und Metakognitives Training.

Das ACT-Team nach dem Hamburger Modell stellt im Gegensatz zu konventionellen Modellen ein therapeutisches ACT dar, dass heißt, es ist auf die Behandlung von Psychosen spezialisiert, multiprofessionell besetzt (u.a. Fachärzte, Therapeuten, Sozialarbeiter) und speziell ausgebildet für die Pharmako- und Psychotherapie von Psychosen. Es zeichnet sich zusätzlich durch eine niedrige Behandler-Patienten-Ratio von 1:10 bis maximal 1:20 und „no drop out policy" aus.

Darüberhinaus wird durch das Hamburger Modell die Vernetzung ambulanter Ressourcen insbesondere von Institutsambulanz, niedergelassenen Ärzten und Psychosozialer Kontaktstelle (Gemeindepsychiatrischen Zentrum Eimsbüttel, GPZE) verbessert und das Hilfssystem für Erkrankte so transparenter und weniger hochschwellig gestaltet, sowie eine setiingübergreifende Behandlungskontinuität gewährleistet. Die 20 niedergelassenen Psychiater, die am Hamburger Modell teilnehmen, haben sie sich dazu verpflichtet, möglichst keine oder nur sehr kurze Wartezeiten vorzuhalten und diese Patienten intensiver als bisher zu betreuen, sowie regelmäßig an den intersektoralen Fallkonferenzen inklusive der Vorbereitung der Kasuistiken teilzunehmen und einer zusätzlichen Dokumentation im Rahmen der Integrierten Versorgung zur begleitenden Qualitätssicherung zugestimmt.

Kontrollbedingung

Die Kontrollbedingung ist eine historische Kontrollgruppe von 120 erwachsenen Patienten mit Psychosen, die in dem Zeitraum vor Implementierung der Integrierten Versorgung am Universitätsklinikum Hamburg-Eppendorf, an der ACCESS-Studie teilgenommen haben und eine Einverständniserklärung für die Verwendung ihrer Daten unterschrieben hatten. Die ACESS wurde im Zeitraum von 01/2006 bis 01/2007 am UKE und in Rissen durchgeführt und diente als Referenzstudie für die Einführung der IV nach dem Hamburger Modell („Assertive Community Treatment as part of Integrated Care vs. Standard Care in patients with Schizophrenia„ EudraCT number: 2005-001069-32; Clinicaltrials.gov number: NCT01081418; Nummer Ethik Kommission Hamburg: 2515).

3.2 Stichprobe, Einschlußkriterien, Ausschlußkriterien

Alle Patienten, die sich zur Teilnahme an der IV bereit erklären, werden ausführlich über den Sinn der Behandlung und die Datenerhebung zur Qualitätssicherung informiert und auf die Freiwilligkeit der Teilnahme und die Anonymisierung der Daten hingewiesen. Für eine Teilnahme an der vorliegenden Auswertung müssen die Patienteninformation und die Einwilligungserklärung unterschrieben worden sein (siehe Anlagen x und y). Es müssen folgende Ein- und Ausschlusskriterien erfüllt sein:

Einschlusskriterien

Um in die IV Behandlung eingeschlossen zu werden, müssen die Patienten alle der folgenden Kriterien erfüllen:

- Männer und Frauen
- Alter ab 18 Jahre
- Ausreichende Kenntnisse der deutschen Sprache
- Mitgliedschaft in einer der folgenden Krankenkassen (HEK, IKK Classic, DAK, AOK Rheinland-Hamburg)
- Krankenhauseinweisung
- Vorliegen einer der folgenden Diagnosen nach ICD-10: Schizophrenie (F20 einschließlich aller Subtypen), anhaltende wahnhafte Störung (F22), akute vorübergehende psychotische Störung (F23), induzierte wahnhafte Störung (F24), schizoaffektive Störung (F25), sonstige nichtorganische psychotische Störung (F28), nicht näher bezeichnende nichtorganische Psychose (F29), Substanzinduzierte psychotische Störung (F1x.5), Manische Episode mit psychotischen Symptomen (F30), Bipolar affektive Störung (F31), Schwere depressive Episode mit psychotischen Symptomen (F32.3), Rezidivierende depressive Störung, gegenwärtig schwere depressive Episode mit psychotischen Symptomen (F33.3)
- Erfüllung mindestens eines der folgenden Schweregradkriterien:

Vorliegende Einweisung:	Einweisung zur stationären Aufnahme von einem niedergelassenen Psychiater oder bei Notfallaufnahme von einem Krankenhausarzt liegt vor. Die Schwere der Erkrankung macht normalerweise eine stationäre Behandlung notwendig.
Erfüllung des allgemeinen Schwere-grad-kriteriums:	Der Patient erfüllt einen allgemeinen Schweregrad von einem Gesamtwert von mindestens 40 Punkten in der Brief Psychiatric Rating Scale (BPRS) Chronizitätskriterium hinzu (hatte in den letzten 2 Jahren viele (teil-)stationäre Aufenthalte)

Erfüllung mindestens eines der spezifischen Schweregrad-kriterien:	Halluzinationen (item 10) ≥ 6 Punkte<br>Wahn (item 11) ≥ 6 Punkte<br>Desorganisation (item 15) ≥ 6 Punkte<br>Depressiv-suizidales Syndrom ≥ 10 Punkte<br>Suizidales Syndrom ≥ 6 Punkte<br>Manisches Syndrom ≥ 15 Punkte<br>Verhaltensstörungs-Syndrom im Rahmen einer Psychose ≥ 15 Punkte<br>Syndrom vorherrschender Negativsymptomatik ≥ 15 Punkte

Ausschlusskriterien

- Vorliegen einer der folgenden Diagnosen nach ICD-10: Psychotische Störung aufgrund einer organischen Erkrankung (z.B. F06.0-3)
- Wohnort außerhalb des Sektors

3.2 Studienablauf, Untersuchungen und Erhebungszeitpunkte

Die Untersuchungen im Rahmen der Qualitätssicherung der IV erfolgen in einem ruhigen Raum in der Psychiatrie des UKE. Eine Untersuchung dauert etwa eine halbe bis eine Stunden und wird von einer Psychologin durchgeführt. Einen Überblick über die Hauptmesszeitpunkte und eingesetzten Instrumente gibt Tabelle 1.

Tabelle 1. Überblick über die im Laufe der Behandlung durchgeführten Untersuchungen

Untersuchungsparameter	Skalen und Erklärung	Evaluation Zeitpunkt(e) in Wochen (A = Aufnahme)
**Basisdaten**		
**Demographie und klinische Variablen**	Early Psychosis File Questionnaire (EPFQ; Lambert et al. 2005)	A
**Diagnostische Variablen**	Hauptdiagnose, Komorbidität (bei Aufnahme und im Verlauf), Suizidversuchsdiagnosen	A
**Compliance**		
**Non-Compliance (objektiv)**	≥ 1 Woche ohne Medikation (Robinson et al. 2005)	A, Woche 6, Monat 3 Monat 6, alle 6 Monate, fortlaufend
**Partielle Compliance (objektiv)**	Verpasst 20-80% der Medikation (Kane et al. 2003)	A, Woche 6, Monat 3 Monat 6, alle 6 Monate, fortlaufend
**Gesamtbehandlungsabbruch und Behandlungseinbindung**		
**Gesamtbehandlungsabbruch**	Gesamtbehandlungsabbruch ist evident, wenn der Patient wiederholt trotz “no drop out policy” die weitere Behandlung ablehnt oder trotz vielfacher Versuche nicht mehr kontaktierbar ist.	A, Woche 6, Monat 3 Monat 6, alle 6 Monate, fortlaufend
**Behandlungseinbindung**	Service Engagement Scale (SES; Tait et al. 2005; Werte von 0-3 Punkten; Durchschnittswert über 14 Fragen; niedrigere Werte = besseres Engagement)	A, Woche 6, Monat 3 Monat 6, alle 6 Monate, fortlaufend
**Psychopathologie und Schwere der Erkrankung**		
**BPRS**	Brief Psychiatric Rating Scale (BPRS; Overall & Gorham, 1962; Werte von 0-144 Punkte; niedrigere Werte = bessere Psychopathologie)	A, Woche 6, Monat 3 Monat 6, alle 6 Monate, fortlaufend
**CGI-S**	Clinical Global Impression Scale (Guy et al. 1976; Werte von 1-7 Punkten; niedrigere Werte = niedrigere Schwere der Erkrankung)	A, Woche 6, Monat 3 Monat 6, alle 6 Monate, fortlaufend
**CGI-BP**	Clinical Global Impression Scale – Bipolar Disorder (CGI-BP; Spearing et al. 1997; Werte von 1-7 Punkten; niedrigere Werte = niedrigere Schwere der Erkrankung; 2 Subskalen für depressive und manische Symptome)	A, Woche 6, Monat 3 Monat 6, alle 6 Monate, fortlaufend

**Lebensqualität**		
**Q-LES-Q-18**	Quality of Life Enjoyment and Satisfaction Questionnaire (Ritsner et al. 2005; 18 Fragen; Werte von 0-5; Durchschnittswert über 18 Fragen; höhere Werte = besseres Lebensqualität)	A, Woche 6, Monat 3 Monat 6, alle 6 Monate, fortlaufend
**EQ-5D**	Euro-QoL 5D (Prieto et al. 2004; Werte von 0-5 Punkten; niedrigere Werte = niedrigere Lebensqualität)	A, Woche 6, Monat 3 Monat 6, alle 6 Monate, fortlaufend
**Behandlungszufriedenheit**		
**CSQ-8 Patient**	Client Satisfaction Questionnaire (CSQ-8; Larsen et al. 1979; Werte von 8 bis 32, höhere Werte = höhere Behandlungszufriedenheit)	A, Woche 6, Monat 3 Monat 6, alle 6 Monate, fortlaufend
**CSQ-8 Familie/Angehörige**	Client Satisfaction Questionnaire (CSQ-8; Larsen et al. 1979; Werte von 8 bis 32, höhere Werte = höhere Behandlungszufriedenheit)	A, Woche 6, Monat 3 Monat 6, alle 6 Monate, fortlaufend
**Daten zur Intervention**		
**Dokumentation**	Ständige Dokumentation aller Leistungen (inkl. Anzahl, Art, Dauer; Bericht alle 3-6 Monate; Anzahl, Dauer, Art der Kontakte/Therapien	Ständige Dokumentation
**Kosten**		
**Dokumentation**	Krankenhauskosten: (voll- und teilstationär, Institutsambulanz), Ambulante Kosten, (ACT Team, niedergelassener Psychiater)	Ständige Dokumentation

EPFQ: Early Psychosis File Questionnaire (Conus, Cotton, Schimmelmann, McGorry, & Lambert, 2007); CGI-S: Clinical Global Impression Scale-Schizophrenia (Guy, 1976); GAF: Global Assessment of Functioning Scale (American Psychiatric Association, 2003); MLVI: Modified Location and Vocation Index (Tohen et al., 2000); Q-LES-Q-18: Quality of Life Enjoyment and Satisfaction Questionnaire (Ritsner, Kurs, Gibel, Ratner, & Endicott, 2005); CSQ-8: Client Satisfaction of Care Questionnaire (Nguyen, Attkisson, & Stegner, 1983); EQ-5D: EuroQol-5D (Prieto, Novick, Sacristán, Edgell, & Alonso, 2003);

3.3 Fragestellungen

Primäres Zielkriterium:

Es soll untersucht werden, ob eine langfristige IV Intervention mit ACT nach dem Hamburger Modell im Vergleich zu einer historischen Kontrollgruppe bei Psychose-Patienten mit einem hohen Schweregrad oder Chronifizierung der Erkrankung zu einer vergleichbar hohen der psychofunktionalen Remissionsrate führt.

Operationalisierung:

Das primäre Zielkriterium ist die Anzahl der Patienten in psychofunktionaler Remission über einen Zeitraum von mindestens sechs Monaten (von t1 zu t2). Eine symptomatische Remission liegt vor, wenn die schizophrene Kernsymptomatik einen leichten bis milden Schweregrad erfüllt (siehe Andreasen et al. 2005). Eine funktionale Remission ist über einen GAF-Wert ≥ 60 operationalisiert. Eine psychofunktionale Remission ist operationalisiert über ein zeitgleiches Erfüllen des symptomatischen und funktionalen Remissionskriteriums über einen Zeitpunkt von mindestens sechs Monaten. Alle Ergebnisse werden hinsichtlich Alter, Geschlecht, Dauer der Erkrankung und Schweregrad der Symptomatik zu Beginn der Behandlung kontrolliert.

Sekundäre Zielkriterien:

Es soll untersucht werden, ob eine Behandlung in der integrierten Versorgung nach dem Hamburger Modell bei Psychosepatienten zu einer Verbesserung des Outcomes führt hinsichtlich:

A. Psychopathologie, Funktionsniveau und Lebensqualität,

B. Inanspruchnahme der Behandlungsangebote (service engagement), Medikamenten-Adhärenz und Behandlungszufriedenheit der Patienten und

C. Zuwachs an QALY's innerhalb von 12 Monaten oder einem längeren Zeitraum führt.

3.4 Statistische Analysen

Primäres Zielkriterium: Als primäre statistische Analyse wird ein exakter Test nach Fisher zweiseitig zum 5% Niveau verwendet. Die abhängige Variable ist die prozentuale psychofunktionale Remissionsrate zum Follow-up nach 12 bzw. 24 Monaten in der Interventionsgruppe und 12 Monaten in der historischen Kontrolle. Alle Ergebnisse werden hinsichtlich Alter, Geschlecht, Dauer der Erkrankung und Schweregrad der Symptomatik zu Beginn der behandlung kontrolliert.

Sekundäre Zielkriterien A-C.: Zur Untersuchung der Frage, ob innerhalb der Interventionsgruppe mit IV Behandlung Interventionseffekte für die sekundären Outcomeparameter, z.B. Psychopathologie, vorliegen, werden Kovarianzanalysen und Varianzanalysen mit Messwiederholung berechnet. Die Kosten-Effektivität wird mittels Quality-Adjusted Life Years (QALYs) und einem Vergleich der Behandlungskosten überprüft (Knapp et al., 2008)

4. Referenzen

American Psychiatric Association. (2003). *Diagnostisches und Statistisches Manual Psychischer Störungen -Textrevision- DSM-IV-TR* (H. Saß, H. U. Wittchen, M. Zaudig & I. Houben, Trans.). Göttingen: Hogrefe.

Conus, P., Cotton, S., Schimmelmann, B. G., McGorry, P. D., & Lambert, M. (2007). The first-episode psychosis outcome study: premorbid and baseline characteristics of an epidemiological cohort of 661 first-episode psychosis patients. *Early Intervention in Psychiatry, 1*, 191-200.

Guy, W. (1976). *Clinical Global Impressions: in ECDEU Assessment Manual for Psychopharmacology*. Rockville, Maryland: National Institute for Mental Health.

Henry, L. P., & McGorry, P. (2007). Early psychosis prevention and intervention centre long-term follow-up study of first-episode psychosis: methodology and baseline characteristics. *Early Intervention in Psychiatry, 1*, 49-60.

Jacobi, F., Wittchen, H. U., Hölting, C., Höfler, M., Pfister, H., Müller, N., et al. (2004). Prevalence, comorbidity and correlates of mental disorders in the general population: results from the German Health Interview and Examination Survey (GHS). *Psychological Medicine, 34*, 597-611.

Karow, A., Reimer, J., König, H. H., Heider, D., Bock, T., Huber, C. G., et al. (in press). Cost-effectiveness of 12-month therapeutic Assertive Community Treatment as part of Integrated Care vs. Standard Care in patients with schizophrenia treated with Quetiapine IR (ACCESS Trial). *J Clin Psychiatry*.

Knapp, M., Windmeijer, F., Brown, J., Kontodimas, S., Tzivelekis, S., Haro, J. M., et al. (2008). Cost-utility analysis of treatment with olanzapine compared with other antipsychotic treatments in patients with schizophrenia in the pan-European SOHO study. *26*, 341-358.

Lambert, M., Bock, T., Schottle, D., Golks, D., Meister, K., Rietschel, L., et al. (2010). Assertive community treatment as part of integrated care versus standard care: a 12-month trial in patients with first- and multiple-episode schizophrenia spectrum disorders treated with quetiapine immediate release (ACCESS Trial). *J Clin Psychiatry, Oct;71(10)*, 1313-1323.

Lambert, M., & Naber, D. (2009). *Current Schizophrenia* – (2nd ed.): Current Medicine Group.

Marshall, M., & Lockwood, A. (2011). Assertive community treatment for people with severe mental disorders. *Cochrane Database Syst Rev*(4), CD001089.

Mihalopoulos, C., Harris, M., Henry, L., Harrigan, S., & McGorry, P. (2009). Is Early Intervention in Psychosis Cost-Effective Over the Long Term? *Schizophr Bull, 35*(5), 909-918.

Nguyen, T. D., Attkisson, C. C., & Stegner, B. L. (1983). Assessment of patient satisfaction: development and refinement of a service evaluation questionnaire. *Evaluation and Program Planning, 6*, 299-314.

Petersen, L., Jeppesen, P., Thorup, A., Abel, M.-B., Ohlenschlaeger, J., Christensen, T. O., et al. (2005). A randomised multicentre trial of integrated versus standard treatment for patients with a first episode of psychotic illness. *British Medical Journal, 331*(7517), 602.

Prieto, L., Novick, D., Sacristán, J. A., Edgell, E. T., & Alonso, J. (2003). A Rasch model analysis to test the cross-cultural validity of the EuroQoL-5D in the Schizophrenia Outpatient Health Outcomes Study. *Acta Psychiatrica Scandinavica, 107*, 24-29.

Ritsner, M., Kurs, R., Gibel, A., Ratner, Y., & Endicott, J. (2005). Validity of an abbreviated quality of life enjoyment and satisfaction questionnaire (Q-LES-Q-18) for schizophrenia, schizoaffective, and mood disorder patients. *Quality of Life Research, 14*, 1693-1703.

Rosen, K., & Garety, P. (2005). Predicting Recovery From Schizophrenia: A Retrospective Comparison of Characteristics at Onset of People With Single and Multiple Episodes. *Schizophr Bull, 31*(3), 735-750.

Tohen, M., Hennen, J., Zarate, C. M., Baldessarini, R. J., Strakowski, S. M., Stoll, A. L., et al. (2000). Two-Year syndromal and functional recovery in 219 cases of first-episode major affective disorder with psychotic features. *Americal Journal of Psychiatry, 157*, 220-228.

LEBENSLAUF

Persönliche Daten

Name	PD Dr. med. Karow
Vorname	Anne
Geburtstag/-ort	02. April 1971, Hamburg
Nationalität	Deutsch
Adresse	Schrammsweg 6, 20249 Hamburg
Familie	Lebenspartner Christoph Himmel, Sohn Leo

Schulausbildung

1977-1981	Ludwig-Frahmschule Hamburg
1981-1990	Gymnasium Harksheider Strasse, Hamburg

Studium

1991-1998	Humanmedizin, Universität Hamburg

Dissertation

Fach Orthopädie (Doktorvater: Prof. Dr. E. Hille): „Verlauf der Konzentrationen von Kobalt und Chrom im Serum bei Implantation von Hüft- und Knieendoprothesen unter Verwendung von Retransfusionssystemen“, 2001

Habilitation

Fach Psychiatrie: Gesundheitsbezogene Lebensqualität bei schweren psychischen Störungen am Beispiel von Patienten mit schizophrenen Störungen und Opioidabhängigkeit, 2009

Berufstätigkeit

1999 – 2001	Wissenschaftliche Mitarbeiterin, Zentrum für psychosoziale Medizin, Psychotherapie und Psychiatrie, Universitätsklinik Hamburg-Eppendorf Assistenzarzt in den Bereichen Psychose und Sucht
2001-2002	Psychiatric Registrar in EPPIC (Early Psychosis Prevention and Intervention Center), Mental Health Service for Kids and Youth, Melbourne, Australien
2002-2004	Wissenschaftliche Mitarbeiterin, Zentrum für Interdisziplinäre Suchtforschung der Universität Hamburg (ZIS)
2004-2005	Wissenschaftliche Mitarbeiterin, Klinik für Neurologie, Universitätsklinik Hamburg-Eppendorf
2005-2007	Wissenschaftliche Mitarbeiterin, Zentrum für psychosoziale Medizin, Psychotherapie und Psychiatrie, Universitätsklinik Hamburg-Eppendorf Assistenzarzt in den Bereichen Sucht, Angstspektrumsstörungen und Tagesklinik für Persönlichkeitsstörungen, Depression und Psychosen
Februar 2007	Facharzt für Psychiatrie und Psychotherapie
2007-2008	Mutterschutz und Elternzeit
2008-2009	Wissenschaftliche Mitarbeiterin, Zentrum für psychosoziale Medizin, Psychotherapie und Psychiatrie, Universitätsklinik Hamburg-Eppendorf
Juni 2009	Habilitation mit dem Thema: Gesundheitsbezogene Lebensqualität bei schweren psychischen Störungen am Beispiel von schizophrenen Störungen und Opioidabhängigkeit
2009-	Oberärztliche Leitung der Psychosen Ersterkennungs- und Behandlungsambulanz (PEB)

Forschungsschwerpunkte

- Untersuchung von Patienten relevanter Parameter (Patient-rated Outcomes - PRO) wie Lebensqualität, Behandlungszufriedenheit und subjektives Wohlbefinden bei schizophrenen Störungen und Suchterkrankungen unter der Berücksichtigung klinischer und anderer Einflussgrößen
- Früherkennung und Frühbehandlung von Psychosen, wissenschaftliche Untersuchung krankheitsverlaufsbeeinflussender Faktoren bei Patienten mit potentiellem Prodrom und Ersterkrankungen von Psychosen
- Gesundheitsökonomische Aspekte der Versorgung und differentielle Untersuchung verschiedener Versorgungsformen für Patienten mit Psychosen
- Untersuchung verschiedener Wirkmechanismen von Antipsychotika bei schizophrenen Störungen

Ad hoc Gutachtertätigkeit

1. Schizophrenia Research
2. Journal of Psychiatric Research
3. Psychiatry Research
4. European Psychiatry
5. Pharmacopsychiatry
6. Psychopathology
7. Progress in Neuro-Psychopharmacology & Biological Psychiatry
8. European Addiction Research
9. Suchttherapie

Mitgliedschaften

Deutsche Gesellschaft für Psychiatrie, Psychotherapie und Nervenheilkunde (DGPPN)

Deutsch-chinesische Gesellschaft für Medizin

Laufende Forschungsprojekte

Gefördert (Beginn 6/2009)	Psychoseprävention bei Personen mit erhöhtem Psychoserisiko (PREVENT Studie) (PD Dr. Andreas Bechdolf; Prof. Dr. Joachim Klosterkötter, Prof. Dr. Martin Lambert, PD Dr. Anne Karow, PRE-VENT Study group, DFG)
Gefördert (Beginn 8/2010)	Early cognitive behavioural psychotherapy in subjects at high risk for bipolar affective disorders (Acronym: EarlyCBT) (Prof. Dr. Bauer, Dr. Andrea Pfennig, Prof. Dr. Juckel, Prof. Dr. Martin Lambert, PD Dr. Andreas Bechdolf; PD Dr. Anne Karow, DFG)
Gefördert (Beginn 11/2010)	Aufklärung und Bildung über psychische Erkrankungen in der Hamburger Bevölkerung (Prof. Dr. Martin Lambert, Prof. Dr. Thomas Bock, PD Dr. Anne Karow, BMBF)
Gefördert (Beginn 11/2010)	Gesundheitsnetz Psychose: Verbesserte Früherkennung und Frühbehandlung durch Aufklärung, Fortbildung und Errichtung eines Behandlungsnetzwerkes mit Früherkennungsmandat und Integrierter Versorgung (Prof. Dr. Martin Lambert, Prof. Dr. Thomas Bock, PD Dr. Anne Karow, BMBF)

Betreuung medizinischer Dissertationen

Assistenzarzt Daniel Schnedler, Stand: abgeschlossen, Note: 2

Assistenzarzt Florian Hirdes, Stand: abgeschlossen, Note: 1

Assistenzärztin Claudia Osterwald, Stand: Korrektur abgeschlossen

Assistenzärztin Anna Rehwinkel, Stand: Korrektur abgeschlossen

Assistenzarzt Rasmus Knauer, Stand: in Arbeit

Cand. med. Florian Gau, Stand: in Arbeit

Cand. Med. Aysen Yilmaz, Stand: in Arbeit

Kongresse

Mitglied des Programmkomitees und der wissenschaftlichen Leitung des jährlich stattfindenden Fachkongresses „Die subjektive Seite der Schizophrenie“

Preis

Lilly Quality of Life Award 2011

Anlage 3 Schriftenverzeichnis der Antragsteller

Schriftenverzeichnis von PD Dr. Anne Karow

Originalarbeiten

1. Naber D. Karow A., Good tolerability equals good results: The patient's perspective, Eur. Neuropsychopharmacol 2001; (supp4): 391-6
2. Naber D. Karow A. Lambert M., Psychosocial outcomes in patients with schizophrenia: Quality of life and reintegration, Curr. Opinion in Psych. 2002; 15: 31-36
3. Haasen C. Lambert M. Yagdiran O. Karow A. Krausz M. Naber D., Comorbidity of schizophrenia and galactosemia: Effective clozapine treatment with weight gain, Int. Clin. Psychopharm. 2003 Mar; 18(2): 113-115
4. Karow A. Lambert M., Polypharmacy in treatment with psychotropic drugs: The underestimated phenomenon, Curr. Opinion in Psych. 2003; 16(6): 713-718
5. Lambert M. Schimmelmann B.G. Karow A. Naber D., Subjective Well-being and Initial Dysphoric Reaction under Antipsychotic Drugs - Concepts, Measurement and Clinical Relevance, Pharmacopsychiatry 2003 Dec; 36 (supp3): 181-190
6. Schäfer I. Lauterwein N. Karow A. Naber D., Ambulante Verordnungen atypischer Antipsychotika im Anschluss an die stationäre Behandlung schizophrener Patienten, Psychiatr Prax. 2004, 31: 74-77
7. Naber D. Lambert M. Karow A., Subjective well-being under antipsychotic treatment and its meaning for compliance and course of disease, Psychiatr. Prax. 2004 Nov; 31 (supp2): 230-232
8. Lambert M. Conus P. Eide P. Mass R. Karow A. Moritz S. Golks D. Naber D., Impact of present and past antipsychotic side effects on attitude toward typical antipsychotic treatment and adherence, Eur. Psychiatry 2004 Nov; 19(7): 415-422.
9. Reimer J. Gilg K. Karow A. Esser J. Franke GH., Health-related quality of life in blepharospasm or hemifacial spasm, Acta Neurol. Scand. 2005 Jan; 111(1): 64-70.
10. Naber D. Karow A. Lambert M., Subjective well-being under the neuroleptic treatment and its relevance for compliance, Acta Psychiatr. Scand. (supp) 2005; (427): 29-34.
11. Schimmelmann B.G. Moritz S. Karow A. Schäfer I. Bussopulos A. Golks D. Krausz M. Naber D. Lambert M., Correlates of subjective well-being in schizophrenic patients treated with atypical antipsychotics, Int. J. Psych. Clin. Prac. 2005; 9(2): 94-98
12. Karow A. Moritz S. Lambert M. Schoder S. Krausz M., PANSS syndromes and quality of life in schizophrenia, Psychopathology 2005 Nov-Dec; 38(6): 320-326.
13. Moritz S. Rufer M. Fricke S. Karow A. Morfeld M. Jelinek L. Jacobsen D., Quality of life in obsessive-compulsive disorder before and after treatment, Compr. Psychiatry 2005 Nov-Dec; 46(6): 453-459
14. Karow A. Schnedler D. Naber D., What would the patient choose? - Subjective comparison of atypical and typical neuroleptics, Pharmacopsychiatry 2006 Mar; 39(2): 47-51.
15. Reimer J. Voigtlaender-Fleiss A. Karow A. Bornfeld N. Esser J. Franke G.H., The impact of diagnosis and plaque radiotherapy treatment of malignant choroidal melanoma on patients' quality of life, Psychooncology 2006 Apr 24

16. Karow A. Pajonk F.G., Insight and quality of life in schizophrenia: Recent findings and treatment implications, Curr. Opin. Psychiatry. 2006 Nov; 19(6): 637-641.

17. Lambert M. Schimmelmann B.G. Naber D. Schacht A. Karow A. Wagner T. Czekalla J. Clin J., Prediction of remission as a combination of symptomatic and functional remission and adequate subjective well-being in 2,960 patients with schizophrenia, Psychiatry 2006 Nov; 67(11): 1690-1697.

18. Karow A. Czekalla J. Dittmann R.W. Schacht A. Wagner T. Lambert M. Schimmelmann B.G. Naber D. Clin J., Association of subjective well-being, symptoms and side effects with compliance after 12 month treatment in Schizophrenia, Psychiatry 2007 Jan; 68(1): 75-80.

19. Karow A. Pajonk F. Reimer J. Hirdes F. Osterwald C. Naber D. Moritz S., The dilemma of insight into illness in schizophrenia: Self- and expert-rated insight and quality of life, Eur. Arch. Psychiatry Clin. Neurosci. 2007 Nov; 13: 768-775

20. Schäfer I. Reininghaus U. Langeland W. Voss A. Zieger N. Haasen C. Karow A., Dissociative symptoms in alcohol-dependent patients: Associations with childhood trauma and substance abuse characteristics, Comprehensive Psychiatry 2007 Nov-Dec; 48(6): 539-545

21. Karow A. Verthein U. Krausz M. Schäfer I., The association of personality disorders, family conflicts and treatment with quality of life in opiate addiction, Eur. Add. Res. 2008; 14: 38-46

22. Karow A. Schäfer I. Hirdes F. Osterwald C. Naber D., Positivsymptome und Lebensqualität aus der Sicht schizophrener Patienten, Psych. Prax. 2008; Mai; 27

23. Schäfer I. Gschwend C. Karow A. Naber D., Attitudes of Patients with Schizophrenia to Psychiatric Research, Int. J. Psych. Clin. Pract. 2008; 12(3): 165-170

24. Lambert M. Naber D. Schacht A. Wagner TT. Hundemer H.-P. Karow A. Huber C.G. Suarez D. Haro JM. Novick D. Schimmelmann B.G., Rates and predictors of remission and recovery during three years in 392 never-treated patients with schizophrenia, Acta Psych. Scand. 2008; 118: 220–229

25. Lambert M. Huber C.G. Naber D. Schacht A. Wagner TT. Hundemer H.-P. Karow A. Schimmelmann B.G.,Treatment of severe agitation with olanzapine in 166 patients with schizophrenia, schizoaffective or bipolar I disorder, Pharmacopsychiatry. 2008 Sep;41(5):182-9.

26. Lambert M. Schimmelmann BG. Schacht A. Karow A. Wagner T. Wehmeier PM. Huber CG. Hundemer HP. Dittmann RW. Naber D., Long-term patterns of subjective wellbeing in schizophrenia: cluster, predictors of cluster affiliation, and their relation to recovery criteria in 2842 patients followed over 3 years Schizophr Res. 2009 Feb;107(2-3):165-72.

27. Lambert M. Schimmelmann B.G. Naber D. Eich F.-X. Schulz H. Huber C.G. Karow A., Early- and delayed antipsychotic response and prediction of outcome in 528 severely impaired patients with schizophrenia treated with amisulpride, Pharmacopsychiatry, 2009 42(6): 277-83

28. Lambert M. Naber D. Karow A. Huber C. Schimmelmann B., Subjective Wellbeing under quetiapine treatment: Effect of diagnosis, mood state, and anxiety, Schizophr. Res. 2009, 110(1-3): 72-9

29. Moritz S. Peters M.J.V. Karow A. Deljkovic A. Naber D., Cure and/or curse? Ambivalent attitudes towards neuroleptic medication in schizophrenia and non Schizophrenia patients, Mental Illness, 2009, 1:e2

30. Moritz S., Veckenstedt, R., Randjbar S., Vitzthum F., Karow A., Lincoln, T.M., Course and determinants of self-esteem in people diagnosed with schizophrenia during psychiatric treatment, Psychosis. 2010 1

31. Eiroá-Orosa FJ. Verthein U. Kuhn S. Lindemann C. Karow A. Haasen C. Reimer J. Implication of gender differences in heroin-assisted treatment: results from the German randomized controlled trial. Am J Addict. 2010 Jul-Aug;19(4):312-8.

32. Karow A. Reimer J. Schäfer I. Krausz M. Haasen C. Verthein U., Quality of life under maintenance treatment with heroin versus methadone in patients with opioid dependence, Drug Alcohol Depend. 2010 Dec 1;112(3):209-15.

33. Karow A. Verthein U. Pukrop R. Reimer J. Haasen C. Krausz M. Schäfer I., Quality of life profiles and changes in the course of maintenance treatment among 1,015 patients with severe opioid dependence, Substance Use Misuse, 2011;46(6):705-15.

34. Schimmelmann BG. Conus P. Cotton SM. Kupferschmid S. Karow A. Schultze-Lutter F. McGorry PD. Lambert M. Cannabis use disorder and age at onset of psychosis - A study in first-episode patients. Schizophr Res. 2011 Jun;129(1):52-6. Epub 2011 Apr 17.

35. Karow A. Moritz S. Lambert M. Schöttle D. Naber D., Remitted but still impaired? Symptomatic versus functional remission in patients with schizophrenia, Eur Psychiatry. 2011 May 12.

36. Karow A. Lambert M. Naber D. Moritz S., Remission as perceived by people with schizophrenia, family members and psychiatrists, Eur Psychiatry. 2011 May 13

37. Reimer J. Verthein U. Karow A. Schäfer I. Naber D. Haasen C. Physical and mental health in severe opioid-dependent patients within a randomised controlled maintenance treatment trial. Addiction. 2011 Apr 14. doi: 10.1111/j.1360-0443.2011.03463.x.

38. Karow A. Reimer J. König HH. Heider D. Bock T. Huber CG. Schöttle D. Meister K. Rietschel L. Ohm G. Schulz H. Naber D. Schimmelmann BG. Lambert M. Cost-effectiveness of 12-month therapeutic Assertive Community Treatment as part of Integrated Care vs. Standard Care in patients with schizophrenia treated with Quetiapine IR (ACCESS Trial). J. Clin. Psych. In press

39. Karow A. Schimmelmann B.G. Kuhnigk O. Schöttle D. Rehwinkel A. Naber D. Lambert M. Self- and expert - rated insight in patients with schizophrenia and short-term outcome, Psychopathology, submitted

Reviews und Übersichtsarbeiten:

1. Karow A. Naber D., Internationale Standards in der medikamentösen Therapie – Auswirkungen auf die Langzeitbehandlung, Hrsg.: Ehlers A.P.F. Deutsch E. Schwandt P. Wille E. Feldmann M. Stoschek J., Recht und Politik im Gesundheitswesen pmi Verlag AG 2001; Band 7: 9-13

2. Karow A., Die subjektive Verarbeitung neuroleptischer Behandlung, Hrsg.: Jost, Neuro Date aktuell, W.H. Westermayer Verlag AG 2002

3. Karow A. Naber D., Subjective well-being and quality of life under atypical antipsychotic treatment, Psychopharmacology 2002, 162: 3-10

4. Karow A. Schnedler D. Naber D., Wie entscheiden sich Patienten?, Neuro-Psychiatrische Nachrichten, Biermann Verlag 2002

5. van Sung T. Baehr L. Karow A. Krausz M., HEANTOS – Medikamenten-entwicklung auf der Basis traditioneller Vietnamesischer Medizin in der Behandlung von Drogenabhängigkeit, Suchttherapie 2003; 2: 93-98

6. Karow A., Die subjektive Verarbeitung neuroleptischer Behandlung, Info. Neuro. 2003; 21-26

7. Aderhold V. Lambert M. Bock T. Karow A. Schimmelmann B.G., Psychosenbehandlung verbessern - ein Projekt für ersterkrankte psychotische Patienten an der Universitätsklinik Hamburg-Eppendorf, Soziale Psychiatrie 2004, 28(1): 12-14

8. Karow A. Naber D., Therapie orientiert am subjektiven Wohlbefinden statt Symptomreduktion „um jeden Preis“– Paradigmenwechsel in der Therapie mit Antipsychotika, MMW Fortschr. Med. 2005 Sep 15; 147(37): 44-45.

9. Karow A. Naber D., Entdecken Sie die " subjektive Seite". Befindlichkeits-orientierte Therapie mit Antipsychotika, MMW Fortschr. Med. 2005; 25: 1-2

10. Lambert M. Karow A. Naber D., Lebensqualität bei schizophrenen Patienten unter besonderer Berücksichtigung von Quetiapin, Hrsg.: Möller, Pharmakologie, Indikationen, therapeutische Erfahrungen; Das Quetiapin-Dossier 2005; 245-259

11. Meyer T. Karow A. Naber D. Franz M., Anmerkungen zur kritischen Betrachtung eines modernen Konstrukts - Subjektive Lebensqualität, Nervenarzt 2005 Mai; 76(5): 623-624

12. Karow A., Remission und Lebensqualität als Erfolgsparameter der Behandlung, Psycho. Neuro. 2006; 32(7+8): 231-236

13. Karow A. Naber D., Compliance sichern als gemeinsamen Prozess, Der Neurologe und Psychiater 2007; 03: 42-46

14. Lambert M. Karow A. Leucht S. Schimmelmann BG. Naber D. Remission in schizophrenia: validity, frequency, predictors, and patients' perspective 5 years later. Dialogues Clin Neurosci. 2010;12(3):393-407. Review.

Buchbeiträge:

1. Karow A. Moritz S. Lambert M. Krausz M. Naber D., Lebensqualität bei schizophrenen Patienten unter der Behandlung mit atypischen Neuroleptika, Hrsg.: Bullinger M., Siegrist J., Ravens-Sieberer U., Jahrbuch für Psychiatrie und Psychotherapie; Hogrefe Verlag 2000; 74-85

15. Karow A. Naber D., Lebensqualität bei psychischen Störungen, In: Lebensqualität und Gesundheitsökonomie in der Medizin, Hrsg.: Ravens-Sieberer, U., Cieza, A., Eco-med Verlag 2000; 199-212

16. Karow A. Perro C. Yagdiran O. Moritz S. Nika E. Basdekis R. Lambert M. Briken P. Gottwalz E. Jung J. Krausz M., PERSIST-Personenzentrierte Settingübergreifende Integrative Schizophrenietherapie, Leponex – Pharmakologie und Klinik eines atypischen Neuroleptikums; Springer-Verlag 2000; 84-91

17. Moritz S. Naber D. Lausen A. Küppers D. Lambert M. Karow A. Krausz M., Primäre und sekundäre Konsequenzen typischer und atypischer Neuroleptika auf das subjektive kognitive Leistungsvermögen, Leponex – Pharmakologie und Klinik eines atypischen Neuroleptikums; Springer Verlag 2000; 91-96

18. Karow A., Lebensqualität schizophrener Patienten unter der Therapie mit atypischen Neuroleptika, Hrsg.: Krausz, Lambert, Naber, Atypische Neuroleptika in der Behandlung schizophrener Patienten; Uni-med Verlag 2001; 225-233

19. Karow A. Lambert M. Naber D., Lebensqualität schizophrener Patienten, Hrsg.: Schmauss M., Monographie Schizophrenie – Pathogenese, Diagnostik und Therapie; Uni-med Verlag 2003; 154-170

20. Karow A, Haasen C., Substitution mit Heroin, Hrsg.: Krausz M. Haasen C. Naber D., Pharmakotherapie der Sucht, 3.1.3: 124-134

21. Schäfer I. Karow A. Gottwalz E., Qualitätssicherung in der Versorgung schizophrener Patienten, Hrsg.: Naber, Hohagen, Schizophrenie; Thieme-Verlag 2003; 183-197

22. Haasen C. Reimer J. Karow A., Behandlungsansätze – Substitution, Kompendium Sucht; Thieme-Verlag 2003; 82-105

23. Karow A. Schmudlach S. Penss S. Weber J. Becker A. Bollow M. Kemme G., Die subjektive Seite des Patienten – wie wichtig ist Rückfallprophylaxe, Hrsg.: Pajonk F.-G. Falkai P., Langzeittherapie der Schizophrenie; Uni-Med Verlag 2005; 144-156

24. Moritz S. Karow A. Lambert M. Naber D., Neurokognition unter konventionellen und atypischen Neuroleptika: Eine methodenkritische Übersicht, Hrsg.: Lasar M. Goldbeck F., Kognition und Schizophrenie - Biopsychosoziale Konzepte; Lengerich Pabst 2004; 67-94

25. Krausz M. Karow A., Suicide among schizophrenic adolescents in the long term of the illness, Hrsg.: Tatarelli R. Pompili M. Girardi P., Suicide in Schizophrenia; Nova Science Publishers Inc. New York 2006; ChXV: 215-225

26. Karow A. Lambert M. Naber D., Lebensqualitätsforschung, Hrsg.: Holsboer Gründer Benkert, Handbuch der Psychopharmakotherapie; Springer-Verlag 2007; 489-494

27. Naber D., Bullinger M., Karow A., Psychopharmaka und Lebensqualität, Hrsg.: Riederer, Laux, Neuropsychopharmaka 2. Auflage, Band 1, 2010

Curriculum vitae

**Name**	Daniel Georg Schöttle
**Nationality**	German
**Date and Place of Birth**	22.04.1977 in Pforzheim, Germany
**Private address**	Hospitalstrasse 62, 22767 Hamburg, Germany
**Marital Status**	not married, in partnership,1 child
**School**	1983-85: Primary school, Illingen<br>1985-1987: Primary school, Donzdorf<br>1987-1996: Grammar school, Donzdorf
**Civil Service**	1996-1997: Hospital „Klinik am Eichert“, Surgery
**Study**	2003: University of Ulm<br>2003-2004: University “Autonoma”, Madrid, Spain<br>2003-2004: University of Dresden
**Graduation**	2004, Carl-Gustav-Carus-University, Medical School, Dresden
**Occupation**	
01.01.2005 – 16.10.2005	Klinikum Chemnitz gGmbh, Psychiatric Hospital<br>General Psychiatry and special ward for affective disorders, Prof. Bräunig
since 17.10.2005	University Hospital Hamburg, Psychiatry, Prof. Naber<br>Special Outpatient center for people with schizophrenia and bipolar disorders<br>Psychosis Early Detection and Intervention Centre (PEDIC)<br>Consultation-hours: Bipolar-Disorder Outpatient-Unit, Consultation-hour for people with adult ADHD,<br>Member oft the Assertive Community Treatment Team “Integrated Care”
1.4.2009-1.4.2010:	University Hospital, Neurology, Prof. Gerloff
since 1.4.2010	Special Outpatient center for people with schizophrenia and bipolar disorders

Research Activity

2001-2004: Dissertation, Research Assistant in University of Ulm, Neurochemical Laboratory, Prof. Tumani, CSF-Dementia-Research-Group

2005-2008: Research Assistant in the "BiPo-Project" – Psychotherapy versus Psychoeducation in the long-term follow up of patients with bipolar disorders"

2006-2007: Research Assistant in Study: "Compliance and disengagement in first-episode psychosis: a study evaluating case management and acute home treatment care"

2007-2009: Research Assistant in Study "Compliance and disengagement in patients with schizophrenia and bipolar disorder comparing case management and acute home treatment care, the "integrated care program"

2008: Research Assistant in Study "MDQ/HCL32, diagnostic validity and reliability in bipolar patients"

2008: Principal Investigator Study "Changes in sexual behaviour in patients after manifestation of first-episode psychosis"

2008: Research Assistant Study "Bipolar Disorder: influence of family therapy on burden of disorder and outcome of therapy"

2009: Principal Investigator in Study "HIV and Hepatitis prevalence, HIV knowledge and risk behaviour in patients with schizophrenia, bipolar disorder and addiction disorders"

Thesis

2008, Title: Differentiation of demential disorders with a combination of neurobiological parameters in human cerebrospinal fluid

Publications

Schöttle, D., Rode, S., Kruger, S, Braunig, P.
Self-rating scales for manic episodes
Psychiatr Prax. 2006 Mar;33 Suppl 1:S55-9. Review. German.

Brettschneider J, Petzold A, **Schöttle D**, Claus A, Riepe M, Tumani H.
The neurofilament heavy chain (NfH) in the cerebrospinal fluid diagnosis of Alzheimer's disease.
Dement Geriatr Cogn Disord. 2006;21(5-6):291-5. Epub 2006 Feb 10.

Burlon, M, Naber, **D, Schöttle**, D, Lambert, M (2008) Nebenwirkungen und Therapie-Adhärenz in der Behandlung der Schizophrenie. Der Neurologe und Psychiater, 27-33.

Kammerahl D, **Schöttle D**, Huber CG.
Sexualität und Schizophrenie – eine Übersicht.
Z Sexualforschung 2009

Schöttle D, Kammerahl D, Huber J, Briken P, Lambert M, Huber CG.
Sexual Problems in Patients with Schizophrenia.
Psychiatr Prax. 2009 May;36(4):160-168. Epub 2009 May 7.

Lambert M, Bock T, **Schöttle D** et al. Assertive Community Treatment (ACT) as part of Integrated Care versus Standard Care: a 12-month trial in patients with first- and negatively selected multiple-episode schizophrenia-spectrum disorders treated with quetiapine IR. J Clin Psychiatry, J Clin Psychiatry. 2010 Oct;71(10):1313-23. Epub 2010 Mar 23.

Thomas D. Meyer, Britta Bernhard, Christoph Born, Kristina Fuhr, Sonja Gerber, Lars Schaerer, Jens M. Langosch, Andrea Pfennig, Johanna Sasse, Susan Scheiter, **Daniel Schöttle**, Dietrich van Calker, Larissa Wolkenstein & Michael Bauer
The Hypomania Checklist – 32 and the Mood Disorder Questionnaire as screening tools – going beyond samples of purely mood disordered patients, submitted, J Affec Dis 2010

Daniel Schöttle, Martin Lambert, Christian G. Huber, Peer Briken, Psychopharmaka und sexuelle Störungen
Psychiatrie und Psychotherapie up2date 3 ê2009 êDOI 10.1055/s−0029−1220347

Anne Karow, J. Reimer, H.H. König, Thomas Bock, **Daniel Schöttle**, MD1; Klara Meister, PhD1; Liz Rietschel, Gunda Ohm, Holger Schulz, Benno Schimmelmann, Dieter Naber, Martin Lambert Cost-utility analysis of 12 months Assertive Community Treatment as part of Integrated Care versus Standard Care in patients with schizophrenia treated with Quetiapine (ACCESS Trial), submitted

Anne Karow, MD; Benno G. Schimmelmann, MD; Ingo Schäfer, MD, Olaf Kuhnigk, MD; **Daniel Schöttle**, MD, Anna Rehwinkel, Dieter Naber; MD, Martin Lambert, MD, Course and clinical correlates of self- and expert-rated insight in patients with acute schizophrenia, submitted

Karow A, Moritz S, Lambert M, **Schöttle D**, Naber D; on behalf of the EGOFORS initiative.
Remitted but still impaired? Symptomatic versus functional remission in patients with schizophrenia. Eur Psychiatry. 2011 May 12. [Epub ahead of print]

Huber CG, **Schöttle D**, Lambert M, Moritz S, Naber D, Schroeder K.
Applicability of the Clinical Global Impressions-Aggression (CGI-A) scale for use in file audit trials.

Pharmacopsychiatry. 2011 Jul;44(5):189-92. Epub 2011 Jul 12.

Schöttle D, Huber CG, Bock T, Meyer TD.
Psychotherapy for bipolar disorder: a review of the most recent studies.
Curr Opin Psychiatry. 2011 Nov;24(6):549-55. Review.

Huber CG, **Schöttle D**, Lambert M, Hottenrott B, Agorastos A, Naber D, Schroeder K.
Brief Psychiatric Rating Scale - Excited Component (BPRS-EC) and neuropsychological dysfunction predict aggression, suicidality, and involuntary treatment in first-episode psychosis.
Schizophr Res. 2012 Feb;134(2-3):273-8. Epub 2012 Jan 4.

Abstracts

Claus, A., **Schottle, D.**, Riepe, M., Tumani, H.
Diagnostische Wertigkeit von hirnspezifischen Proteinen im Liquor bei der Klassifikation dementieller Erkrankungen (Kongress der Deutschen Gesellschaft für Neurologie – DGN – Hamburg, 2003)

A Claus, **D Schöttle**, U Beinhoff, M Riepe, H Tumani (Ulm)
Diagnostische Wertigkeit der Analyse von hirnspezifischen Proteinen im Liquor bei der Differenzierung organisch und nicht organisch bedingter dementieller Erkrankungen
Akt Neurol 2004; 31

Claus, A., **Schottle, D.**, Riepe, M., Tumani, H.
Diagnostische Wertigkeit der Analyse von hirnspezifischen Proteinen im Liquor bei der Differenzierung organisch und nicht organisch bedingter dementieller Erkrankungen (Kongress der Deutschen Gesellschaft für Neurologie – DGN – Düsseldorf, 2004)

Burlon, M, **Schöttle, D.**, Bock, T., Lambert, M.
Erste Inanspruchnahmeergebnisse einer neuen interaktiven und trialogischen Website zu Psychosen und Bipolaren Erkrankungen: www.psychose.de
DGPPN 2008, Berlin

Martin Lambert, Michael Sadre Chirazi-Stark, **Daniel Schöttle**, Michael Schödlbauer, Marietta Frieling. Manoshi Pakrasi, Dietmar Golks, Klara Meister, Dieter Naber
Assertive Community Treatment (ACT): Vorläufige Ergebnisse einer offenen, nicht-randomisierten 12-Monatsstudie bei Patienten mit einer ersten oder wiederholten Erkrankung aus dem schizophrenen Formenkreis behandelt mit Quetiapin IR.
DGPPN, Berlin 2008

Veränderung der therapeutischen Beziehung
durch Home-Treatment
Michael Schödlbauer, **Daniel Schöttle**, Martin Lambert (Hamburg)
XI. Tagung
Die subjektive Seite der Schizophrenie
Therapeutische und andere Beziehungen
Hamburg, 25. Februar bis 27. Februar 2009

Paarbeziehung und Sexualität
Daniel Schöttle, Dürten Kammerahl, Peer Briken
XI. Tagung
Die subjektive Seite der Schizophrenie
Therapeutische und andere Beziehungen
Hamburg, 25. Februar bis 27. Februar 2009

Daniel Schöttle, Martin Lambert
Management of incomplete remission and treatment resistance in first episode psychosis

7th International Meeting on the Early Phases of Mental Illnesses, Santander, Spain, 2009

Schöttle D, MD; Karow A, MD, Schimmelmann BG, MD, Conus P, MD; Cotton S, PhD; McGorry PD, MD; Huber CG, MD; Lambert M, MD
Distinction between schizoaffective and bipolar I disorder in the early phase of psychotic disorders,
IEPA, Amsterdam 2010

CG Huber,* **D Schöttle**, M Lambert, B Hottenrott, D Naber, K Schroeder
Psychopathology and neuropsychological dysfunction are differentially
connected with aggression, suicidality, and involuntary treatment in fi rst-episode psychosis
IEPA, Amsterdam 2010

Anne Karow, Benno G Schimmelmann, **Daniel Schöttle**, Christian Huber, Klara Meister, Liz Rietschel, Dietmar Golks, Thomas Bock, Dieter Naber, Martin Lambert
Clinical outcome of fi rst versus multiple-episode patients with schizophrenia spectrum disorders in 12-months Assertive Community Treatment (ACT) versus standard care
IEPA, Amsterdam 2010

Christian G. Huber, **Daniel Schöttle**, Martin Lambert, Birgit Hottenrott, Dieter Naber, Katrin Schröder
Psychopathology and neuropsychological dysfunction are differentially connected with aggression, suicidality, and involuntary treatment in first-episode psychosis
DGPPN, Berlin 2010

Daniel Schöttle
Therapie von Depressionen bei
neurologischen Erkrankungen
Neurologie Update, Hamburg, 9. Januar 2010

Daniel Schöttle
Umgang mit Individualität – Erfahrungen aus dem Hamburger Modell
XIII. Tagung
Die subjektive Seite der Schizophrenie
Bedu□rfnisorientierte Behandlung – Integration
der Versorgung
Hamburg, 23. Februar bis 25. Februar 2011

Christos Ganos, Odette Schunke, Simone Zittel, **Daniel Schöttle**, Nicole David, Andreas K Engel and Alexander Mu□nchau
High Functioning Autism and Asperger's Syndrome: The way they move - a video-based clinical study
15th international congress of Parkinsons' desease and Movement Disorders, 5.-9. Juni 2011, Toronto, Canada

C. Ganos, O. Schunke, S. Zittel, **D. Schöttle**, N. David, S. Kuehn, A. Engel, C. Gerloff, A. Münchau
High Functioning Autism and Asperger's Syndrom: a video-based evaluation study of the motor phenotype of the two syndromes
84. Kongress der Deutschen Gesellschaft für Neurologie mit Fortbildungsakademie
28.09.-01.10., Wiesbaden, Poster

Memberships

German Society of Psychiatry, Psychotherapy and Neurology (Deutsche Gesellschaft für Psychiatrie, Psychotherapie und Nervenheilkunde)

German Society for Bipolar Disorders (Deutsche Gesellschaft für Bipolare Störungen, DGBS), Study group: „DGBS, young scientists" and
Study group: „DGBS, gender specific studies in bipolar disorder" (Arbeitskreis Geschlechterspezifische Fragestellungen bei Bipolaren Störungen")

German Society for sexuality research (Deutsche Gesellschaft für Sexualforschung)

Languages English, Spanish, Latin

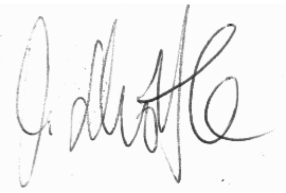

Daniel Schöttle
Hamburg, 31.01.2012

Universitätsklinikum Hamburg-Eppendorf

Universitätskrankenhaus Hamburg-Eppendorf (UKE)

Klinik und Poliklinik für Psychiatrie und Psychotherapie

Integrierte Versorgung von Patienten mit psychotischen Störungen

Patientenname:		Datum:			
Patientenvorname:		Geburtsdatum:			
			Tag	Monat	Jahr

Patienteninformation

zur Teilnahme an der Integrierten Versorgung von Patienten mit einer psychotischen Störung am UKE

Sehr geehrte Patientin, sehr geehrter Patient,

bei Ihnen wurde eine psychotische Störung diagnostiziert. Speziell für dieses Krankheitsbild haben wir ein umfassendes Behandlungsprogramm zur Durchführung der integrierten Versorgung entwickelt. Dies bedeutet, dass die für Ihre Erkrankung notwendigen Behandlungseinheiten eng aufeinander abgestimmt sind. Im Rahmen dieser Integrierten Versorgung können Sie alle Leistungen des Psychosezentrums des UKE in Anspruch nehmen. Dies beinhaltet, dass Sie einen Therapeuten und einen Arzt zur Seite gestellt bekommen, die mit Ihnen Ihre integrierte Therapie abstimmen. Damit soll die Qualität Ihrer Behandlung und damit Ihre Lebensqualität verbessert werden. Ziel ist es auch, dass Sie, wenn immer möglich, in Ihrem eigenen Umfeld behandelt und Krankenhausaufenthalte auf das Notwendigste reduziert werden. Alle Behandlungsschritte sind qualitätsgesichert und werden durch die enge Zusammenarbeit zwischen Ihrem niedergelassenen Nervenarzt/Psychiater und dem UKE zu Ihrem Wohle geregelt. Dadurch werden unnötige Behandlungsschritte vermieden und Abläufe verkürzt.

Ihre Krankenkasse hat hierzu mit dem UKE und verschiedenen niedergelassenen Nervenärzten einen Vertrag geschlossen. Das UKE koordiniert Ihre Behandlungseinheiten und regelt die Abrechnung der erbrachten Leistungen mit Ihrer Krankenkasse.

Im Rahmen dieses Vertrages erstattet Ihnen die DAK 50 % der Zuzahlung zur stationären Behandlung bis maximal 140,- € je Kalenderjahr. Voraussetzung ist, dass die stationäre Behandlung aufgrund der psychotischen Störung erforderlich ist. Bitte reichen Sie für die Erstattung ihre Zuzahlungsquittung ein.

Ihre Teilnahme an der integrierten Versorgung ist freiwillig. Um die Kommunikation mit Ihrem niedergelassenen Nervenarzt/Psychiater zu verbessern und unnötige Untersuchungen zu vermeiden, bitten wir Sie um Ihre Einwilligung, die in diesem Zusammenhang von uns über Ihre Behandlung erstellte Dokumentation Ihrem niedergelassenen Nervenarzt/Psychiater zur Verfügung stellen zu können. Gleichfalls bitten wir Sie um Ihre Einwilligung, dass auch Ihr niedergelassener Nervenarzt/Psychiater uns seine Behandlungsdokumentation zur Verfügung stellen darf.

Ein weiterer wesentlicher Bestandteil der integrierten Versorgung ist die Qualitätssicherung. Dafür werden bestimmte Angaben von Ihnen über Ihre Behandlung vom UKE ausgewertet. Die Ergebnisse der Auswertung werden Dritten ausschließlich anonymisiert zur Verfügung gestellt.

Weil uns Ihre Meinung als Patientin/Patient und die Meinung Ihrer Sie bei der Behandlung begleitenden Angehörigen wichtig ist und uns hilft, unser Behandlungsangebot weiter zu verbessern, bitten wir Sie und Ihre Angehörigen im Anschluß an Ihre Behandlung, Fragen über die erfolgte Behandlung zu beantworten.

Falls Sie Fragen oder Verständnisprobleme haben, wenden Sie sich bitte an einen Mitarbeiter des UKE oder an Ihren niedergelassenen Nervenarzt/Psychiater.

Ihre Ärzte und Ärztinnen des Universitätsklinikums Hamburg-Eppendorf

Einwilligungserklärung

zur Teilnahme an der Integrierten Versorgung von Patienten mit einer psychotischen Störung am UKE

Vor- und Nachname der Patientin/des Patienten: ____

Geburtsdatum: ___________________

Anschrift: ____

Ich möchte am Behandlungsprogramm Integrierte Versorgung teilnehmen. Mir ist bekannt, dass die Teilnahme am Programm freiwillig ist und ich die integrierte Versorgung jederzeit abbrechen darf.

Die Behandlung aufgrund meiner psychotischen Störung findet im Rahmen der Integrierten Versorgung ausschließlich im UKE und bei den niedergelassenen kooperierenden Psychiatern und Nervenärzten statt. Sofern ich in einem anderen Krankenhaus aufgenommen werde, ermächtige ich die DAK, das UKE darüber zu informieren.

Ich erkläre mich damit einverstanden, dass die mich im UKE behandelnden Ärzte bei den anderen an meiner integrierten Versorgung Beteiligten die im Zusammenhang mit der integrierten Versorgung erhobenen Behandlungsdaten und Befunde für meine konkret anstehende Behandlung abfordern. Dies gilt einschließlich der für die vorgeschriebene Qualitätssicherung erforderlichen, ggf. bei meiner Krankenkasse von anderen Leistungserbringern vorliegenden Daten.

Des weiteren bin ich damit einverstanden, dass das UKE den anderen an meiner integrierten Versorgung beteiligten Leistungserbringern die im Zusammenhang mit der integrierten Versorgung im UKE erhobenen Behandlungsdaten für meine konkret anstehende Behandlung zur Verfügung stellt.

Ort / Datum: ___________________________ ___________________________________
(Unterschrift der Patientin/des Patienten)

____
(Bei Patienten, für die eine gesetzliche Betreuung eingerichtet wurde, welche die Gesundheitsfürsorge umfasst, - zusätzliche - Unterschrift des Betreuers/der Betreuerin)
