# Supplementary material for: Effectiveness of integrated care including therapeutic assertive community treatment in severe schizophrenia-spectrum and bipolar I disorders: Four-year follow-up of the ACCESS II study
Source: PLoS One. 2018 Feb 27;13(2):e0192929. doi: 10.1371/journal.pone.0192929 (PMC5828355; doi:10.1371/journal.pone.0192929)
Supplement: S4 File — (DOCX) [file pone.0192929.s004.docx]

**2.2.3 Integrated Care Models for Psychosis: The Hamburg Model**

The “Hamburg Model” designates an integrated care model for severely ill patients with psychotic disorders according to §140 SGB V (German Social Code, Book V). The model includes a cross-sectoral and long-term treatment within a network consisting of the Psychosis Center of the University Medical Center Hamburg-Eppendorf (UKE) and private psychiatrists (table 1). The model has been implemented since 1.05.2007. The following german health insurance funds participate: DAK, HEK, IKK Classic and AOK Rheinland Hamburg. There are health insurance-specific and diagnostic indications for participation as well as indications representing a high severity of the disease. Assertive Community Treatment (ACT) is the core element of the treatment model. In addition, participants can use all offers of the Psychosis Center and involved private psychiatrists. The Hamburg Model is a so-called “Capitation Model” meaning that the UKE as the main contractor receives an insurant-related annual lump sum, with which all services are financed.

**Table 1: Overview of the Hamburg Model**

| **Information** | **Details** |
| --- | --- |
| **Start** | - 1.05.2007 (duration: 4 years, 7 months) |
| **Participating health insurance funds** | - DAK, HEK, IKK Classic, AOK Rheinland Hamburg |
| **Indication** | - Psychotic Disorder (12 diagnoses) - Hospitalization - High general severity of the disease - Presence of an associated syndrome with high severity |
| **Financing** | - Managed Care Capitation Model (annual lump sum) - Annual lump sum starts on the day of hospital admission - Annual lump sums for the first two years, then stepped lump sums depending on severity of the disease (CGI-S) and functioning level (GAF) = Remission over the last six months - Every year new rating with regard to remission |

**2.2.4 Indications for Participation in the Hamburg Model (in- and exclusion criteria)**

a) Insured with one of the following health insurance funds:

DAK, HEK, IKK Classic and AOK Rheinland Hamburg

b) Fulfilment of one of the following main diagnoses according to ICD-10:

| F1 Disorders: | Substance induced psychotic disorder (F1x.5) |
| --- | --- |
| F2 Disorder: | Schizophrenia (F20)  Persistent delusional disorder (F22)  Acute and transient psychotic disorders (F23)  Induced delusional disorder (F24)  Schizoaffective disorders (F25)  Other nonorganic psychotic disorders (F28)  Unspecified nonorganic psychosis (F29) |
| F3 Disorders: | Manic episode with psychotic symptoms (F30.2)  Bipolar affective disorder (F31)  Severe depressive episode with psychotic symptoms (F32.3)  Recurrent depressive disorder, current episode severe with psychotic symptoms (F33.3) |

c) Fulfilment of at least one of the following severity criteria:

| Present hospitalization | The patient is admitted to hospital by a private psychiatrist or in case of emergency by a hospital doctor. The severity of the disease usually makes an in-patient care necessary. |
| --- | --- |
| Fulfilment of the general severity criterion | The patient fulfils a general severity with a total score of at least 40 points as assessed with the Brief Psychiatric Rating Scale (BPRS). Criterion of chronicity (many in-patient stays in the last 2 years) |
| Fulfilment of at least one of the specific severity criteria | Hallucinations (item 10) ≥ 6 points  Delusion (item 11) ≥ 6 points  Disorganization (item 15) ≥ 6 points  Depressive-suicidal syndrome ≥ 10 points  Suicidal syndrome ≥ 6 points  Manic syndrome ≥ 15 points  Disruptive behavior syndrome in the context of a psychosis ≥ 15 points  Syndrome of prevalent negative symptoms ≥ 15 points |
| Exclusion criteria | Psychotic disorders due to a medical condition  IQ< 70 |

**2.2.5 The Treatment Model**

The model includes a cross-sectoral and long-term treatment within a network consisting of the Psychosis Center of the UKE and private psychiatrists. Figure 2 shows all institutions involved as well as the 20 psychiatrists who are currently participating.

The Psychosis Center of the UKE comprises several sub-areas, which are involved in the care of psychotic patients, resp. which are available for the patients and their families within the framework of integrated care. The health care facilities involved include:

- Specialised Psychosis Outpatient Center (SPA) of the UKE with a long-term outpatient individual and group offer
- The crisis day-clinic for young adults with psychosis (age range of 16-29, 8-10 places)
- The Assertive Community Treatment (ACT) Team
- The psychosis inpatient unit of the adult psychiatry (PS 2, 23 beds)
- The acute inpatient unit of the adult psychiatry (PS EG, 23 beds)
- 20 private psychiatrists from the extended sector (esp. Eimsbüttel)

Assertive Community Treatment (ACT) is the core element of the treatment model. ACT is one of the evidence-based treatment models for patients with severe mental illness and comprises an intensive and long-term treatment for people with chronic and severe mental illness (Marshall & Lockwood 2011). The development of a team is carried out according to the guidelines of the Assertive Community Treatment Association (ACTA). The quality of the team is ensured based on 28 criteria of the Dartmouth Assertive Community Treatment Scale (DACTS) (range from 1 = “poor fidelity” to 5 = “excellent fidelity”; Teague et al. 1998). The general quality criteria for a team are as follows:

- Multiprofessional team
- Small provider/consumer ratio of 1:10 to max. 1:20
- No-drop-out policy
- 24 hours accessibility
- Integration of ACT in additional treatment options and thus access to all evidence-based therapies and therapy programs as well as necessary social measures
- Continuity of care across settings.

In the conception of ACT in the Hamburg Model the “traditional ACT” has been modified to “therapeutic ACT” (Figure 4 and Table 2).

**Table 2: Differences between traditional ACT and therapeutic ACT**

| **Structure** | **Traditional ACT** | **Therapeutic ACT** |
| --- | --- | --- |
| **Indication** | Severe mental illness | Psychosis |
| **Team** | Non-specialised team | Psychosis expert team |
| **Provider/consumer ratio** | 1:15/20 | 1:15/20 |
| **Availability** | 24h/day / 365 days | 24h/day / 365 days |
| **No-drop-out policy** | Yes | Yes |
| **Pharmacotherapy** | Often no responsibility or not specially trained | Experts |
| **Psychotherapy** | Not specially trained | Experts |
| **Tasks** | Case Management  Home Treatment | Case Management  Home Treatment  Psychotherapy |

As presented in table 2, there are substantial differences between traditional and therapeutic ACT. These are:

- The ACT team is specialised in one illness
- The ACT team is multiprofessional and consists of psychosis experts
- The team has expertise in the pharmacotherapy of psychotic disorders
- The team is specially trained in psychotherapy for psychosis

The tasks also include other evidence-based interventions for severely ill psychosis patients, including Home Treatment and intensive Care Management. With this specialization it is attempted to implement a maximum of disorder-specific treatment quality.

The team at the UKE is multiprofessional and consists of specialists and assistant doctors, psychologists and nursing staff. On the one hand, it is integrated into the Psychosis Outpatient Center, on the other hand it is connected to the inpatient unit and all psychiatrists who participate in the integrated care. As part of the integrated care, each patient is treated by a team consisting of a therapist from the ACT team and a doctor (from the ACT team or resident) over the entire time. The team is responsible for the structural and contentual coordination of the therapy. The therapy is planned together with the patient and, whenever possible, also together with the relatives. The patient can use all therapies offered in the psychosis center. This ensures the access to all evidence-based group therapies and therapy programs as well as necessary social measures. Moreover, the small provider/consumer ratio allows an early access to psychotherapy, which can be performed by the respective ACT therapist. Within the frame of the contract, the private psychiatrists (currently 20) have committed themselves to treat patients without waiting time, more intensive and in close reconciliation with the respective therapist. Hence, the ACT team has the following tasks:

- High frequency and long-term treatment including acute and long-term therapy and including a pharmacotherapy of high quality
- Prevention of relapse with crisis intervention for 7 days a week and 24 hours a day, immediate relapse treatment, immediate compliance-promoting measures in case of non-compliance and immediate re-engagement in case of total treatment discontinuation
- Post-inpatient intensive aftercare und thus reduction of the length of stay
- Planning and coordination of all interventions und thus access to all evidence-based group therapies and therapy programs as well as necessary social measures
- Early access to psychotherapy, which is performed by an ACT member
- Inclusion of private psychiatrists

By this time, 20 private psychiatrists have declared their joining to the Hamburg Model. They have committed themselves to treat patients of integrated care more intensive and without waiting time. In addition to the guideline-based treatment of psychotic disorders including pharmacotherapy, further services are provided to fulfil the contractual objectives. These include:

- Information of the patient about the new concept of care
- Short-dated arranging of appointments
- Coordination of the individual treatment with other providers of the integrated care
- Additional documentation within the framework of integrated care for quality assurance
- Regular participation in the intersectoral case meetings including the preparation of case reports.

**2.5 Description of the planned experimental procedure**

The present study is conducted by the study center of the Psychosis Center at the University Medical Center Hamburg-Eppendorf. The duration of the treatment is unlimited and applies to the entire sample of the treatment in integrated care and ACT. In Germany, Assertive Community Treatment (ACT) has only been implemented at the UKE so far. ACT means that a team of psychosis experts provide acute and long-term treatment in the domestic environment of the patient. The team at the UKE is multiprofessional und consists of specialists and assistant doctors, psychologists and nursing staff. On the one hand, it is integrated into the Psychosis Outpatient Center, on the other hand it is connected to the inpatient unit and all psychiatrists who participate in the integrated care. The team is responsible for the structural and contentual coordination of the therapy. The therapy is planned together with the patient and, whenever possible, also together with the relatives. The patient can use all therapies offered in the psychosis center. This ensures the access to all evidence-based group therapies and therapy programs as well as necessary social measures. Moreover, the small provider/consumer ratio allows an early access to psychotherapy, which can be performed by the respective ACT therapist. Within the frame of the contract, the private psychiatrists (currently 20) have committed themselves to treat patients without waiting time, more intensive and in close reconciliation with the respective therapist.

All patients of the integrated care are examined by a trained psychologist at the times of measurement shown in table 1.

**3.1 Inclusion and Exclusion Criteria**

Patients who fulfil the indications for the participation in the Hamburg Model are included in the present study after signing the consent (see above, please).

Study Protocol

| **Assessments and Measures** | **Details** |
| --- | --- |
| **Fidelity of the ACT team** | Fidelity of the assertive community treatment model was assessed yearly with the Dartmouth Assertive Community Treatment Scale.^[32]^ At initiation of ACCESS, the total score was 4.5 and varied yearly between 4.2 and 4.6 points, indicating that fidelity of the treatment model was good. |
| **Fidelity of ratings** | Trained raters independent of the treatment team to avoid bias. All raters received extensive training, particularly for SCID-I interviews, BPRS, CGI-S, and GAF. |
| **Assessment time points** | Baseline, week 6, and months 3, 6, 12, 18, 24, 30, 36, 40, and 48 |
| **Diagnoses** | Diagnoses of the psychotic disorder and comorbid Axis I disorder(s) were assessed with the SCID-I.^[30]^ |
| **Service disengagement** | Service disengagement for non-practical reasons was considered to be present if a patient repeatedly refused further treatment despite the need and several attempts at reengagement (phone calls to patient and potentially home visits by the assertive community treatment team).^[10]^ |
| **Service use data** | Treatment contacts consisted of face-to-face meetings as well as emails/letters, telephone calls, and contact with institutions or family members. Furthermore, hospital days (inpatient and day-clinic treatment) were noted for each year of treatment. All service use data are presented for patients being actively treated in each year (i.e., excluding service-disengaged patients). |
| **Baseline assessments** | - Sociodemographic, functional, and pretreatment characteristics using the German version of the Early Psychosis File Questionnaire,^[33]^ - Employment/occupation using the Modified Vocational Status Index^[34]^ and the Modified Location Code Index.^[34]^ “Employed/occupied” comprised paid or unpaid full- or part-time employment, being an active student in university, a full- or part-time volunteer; “independent living” comprised living alone, with a partner, or with peers. - Duration of untreated psychosis with the Duration of Untreated Psychosis Scale.^[35-37]^ - Prevalence of previous inpatient treatment, lifetime involuntary admission, and admission within the 2 years before ACCESS were assessed by interviewing patients, relatives, and health service staff previously responsible for the patient. Data were validated by cross checking the hospital database. Involuntary admissions were due to danger to self or others. - Medication adherence was assessed using the criteria of Kane et al.^[38]^ Therapists rated their patients as being fully adherent in the last 4 weeks if taking ≥ 80% of their prescribed medications, partially adherent when taking 20%–80%, and nonadherent when taking ≤ 20% of the prescribed medications. |
| **Baseline and follow-up assessments** | - Psychopathology using the BPRS at baseline and every 6 months - Severity of illness using the Clinical Global Impressions -Severity of Illness scale (CGI-S)^[39]^ - Level of functioning using the Global Assessment of Functioning (GAF) Scale^[40]^; - Quality of life using the 18-item Quality of Life Enjoyment and Satisfaction Questionnaire (QLES-Q-18)^[41]^ - Patients’ satisfaction with their care using the Client Satisfaction Questionnaire (CSQ-8)^[42]^ - Medication adherence (see previous paragraph above)^[38]^ |
