# Supplementary material for: Effectiveness of integrated care including therapeutic assertive community treatment in severe schizophrenia-spectrum and bipolar I disorders: Four-year follow-up of the ACCESS II study
Source: PLoS One. 2018 Feb 27;13(2):e0192929. doi: 10.1371/journal.pone.0192929 (PMC5828355; doi:10.1371/journal.pone.0192929)
Supplement: S5 File — (DOCX) [file pone.0192929.s005.docx]

| [Edit](https://register.clinicaltrials.gov/prs/app/template/edit%2CEditIdentification.vm?wizardmode=Edit&uid=U0001W8A&ts=12&sid=S00044OU&cx=18qimv) | Study Identification   \| Unique Protocol ID: \| ACCESS-II \| \| --- \| --- \| \| Brief Title: \| Integrated Care in Psychotic Disorders With Severe Mental Illness (ACCESS-II) \| \| Official Title: \| Integrated Care in Patients With a Psychotic Disorder Fulfilling Definition of Severe Mental Illness (ACCESS-II Study) \| \| Secondary IDs: \|  \| |
| --- | --- | --- | --- | --- | --- | --- | --- | --- | --- |
|  | |
| [Edit](https://register.clinicaltrials.gov/prs/app/template/edit%2CStatus.vm?wizardmode=Edit&uid=U0001W8A&ts=12&sid=S00044OU&cx=s3k24d) | Study Status   \| Record Verification: \| January 2017 \| \| --- \| --- \| \| Overall Status: \| Recruiting \| \| Study Start: \| May 2007 \| \| Primary Completion: \| January 2018 (ongoing)   [Anticipated] \| \| Study Completion: \| December 2018 (ongoing)  [Anticipated] \| |
|  | |
| [Edit](https://register.clinicaltrials.gov/prs/app/template/edit%2CSponsors.vm?wizardmode=Edit&uid=U0001W8A&ts=12&sid=S00044OU&cx=9nki0m) | Sponsor/Collaborators   \| Sponsor: \| Universitätsklinikum Hamburg-Eppendorf \| \| --- \| --- \| \| Responsible Party: \| Principal Investigator  Investigator: Prof. Dr. med. Martin Lambert [mlambert]  Official Title: Prof. Dr. Martin Lambert  Affiliation: Universitätsklinikum Hamburg-Eppendorf \| \| Collaborators: \|  \| |
|  | |
| [Edit](https://register.clinicaltrials.gov/prs/app/template/edit%2CEditOversightInfo.vm?wizardmode=Edit&uid=U0001W8A&ts=12&sid=S00044OU&cx=-s2298a) | Oversight   \| FDA Regulated?: \| No \| \| --- \| --- \| \| IND/IDE Protocol?: \| No \| \| Review Board: \| Approval Status: Approved     Approval Number: PV4059  Board Name: Ethikkommission der Ärztekammer Hamburg  Board Affiliation: Ethikkommission der Ärztekammer Hamburg Phone: 004940202299240   Email: ethik@aekhh.de \| \| Data Monitoring?: \| No \| \| Plan to Share IPD?: \|  \| \|  \| 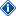NOTE: Plan to Share IPD?: data not entered. \| \| Oversight Authorities: \| Germany: Ethics Commission \| |
|  | |
| [Edit](https://register.clinicaltrials.gov/prs/app/template/edit%2CSummaryInfo.vm?wizardmode=Edit&uid=U0001W8A&ts=12&sid=S00044OU&cx=-g07081) | Study Description   \| Brief Summary: \| The study examine the effectiveness of an integrated care program including therapeutic assertive community treatment (ACT) for people with psychotic disorders fulfilling severe and persistent mental illness (SPMI, ACCESS-II study). \| \| --- \| --- \| \| Detailed Description: \| The trial is carried out at the Psychosis Center of the Department of Psychiatry and Psychotherapy at the University Medical Center Hamburg-Eppendorf (UKE), which has the responsibility to treat all adult psychosis patients within its catchment area of approximately 300.000 habitants.  The IC model was implemented into a network of the Psychosis Center of the University hospital (UKE), private psychiatrists of the UKE catchment area and other outpatient facilities. As such, following health care structures are part of the model and could be used by each patients and its relatives according to need:   - Specialized inpatient unit with attached day-clinic for psychotic disorders; - Acute inpatient unit of the Department; - Specialized psychosis outpatient center separated into a schizophrenia-spectrum and bipolar outpatient teams with a variety of diagnosis-specific evidence-based individual- and group therapies, low-threshold offers to promote day structure (e.g., cooking, sport or art groups), an early detection and intervention service with a mobile early detection team with professionals from the child- and youth and adult psychiatry, possibility of peer-to-peer and relatives-to-relatives counseling, relative groups, etc.; - A multidisciplinary assertive community treatment (ACT) team, which was basically implemented according to guidelines of the Assertive Community Treatment Association and according to the Dartmouth Assertive Community Treatment Scale (DACTS). However, compared to the traditional ACT model there are two important differences: (1) While traditional ACT models have a responsibility for SPMI patients independent from the underlying diagnosis, the present model solely focus on patients with psychotic disorders; (2) The fidelity of the ACT team was further increased by composing a team of highly educated psychosis experts (i.e. consultant psychiatrists, psychiatrists, psychologists, nurses, social worker). Members of the ACT team received diagnosis-specific training in pharmacotherapy, cognitive behavioral (CBT), dynamic, and/or family psychotherapy. - A specialized day-clinic for first-episode psychosis patients in the age range of 15-29 years managed by the child- and youth and adult psychiatry; - A working support outpatient center with outpatient and day-clinic treatment facilities; - 20 private psychiatrists participate in the treatment network of the IC model (their duties and responsibilities are described below). - Further participants of the network are: a psychiatric nursing service, a specialized housing support service and a specialized psychosis living unit.   Within this network, the main responsibility for the individual patient and his relatives is generally allocated according to need and preferences to two specific members of the ACT team, of which one has to be a psychiatrist responsible for pharmacotherapy. However, when pharmacotherapy is stable and adherence assured, private psychiatrists are allowed to take over this responsibility. In this case, the specific IC contract with the private psychiatrists includes several quality assurance guidelines.  Briefly, the staff/client caseload ratio of a single full time employed ACT team member is 1:15-25. In any case, the first responsibility of the network is a broad assessment of the psychotic disorder, comorbid psychiatric and somatic disorders, traumatic events, previous service engagement and medication adherence, social problems, etc. Thereby, a need-adapted treatment plan will be developed together with the patient, relatives and other caregivers. If the patient starts IC during inpatient, transition from in- to outpatient care will be organized. Participant, relatives and other caregiver receive two telephone numbers: one of the ACT therapists for all contacts within office hours (Monday to Friday from 8 am to 6 pm) and a 24 hours crisis number for all emergencies or questions outside office hours. The primary ACT/private psychiatrists subteam is responsible for high-frequent face-to-face treatment contacts fostering "open-end" continuity of care, coordinating treatment and social support (case management), promoting and ensuring service engagement and medication adherence, and offering intensive need-adapted individual psychotherapy at the earliest possible time point. Crisis intervention is offered at any time and is always conducted by two ACT members of which one is the primary ACT therapist. Access to other need-adapted and diagnosis-specific evidence-based interventions is organized within the specialized psychosis outpatient center or the work support center, e.g. psychoeducation groups, social skills training, meta-cognitive training, bipolar psychotherapy groups, etc. Further, all low-threshold interventions in the outpatient center could be used at any time. Thereby day-clinic equivalent program could be organized while being in outpatient status. As many of these patients are at risk for service disengagement and medication non-adherence, a strict 'no-drop-out' policy is implemented including all admissions out of the catchment area.  Managed Care Briefly, the UKE is the managed care organization for the IC model and has full financial responsibility. The UKE receives a yearly per-patient rate, which was calculated according to real direct health care costs of the individual patient including inpatient-, day-clinic- and outpatient treatment. More then 20% of this rate is caused by 'hospital hopping' and repeated emergency room visits. Within one year the calculated yearly per-patient rate now includes: (1) all inpatient days, (2) all day-clinic, (3) all interventions by the ACT team, (4) all interventions in the Specialized psychosis outpatient center, (5) all interventions by private psychiatrists (they receive 4-times higher finances per-patient per-year from the overall yearly per-patient rate), (6) all psychotherapeutic interventions, (7) all assessments to insure quality of outcome, and (8) all managed care facilities. There two specific arrangements: (1) the IC model starts financially at the first day of admission to hospital. This arrangement supports a quick enter into the new IC model and reduced the financial risks for the health insurances. (2) One specific guideline within the model is to stop hospital hopping. To foster this guideline, the UKE has to pay from the yearly per-patient rate every admission in a hospital outside the network from the 4th day.  Assessments and measures Assessments are carried out at baseline, and at 6 weeks, 3, 6, 12, 18, 24, 30, 36, 42, and 48 months follow-ups. At baseline, following variables were assessed: 1) Diagnosis of the psychotic disorder and co-morbid AXIS I disorder(s) with the Structured Clinical Interview for DSM-IV Axis I disorders (SCID-I). In case of clinical evidence for a co-morbid AXIS II disorder, a SCID-II interview for DSM-IV Personality Disorder was applied40; 2) demographic and illness characteristics were assessed with the German version of Early Psychosis File Questionnaire (EPFQ) including age, gender, marital status, years of school education, completed professional education, employment/occupation at entry, ability of independent living, traumatic events, family history of psychiatric illness, previous suicide attempts, previous hospital admissions including involuntary admissions, insight into illness and forensic history. Duration of untreated prodrome, psychosis (DUP) and untreated illness (DUI) will be assessed with the DUP scale). Medication non-adherence was defined according to Robinson et al. as failure to take medication for 1 week or longer. This definition was chosen because stopping medication for a week clearly indicates a problem with acceptance of pharmacological treatment (as opposed of just forgetting a dose).  At baseline and all follow-up time points following structured assessment are applied: 1) Psychopathology with the Brief Psychiatric Rating Scale (BPRS); 2) severity of illness with the Clinical Global Impression Scale (CGI); severity of illness of affective psychosis with the Clinical Global Impression Scale-Bipolar version (CGI-BP); 3) level of functioning with the Global Assessment of Functioning Scale (GAF), 4) Quality of life with the Quality of Life Enjoyment and Satisfaction Questionnaire (Q-LES-Q-18). The Q-LES-Q-18 is a self-report instrument scored on a 5-point scale ('not at all or never' to 'frequently or all the time') with higher scores indicating better enjoyment and satisfaction with specific life domains. The global QOL index is the average score of all 18 items; a score of 4.1 points characterize a quality of life comparable with healthy controls; 5) Patients' satisfaction with care with the Client Satisfaction Questionnaire (CSQ-8). The CSQ-8 is a 8-item instrument that is scored from 1 to 4. The total score ranges from 8 to 32; the mean satisfaction score is computed with a minimum score of 1 and a maximum of 4.  Service disengagement was present, if a patient repeatedly refuses further treatment despite need and several attempts of reengagement (phone calls of patient and potentially home visits of the ACT team). Service used data were assessed from the official IC database, which covers inpatient admissions, day-clinic admissions, and treatment contacts by the ACT team, in the psychosis outpatient center or by the private psychiatrists. \| |
|  | |
| [Edit](https://register.clinicaltrials.gov/prs/app/template/edit%2CConditions.vm?wizardmode=Edit&uid=U0001W8A&ts=12&sid=S00044OU&cx=-i00zpl) | Conditions   \| Conditions: \| Schizophrenia Schizophreniform Disorder Schizoaffective Disorder Delusional Disorder Psychotic Disorder NOS Bipolar Disorder Severe Major Depression With Psychotic Features \| \| --- \| --- \| \| Keywords: \| Schizophrenia Bipolar disorder Assertive community treatment Integrated care Severe and persistent mental disorder \| |
|  | |
| [Edit](https://register.clinicaltrials.gov/prs/app/template/edit%2CIntStudyDesign.vm?wizardmode=Edit&uid=U0001W8A&ts=12&sid=S00044OU&cx=-eu86tl) | Study Design   \| Study Type: \| Interventional   [[Change...](https://register.clinicaltrials.gov/prs/app/template/edit%2CChangeStudyType.vm?uid=U0001W8A&ts=12&sid=S00044OU&cx=4t7cx9)] \| \| --- \| --- \| \| Primary Purpose: \| Treatment \| \| Study Phase: \| Phase 4 \| \| Intervention Model: \| Single Group Assignment \| \| Number of Arms: \| 1 \| \| Masking: \| Open Label \| \| Allocation: \| N/A \| \| Endpoint Classification: \| Efficacy Study \| \| Enrollment: \| 400 [Anticipated] \| |
|  | |
| [Open](https://register.clinicaltrials.gov/prs/app/template/edit%2CArmsGroupsInterventions.vm?wizardmode=Edit&uid=U0001W8A&ts=12&sid=S00044OU&cx=u8ce4i) | Arms and Interventions   \|  \| \| **Arms** \| **Assigned Interventions** \| \| --- \| --- \| \| Experimental: Integrated care  The IC model was implemented into a network of the Psychosis Center of the University hospital (UKE), private psychiatrists of the UKE catchment area and other outpatient facilities. Integrated Care involves ACT treatment within this network. Patients have access to all evidence-based interventions according to need. \| Integrated care  The IC model was implemented into a network of the Psychosis Center of the University hospital (UKE), private psychiatrists of the UKE catchment area and other outpatient facilities. Integrated Care involves ACT treatment within this network. Patients have access to all evidence-based interventions according to need. \| \| \| --- \| --- \| --- \| --- \| --- \| --- \| |
|  | |
| [Edit](https://register.clinicaltrials.gov/prs/app/template/edit%2CEditOutcomeMeasures.vm?wizardmode=Edit&uid=U0001W8A&ts=12&sid=S00044OU&cx=-hotzhm)   [Reorder](https://register.clinicaltrials.gov/prs/app/template/edit%2CReorderOutcomeMeasures.vm?wizardmode=Edit&uid=U0001W8A&ts=12&sid=S00044OU&cx=-sw67vh) | Outcome Measures   \|  \| Primary Outcome Measure:  1.Time to service disengagement  [Time Frame: 4 years] [Safety Issue: Yes]  This primary aim was chosen because the assertive approach of ACT is to prevent service disengagement and because service disengagement is a major predictor for relapse and thereby a poor long-term outcome. Service disengagement is present, if a patient repeatedly refuses further treatment despite need and several attempts of re-engagement (phone calls of patient and potentially home visits of the ACT team).  Secondary Outcome Measures:   1. Change of functioning as measured with the Global Assessment of Functioning Scale (GAF)   [Time Frame: 4 years] [Safety Issue: No]  Change of functioning from baseline to 4-year endpoint will be measured with the Global Assessment of Functioning Scale (GAF) total score. Change from baseline to endpoint is the secondary endpoint.   1. Change of quality of life as measured with the Quality of Life Enjoyment and Satisfaction Questionnaire (Q-LES-Q-18)   [Time Frame: 4 years] [Safety Issue: No]  Change of quality of life as measured with the Quality of Life Enjoyment and Satisfaction Questionnaire (Q-LES-Q-18). The Q-LES-Q-18 is a self-report instrument scored on a 5-point scale ('not at all or never' to 'frequently or all the time') with higher scores indicating better enjoyment and satisfaction with specific life domains. The global QOL index is the average score of all 18 items; a score of 4.1 points characterize a quality of life comparable with healthy controls;   1. Change of psychopathology as measured with the Brief Psychiatric Rating Scale (BPRS)   [Time Frame: 4 years] [Safety Issue: Yes]  Change of psychopathology from baseline to 4-year endpoint will be measured with the Brief Psychiatric Rating Scale (BPRS) total score. Change from baseline to endpoint is the primary endpoint of the study.   1. Change of satisfaction with care as measured with the Client Satisfaction Questionnaire (CSQ-8)   [Time Frame: 4 years] [Safety Issue: No]  Patients' satisfaction with care with the Client Satisfaction Questionnaire (CSQ-8). The CSQ-8 is a 8-item instrument that is scored from 1 to 4. The total score ranges from 8 to 32; the mean satisfaction score is computed with a minimum score of 1 and a maximum of 4.   1. Service use data   [Time Frame: 4 years] [Safety Issue: No]  Service used data were assessed from the official IC database, which covers inpatient admissions, day-clinic admissions, and treatment contacts by the ACT team, in the psychosis outpatient center or by the private psychiatrists. \| \| --- \| --- \| |
|  | |
| [Edit](https://register.clinicaltrials.gov/prs/app/template/edit%2CEligibility.vm?wizardmode=Edit&uid=U0001W8A&ts=12&sid=S00044OU&cx=ijycti) | Eligibility   \| Minimum Age: \| 12 Years \| \| --- \| --- \| \| Maximum Age: \|  \| \| Gender: \| Both \| \| Accepts Healthy Volunteers?: \| No \| \| Criteria: \| Inclusion criteria  Inclusion criteria for trial participation are identical with the IC model participation criteria and are part of IC contracts with the health insurances. Both, treatment and trial participation were voluntary and needed separate informed consent. Following inclusion criteria will be applied:   - Member of one of the following health insurances: DAK Gesundheit, HEK, IKK Classic, AOK Rheinland/Hamburg (Germany); - Diagnosis of a schizophrenia spectrum disorder (i.e. schizophrenia, schizophreniform disorder, schizoaffective disorder, delusional disorder, or psychotic disorder NOS), bipolar disorder, severe major depression with psychotic features, and substance-induced psychotic disorder, all assessed according to DSM-IV with the Structured Clinical Interview for DSM-IV Axis I Disorders (SCID-I); - Present confinement for hospitalization because of an acute illness state as assessed by a psychiatrists; - Presence of a certain severity of illness as assessed with the Brief Psychiatric Rating Scale (BPRS; 24-item version) with a) BPRS total score > 40 points and b) fulfillment of one of the following sub syndromes: ≥ 6 points on item 10 (hallucinations), ≥ 6 points on item 11 (unusual thought content), ≥ 6 points on item 15 (conceptual disorganization), ≥ 10 points on items 3 plus 4 (depressive-suicidal syndrome), ≥ 6 points on item 4 (suicidality), ≥ 15 points on items 8, 9 plus 21 (manic syndrome), ≥ 15 points on items 6, 12 plus 20 (disruptive behavior syndrome) and ≥ 15 points on items 13, 16 plus 17 (negative syndrome); - Age of ≥ 12 years; - Fulfillment of the SPMI condition according to Ruggeri et al.   Exclusion criteria The only exclusion criterion for treatment participation was a psychotic disorder due to medical condition.  Exclusion Criteria:   - The only exclusion criterion for treatment participation was a psychotic disorder due to medical condition. Patients with mental retardation (defined as IQ lower than 70 points) are able to participate in treatment, but were excluded for analysis. \| |
|  | |
| [Open](https://register.clinicaltrials.gov/prs/app/template/edit%2CContactsLocations.vm?wizardmode=Edit&uid=U0001W8A&ts=12&sid=S00044OU&cx=-gjuf14) | Contacts/Locations   \| Central Contact: \| Martin Lambert, Professor     Telephone:   004940741057670     Email:   [lambert@uke.de](mailto:lambert@uke.de) \| \| --- \| --- \| \| Central Contact Backup: \| Daniel Schöttle, Doctor     Telephone:   004915222816853     Email:   [d.schoettle@uke.de](mailto:d.schoettle@uke.de) \| \| Study Officials: \| Martin Lambert, Professor Study Principal Investigator Universitätsklinikum Hamburg-Eppendorf \| \| [[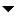](javascript:expandCollapseByKey(false,'_LocationsDivId'))Locations:](javascript:expandCollapseByKey(false,'_LocationsDivId')) \| Germany   \| University Hospital Hamburg-Eppendorf \|  \| Recruiting \| \| --- \| --- \| --- \| \| Hamburg, Hamburg, Germany, 202460 \|  \| \| \| Principal Investigator: Martin Lambert, Professor \| \| \| \| |
|  | |
| [Edit](https://register.clinicaltrials.gov/prs/app/template/edit%2CEditReferences.vm?wizardmode=Edit&uid=U0001W8A&ts=12&sid=S00044OU&cx=ro8fi5) | References   \| Citations: \| **[Study Results]**  Schöttle D, Schimmelmann BG, Karow A, Ruppelt F, Sauerbier AL, Bussopulos A, Frieling M, Golks D, Kerstan A, Nika E, Schödlbauer M, Daubmann A, Wegscheider K, Lange M, Ohm G, Lange B, Meigel-Schleiff C, Naber D, Wiedemann K, Bock T, Lambert M. Effectiveness of integrated care including therapeutic assertive community treatment in severe schizophrenia spectrum and bipolar I disorders: the 24-month follow-up ACCESS II study. J Clin Psychiatry. 2014 Dec;75(12):1371-9. doi: 10.4088/JCP.13m08817.   PubMed ID: 25188752  **[Study Results]**  Lambert M, Bock T, Daubmann A, Meigel-Schleiff C, Lange B, Lange M, Ohm G, Bussopulos A, Frieling M, Golks D, Kerstan A, König HH, Nika L, Ruppelt F, Schödlbauer M, Schöttle D, Sauerbier AL, Rietschel L, Wegscheider K, Wiedemann K, Schimmelmann BG, Naber D, Karow A. [The Hamburg-model of integrated care for patients with psychosis: Part 1. Rationale, treatment concept and results of the pre-study]. Psychiatr Prax. 2014 Jul;41(5):257-65. doi: 10.1055/s-0033-1349497. Review. German.   PubMed ID: 24062155  **[Study Results]**  Karow A, Bock T, Daubmann A, Meigel-Schleiff C, Lange B, Lange M, Ohm G, Bussopulos A, Frieling M, Golks D, Kerstan A, König HH, Nika L, Lange M, Ruppelt F, Schödlbauer M, Schöttle D, Sauerbier AL, Rietschel L, Wegscheider K, Wiedemann K, Schimmelmann BG, Naber D, Lambert M. [The Hamburg-model of integrated care for patients with psychosis: Part 2. Results of the clinical course over 2- and 4-years of treatment]. Psychiatr Prax. 2014 Jul;41(5):266-73. doi: 10.1055/s-0033-1349496. German.   PubMed ID: 24062154 \| \| --- \| --- \| |
